# Redox-active inverse crowns for small molecule activation

**DOI:** 10.1038/s41557-024-01724-5

**Published:** 2025-02-17

**Authors:** Johannes Maurer, Lukas Klerner, Jonathan Mai, Hannah Stecher, Stefan Thum, Michael Morasch, Jens Langer, Sjoerd Harder

**Affiliations:** Inorganic and Organometallic Chemistry, Friedrich-Alexander-Universität Erlangen-Nürnberg, Erlangen, Germany

**Keywords:** Organometallic chemistry, Organometallic chemistry, Supramolecular chemistry

## Abstract

Cyclic crown ethers bind metal cations to form host–guest complexes. Lesser-known inverse crowns are rings of metal cations that encapsulate anionic entities, enabling multiple deprotonation reactions, often with unusual selectivity. Self-assembly of a cycle of metal cations around the multiply charged carbanion during the deprotonation reaction is the driving force for this reactivity. Here we report the synthesis of a pre-assembled inverse crown featuring Na^+^ cations and a redox-active Mg^0^ centre. Reduction of N_2_O followed by N_2_ release and subsequent encapsulation of O^2^^−^ demonstrates its reduce-and-capture functionality. Calculations reveal that this essentially barrier-free process involves a rare N_2_O^2^^−^ dianion, embedded in the metalla-cycle. The inverse crown can adapt itself for binding larger anions like N_2_O_2_^2^^−^ through a self-reorganization process involving ring expansion. The redox-active inverse crown combines the advantages of a strong reducing agent with anion stabilizing properties provided by the ring of metal cations, leading to high reactivity and selectivity.

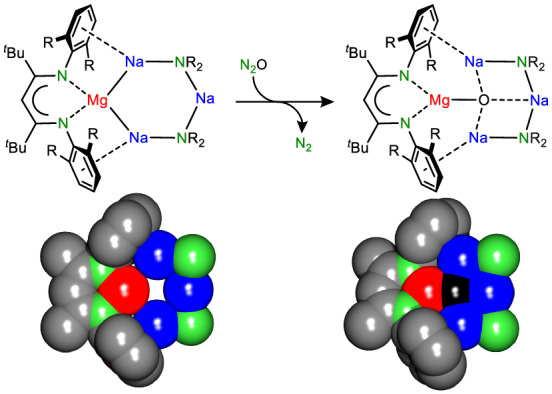

## Abstract (web summary)

Following the building principles of crown ethers for cation encapsulation, inverse crowns are rings of metals that bind anions. Now a redox-active inverse crown ether featuring Na^+^ cations and Mg^0^ has been shown to reduce epoxides, N_2_O, S_8_ or O_2_ by combining anion complexation by the ring of metal cations with the reducing power of Mg^0^.

## Main

**Fig. 1:**
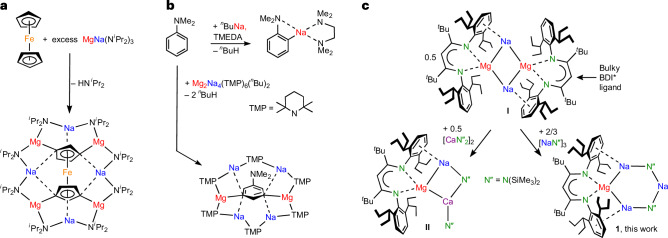
Syntheses of inverse crown complexes. **a**, Selective fourfold 1,1′,3,3′-deprotonation of ferrocene^11^. **b**, The *meta*-selective double deprotonation of *N*,*N*-dimethylaniline^13^ versus directed *ortho* metallation. TMEDA, *N*,*N*,*N*′,*N*′-tetramethylethylenediamine. **c**, Formation of the mixed-metal aggregate **II** from **I** and (CaN′′_2_)_2_ and synthesis of the redox-active inverse crown **1**.

Mixing of metal complexes to achieve unique reactivity dates back to the time of Georg Wittig^1^. Despite this early start, (hetero)bimetallic cooperation is not outdated but is an integral part of contemporary chemistry^2^. Even when restricted to only *s*-block metals, many powerful combinations exist that are fundamental to the development of Turbo-Hauser bases^3,4^, Turbo-Grignard reagents^5,6^ or the ^*n*^BuLi/KO^*t*^Bu superbase^7–9^. Metal cooperation is also the key to Mulvey’s inverse crown complexes^10^. Highlights of this work include the selective fourfold 1,1′,3,3′-deprotonation of ferrocene by MgNa(N^*i*^Pr_2_)_3_ (ref. ^11^; Fig. 1a) and the *meta*-selective double deprotonation of *N*,*N*-dimethylaniline by Mg_2_Na_4_(TMP)_6_(^*n*^Bu)_2_ (TMP, tetramethylpiperidine), which strongly contrasts with the normal *ortho*-directed deprotonation^12,13^ (Fig. 1b). The latter’s abnormal selectivity can be explained by the self-assembly of the inverse crown around Me_2_NC_6_H_3_^2^^−^ during the deprotonation process. With a rare exception of a preformed inverse crown base^14^, the formation of the inverse crown complexes is a dynamic process in which the size of the anion determines composition and ring diameter.

Herein we merge the fields of metallacrowns^15^ and low-valent *s*-block metal chemistry^16–18^ by reporting a preformed inverse crown consisting of Na^+^ cations and a redox-active Mg^0^ centre. The zero-valent nature of the Mg^0^ atom enables the reduction of substrates to give anionic moieties that are encapsulated in an inverse crown complex. The current work is based on our recently reported sodium magnesyl dimer [(BDI*)MgNa]_2_ (**I**; BDI*, bulky β-diketiminate ligand; ref. ^19^; Fig. 1c). Calculations suggest that **I** is best described as being constructed of two (BDI*)Mg^−^ magnesyl anions that are bridged by Na^+^ cations. Supporting the idea that (BDI*)Mg^−^ behaves like an anionic ligand is the observation that the addition of dimeric (CaN′′_2_)_2_ led to exclusive formation of the mixed aggregate (BDI*)MgNa/CaN′′_2_ with a unique Mg–Ca bond (**II**; N′′, N(SiMe_3_)_2_)^20^. As the electronegativity difference of Mg and Ca is only small, some electron transfer from the electron-rich Mg^0^ centre to the Ca^2+^ centre is observed. Herein we report that a mixture of [(BDI*)MgNa]_2_ and NaN′′, which forms a cyclic trimer in the solid state^21^, resulted in exclusive formation of **1** (Fig. 1c), which is shown to be a preformed redox-active inverse crown.

## Results and discussion

### Synthesis and properties of the inverse Mg^0^Na^+^_3_ crown (1)

**Fig. 2:**
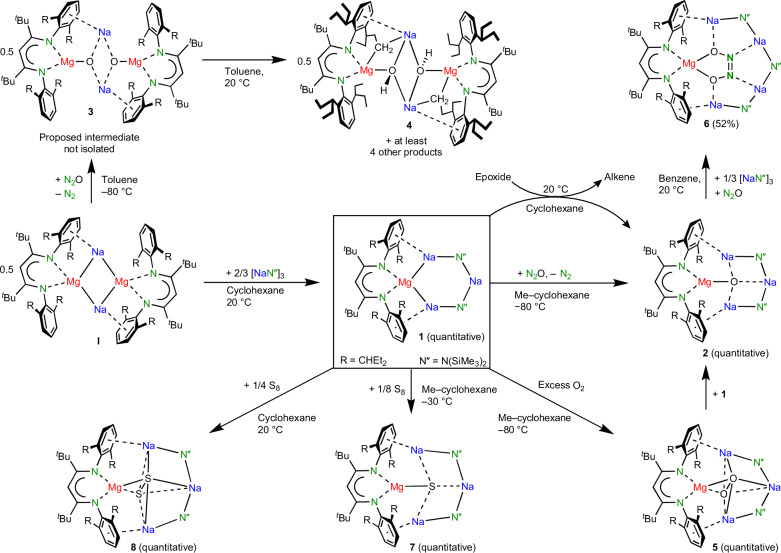
Reactivity of sodium magnesyl complex I and inverse crown 1. Whereas **I** reacted unselectively, reactions with inverse crown **1** are generally quantitative and highly selective on account of anion stabilization by the inverse crown of metals.

Reaction of a dark-brown solution of **I** in methylcyclohexane-d_14_ with 1.33 equiv. trimeric (NaN′′)_3_ led to an immediate colour change to red. A ^1^H NMR analysis and single-crystal X-ray diffraction show exclusive formation of (BDI*)MgNa_3_N′′_2_ (**1**; Fig. 2), which as a crude product is essentially pure. Different **I**/(NaN′′)_3_ ratios repeatedly led to the same product, indicating that cyclic **1** is a privileged structure. Substitution of a second N′′^−^ anion for (BDI*)Mg^−^ is not observed, likely for steric reasons.

**Fig. 3:**
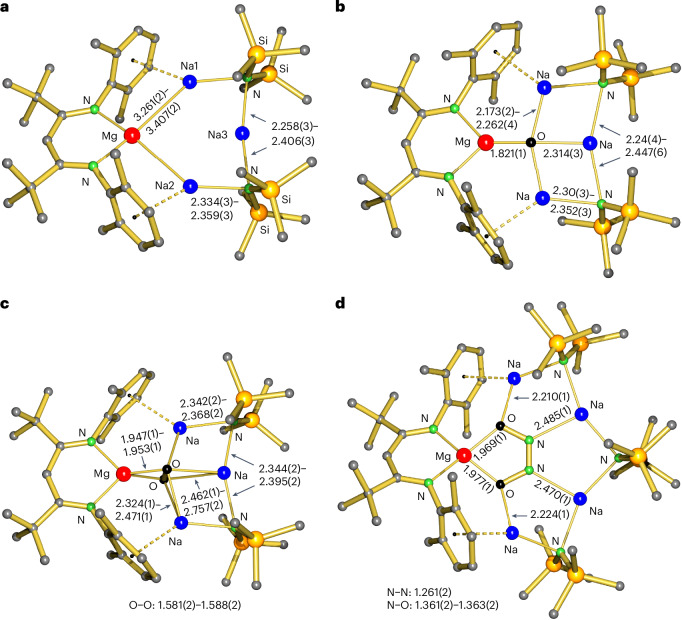
Crystal structures. **a**, (BDI*)MgNa_3_N′′_2_ (**1**). **b**, (BDI*)MgNa_3_N′′_2_O (**2**). **c**, (BDI*)MgNa_3_N′′_2_(O_2_) (**5**). **d**, (BDI*)MgNa_4_N′′_3_(N_2_O_2_) (**6**). The Et groups of the Et_2_(H)C substituents and H atoms are not shown for clarity. Bond distances and ranges are shown in angstroms.

The asymmetric unit in the crystal structure of **1** contains three independent but structurally similar inverse crown aggregates (Fig. 3a). The common feature is the six-membered Mg–Na–N–Na–N–Na ring in which (BDI*)Mg^−^ and N′′^−^ anions are bridged by Na^+^ cations. The Mg–N bonds to the BDI* ligand are unusually long (2.119(3)–2.140(3) Å) and indicative of the low oxidation state on the formally zero-valent Mg centre. Similarly, long Mg–N bonds have been observed in **I** (average, 2.117 Å). The Mg–Na distances vary from 3.261(2) to 3.407(2) Å and are in the range of those in **I** (3.1216(7) and 3.4529(7) Å). The shortest Mg–Na bond is close to the sum of Bragg’s metal radii for Na and Mg (3.19 Å)^22^. Although Na3 is slightly bent inward, the diagonal Mg–Na distances of 3.926(3)–4.396(3) Å are too long to be considered bonding. Additional Ar···Na^+^ contacts are defined by short ring(centroid)···Na^+^ distances (2.626(2)–2.735(2) Å) that are only slightly longer than that in **I** (2.604(1) Å).

Like precursor **I**, solutions of **1** are highly air sensitive and can react with the solvent. A red tetrahydrofuran (THF) solution of **1** immediately turned yellow. Deaggregation likely precedes decomposition. The ^1^H NMR and X-ray diffraction analyses are consistent with the formation of (BDI*)Na(THF)_*x*_, (NaN′′)_3_ and a reactive grey powder that is consistent with Mg^0^ precipitation (Supplementary Figs. 46, 68 and 69). As previously observed^19,23^, such unusual separation of metallic Na^0^ or Mg^0^ from low-valent Mg/Na complexes can be induced by heating or addition of a Lewis base. Also, a red benzene solution of **1** slowly changed colour to yellow under the formation of a white precipitate. The ^1^H NMR analysis showed formation of (BDI*)MgPh, indicating reductive C–H bond activation, the precipitate likely being insoluble metal hydride. However, a cyclohexane-d_12_ solution of **1** even after 3 weeks does not show any signs of decomposition and is also thermally stable up to +60 °C. NMR spectra of **1** in cyclohexane-d_12_ are in accordance with the *C*_2*v*_-symmetric crystal structure. As signals for **I** or (NaN′′)_3_ could not be observed, there is no ion scrambling. By stark contrast, a cyclohexane-d_12_ solution of **I** decomposes at 25 °C within 34 h.

**Fig. 4:**
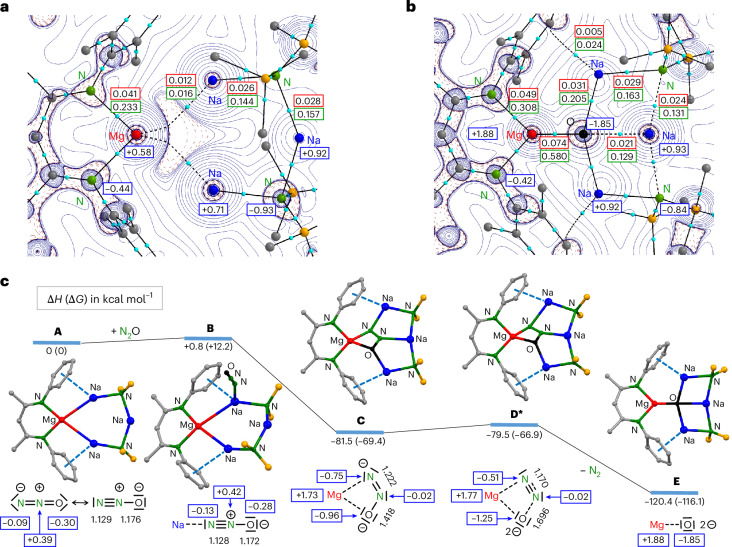
DFT calculations and QTAIM analyses. **a**, Laplacian distribution for (BDI*)MgNa_3_N′′_2_ (**1**) showing bcps (light blue) with *ρ*(**r**) (*e* B^−3^ in red boxes), the Laplacian **∇**^**2**^*ρ*(**r**) (*e* B^−5^ in green boxes) and NPA charges (blue boxes). **b**, Laplacian distribution for (BDI*)MgNa_3_N′′_2_O (**2**). **c**, Energy profile for the reaction of (BDI*)MgNa_3_N′′_2_ (**1**) with N_2_O to give (BDI*)MgNa_3_N′′_2_O (**2**). Δ*H*, the change in enthalpy, and Δ*G*, the change in Gibbs free energy calculated at 298 K (in parentheses) are given in kilocalories per mole, and distances are given in angstroms. NPA charges are shown in blue boxes. **A**, **B**, **C** and **E** are true minima and D* is a transition state.

Density functional theory (DFT) calculations at the B3PW91-D3BJ/def2tzvp//B3PW91-D3BJ/def2svp level of theory reproduced the experimentally observed geometry of **1** (Supplementary Fig. 71). Minor differences are found for the calculated Mg–Na bonds (3.137–3.174 Å), which are shorter than experimentally observed (average, 3.344 Å), and the position of Na3, which is calculated to bend slightly outward instead of inward, demonstrating the flexibility of the ring. The quantum theory of atoms in molecules (QTAIM) confirms Mg–Na bonding by bond paths and bond critical points (bcps) at the Mg–Na axes (Fig. 4a). However, the electron density *ρ*(**r**) and Laplacian **∇**^**2**^*ρ*(**r**) in the bcps are low, indicating a weak electrostatic interaction (**r** = vector space). The Laplacian of the electron distribution shows accumulation of electron density at the Mg nucleus. This is confirmed by natural population analysis (NPA), which calculates a very low charge of +0.58 at Mg that agrees with the formal Mg^0^ assignment. Charges at the connected Na atoms (+0.71/+0.73) are unusually low, whereas the +0.92 charge on the remote Na3 is in the normal range for a Na^+^ cation. This charge distribution is comparable to that in **I** (Mg, +0.57; Na, +0.74) and in line with partial Mg^0^ → Na^+^ electron transfer due to the small difference in their Pauling electronegativities (Mg, 1.31; Na, 0.93)^24^.

### Reactivity of the inverse Mg^0^Na^+^_3_ crown (1)

The redox reactivity of (BDI*)MgNa_3_N′′_2_ (**1**) was evaluated in the reduction of N_2_O. This potent greenhouse gas, responsible for global warming and ozone depletion, is thermodynamically a strong oxidizing agent, but high kinetic stability hampers its use^25,26^. Saturating a dark-red solution of **1** in methylcyclohexane-d_14_ at −80 °C with N_2_O led to an immediate colour change to yellow. The ^1^H NMR analysis confirmed the quantitative formation of an essentially pure crude product that was identified as the expected product of oxidation: (BDI*)MgNa_3_N′′_2_O (**2**; Fig. 3b). The geometry of **2** shows strong similarities with that of **1**. The magnesium centre is oxidized to Mg^2+^ and the O^2^^−^ anion is encapsulated in the inverse Mg^2+^Na^+^_3_ crown. Although the Mg···Na distances (3.182(2)–3.22(2) Å) are shorter than those in **1**, due to loss of two valence electrons at Mg, there is no bonding. This is confirmed by QTAIM analysis of the calculated structure (Fig. 4b), which does not show Mg–Na bond paths. Instead, the complex is stabilized by Ar···Na bonding, for which bond paths were observed. The crystal structure shows short ring(centroid)···Na^+^ distances of 2.662(2) to 2.831(17) Å.

The Na–O distances in **2** vary from 2.173(2) to 2.314(3) Å and are much longer than the Mg–O bond of 1.821(1) Å, which is among the shortest reported. It is significantly shorter than that of 1.8673(9) Å in a Mg_2_Na_2_(TMP)_4_O inverse crown complex with a comparable tetra-coordinated μ_4_-O^2^^−^ anion^27^. Despite the four-fold coordination, it is of similar length to those in LMg(μ_2_-O)MgL complexes (L, anionic ligand) in which O^2−^ is only two-coordinated (Mg–O, 1.8080(5)–1.8380(6) Å)^28,29^. The prominence of the Mg–O bond in **2** is supported by QTAIM analysis (Fig. 4b): the electron density in the Mg–O bcp is two to three times larger than that in the Na–O bcps. The positive Laplacian of +0.58 is in line with a highly ionic Mg^2+^O^2^^−^ bond, which is supported by high NPA charges at Mg (+1.88) and O (−1.85). Complex **2** could be considered a hitherto unobserved (BDI*)MgO^−^ anion that is stabilized by a [Na–N′′–Na–N′′]^+^ chain.

The importance of product stabilization is illustrated by the strikingly different reactivities of **1** and **I** with N_2_O. Whereas **1** cleanly reacted by O incorporation to give **2**, reaction of **I** gave a complex mixture of products, of which one was identified as complex **4** (Supplementary Fig. 1). Tentatively, formation of **4** can be realized by prior formation of the intermediate [(BDI*)MgO^−^Na^+^]_2_ (**3**). Lacking stabilization by an inverse crown arrangement, **3** decomposes instantaneously, for example, by deprotonation of the Et_2_CH substituent to give **4**. Interestingly, this substituent is deprotonated in a terminal Me group and not in the more acidic benzylic position. Similar uncommon selectivity has been noticed previously for inverse crown complexes^13^ (compare with Fig. 1b). The difference in stability of **2** and **3** illustrates the importance of the inverse crown template in **2**. An alumoxane anion (LAlO^−^, where L is a dianionic bis-amide ligand) that is similar to the (BDI*)MgO^−^ anion in **3** was recently isolated in the form of a dimeric K salt, [(LAlO^−^)K^+^]_2_ (ref. ^30^). Its higher stability is explained by the lower polarity of the Al–O bond compared to Mg–O and tight LAlO^−^···K^+^ contacts. Removal of K^+^ led to a similar internal alkyl deprotonation. Also, Jones’s Mg^I^ complex (^DIPP^BDI)MgMg(^DIPP^BDI) (^DIPP^BDI, HC[C(Me)N(DIPP)]_2_; DIPP, 2,6-diisopropylphenyl) reacted unselectively with N_2_O, giving analytically impure mixtures of (^DIPP^BDI)Mg(μ_2_-O)Mg(^DIPP^BDI) and the hydroxide complex (^DIPP^BDI)Mg(μ_2_-OH)_2_Mg(^DIPP^BDI) (ref. ^29^).

**Fig. 5:**
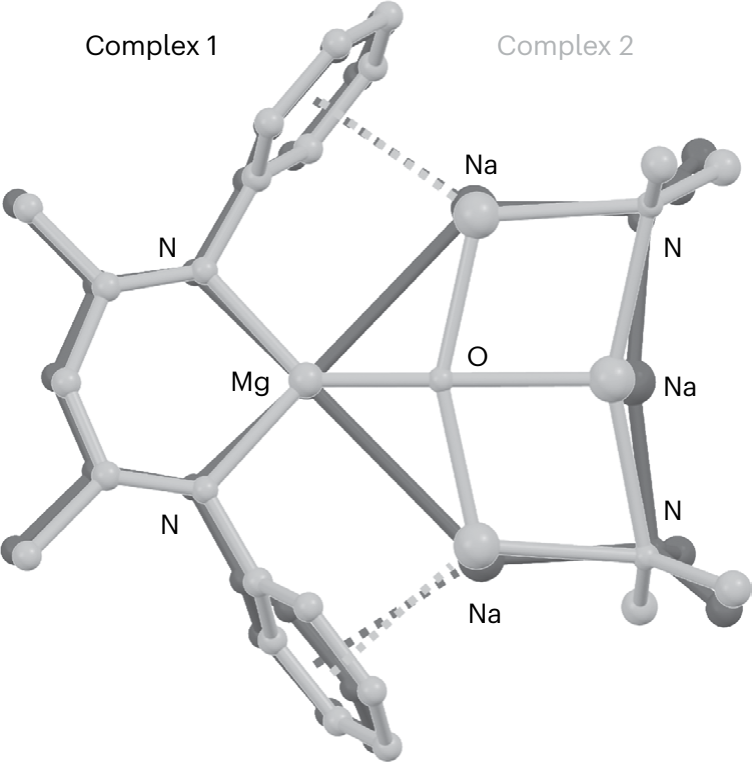
Partial crystal structures of 1 (black) and 2 (light grey). Fitting the structures on top of each other shows that only minimal structural changes occur upon oxidation.

Reaction of **1** with N_2_O could be described as reduce-and-capture reactivity: the product of reduction (O^2^^−^) is captured in the inverse crown. An overlay of the partial structures of **1** and **2** (Fig. 5) shows that the atomic positions hardly change. Thus, the reduction of **1** follows the principle of least motion. Such reactions are generally extremely facile and selective^31^.

The mechanism for the reduction of **1** with N_2_O was studied by DFT calculations (Fig. 4c). Starting with the inverse crown **1** (**A**), coordination of N_2_O is slightly endothermic (**B**), and O=N=N···Na bonding to the Na centre flanking Mg^0^ is slightly favoured over alternative N=N=O···Na bonding, which is 0.3 kcal mol^−1^ higher in energy (**B′** in Supplementary Fig. 73). The key step is the two electron (2*e*) transfer from Mg^0^ to N_2_O, which forms a N_2_O^2^^−^ dianion that is encapsulated within the plane of the inverse crown (**C**). Alternatively, the N_2_O^2^^−^ dianion can also be encapsulated perpendicularly with respect to the MgNa_3_ cycle (**C′** in Supplementary Fig. 73), but this minimum is 21.7 kcal mol^−1^ higher in energy than **C**. The NPA charges (Mg, +1.73; N_2_O, –1.73) and elongated N–N and N–O distances in **C** confirm the formal 2*e* reduction of N_2_O. As this step is extremely exothermic on account of the favourable encapsulation of N_2_O^2^^−^ in the MgNa_3_ cycle (**B** → **C**; change in enthalpy Δ*H* = −82.6 kcal mol^−1^), its transition state is expected to be extremely early and close to **B**. Although we could not locate this state, a transition state starting from the alternative **1**(N_2_O) complex **B′** with N=N=O···Na coordination has been located. As expected, this is a very early transition state with a barrier of only +4.1 kcal mol^−1^. The barrier for subsequent N_2_ release is even lower (**C** → D*; Δ*H* = +2.0 kcal mol^−1^) and results in further energy release (**C** → **E;** Δ*H* = −38.9 kcal mol^−1^). The enormous exothermicity for the reaction, **1** + N_2_O → **2** + N_2_ (Δ*H* = −120.4 kcal mol^−1^), underscores the strongly reducing power of atomic Mg^0^ and the efficient stabilization of O^2^^−^ in the inverse crown complex.

Intermediate **C** with the unusual *N*-nitrosoimide N_2_O^2^^−^ dianion is thermodynamically a highly stable species but kinetically prone to facile N_2_ loss. Note that N_2_O^2^^−^ is isoelectronic to neutral ozone (O_3_) or to carbonite, the dianion of carbon dioxide (CO_2_^2^^−^), which has been proposed to be a crucial intermediate in the reduction of CO_2_ (ref. ^32^). A very early claim for carbonite in the form of a blue-black caesium salt (Cs_2_CO_2_; ref. ^33^) has been corroborated by low-temperature matrix isolation^34^. By contrast, there is no experimental evidence for ionic salts with the N_2_O^2^^−^ dianion. Rare examples of transition metal complexes of the notoriously poor ligand N_2_O include end-on or side-on coordination^34,35^ and, although there is some metal → N_2_O *e* transfer, such complexes should be described with covalent synergistic bonding models^36,37^. The near barrier-free release of N_2_ from N_2_O^2^^−^ in intermediate **C** (Fig. 4c) contrasts with the high kinetic stability of neutral N_2_O. All attempts to optimize any of the intermediates **B**, **C** or D* without the stabilizing Na–N′′–Na–N′′–Na chain, that is, (BDI*)Mg^−^···(N_2_O), led to immediate N_2_ release and the formation of (BDI*)MgO^−^. This illustrates that the crown of metals is able to stabilize intermediates on the pathway.

The inverse crown (BDI*)MgNa_3_N′′_2_ (**1**) also shows highly reducing activity in the deoxygenation of epoxides. Reacting a dark-red solution of **1** in cyclohexane-d_12_ with propylene oxide at 20 °C led to immediate discolouration and quantitative conversion to **2** and propene (Fig. 2). This smooth and selective reaction stands in strong contrast with Wittig’s first report on the deoxygenation of activated epoxides with Ph_3_P, which needed temperatures of at least 200 °C (ref. ^38^). Although **I** also reduced propylene oxide already at room temperature, this led to formation of a large variety of decomposition products. The selective deoxygenation of epoxides with **1** to give **2** and the corresponding alkene is a general reaction and is also smooth for other monosubstituted epoxides like 1-hexylene oxide or styrene oxide (the styrene product partially oligomerized), and for 1,2-disubstituted epoxides like *cis*- and *trans*-stilbene oxides (Supplementary Figs. 50–59). As *cis*- and *trans*-stilbene oxides both reacted to *trans*-stilbene, the reaction is not concerted. We propose reductive C–O bond cleavage to give a Na–C–C–O–Mg intermediate, which then loses alkene to give **2**.

Reaction of **1** with an excess of dry O_2_ selectively led to quantitative formation of the peroxide complex (BDI*)MgNa_3_N′′_2_(O_2_) (**5**). Its crystal structure (Fig. 3c) shows a O_2_^2^^−^ dianion (O–O, 1.581(2)–1.588(2) Å) that is encapsulated in the inverse crown complex by side-on bonding to Mg^2+^ and three Na^+^ cations. While the Mg–O bonds in **5** are in a narrow range (1.947(1)–1.953(1) Å), much more discrepancy exists in the considerably longer Na–O bonds (2.324(1)–2.757(2) Å), indicating that the Mg–O contact is the principal bond. With an average length of 1.948 Å, it is significantly shorter than the average Mg–O bond in the inverse crown complex Mg_2_K_2_N′′_4_(μ_4_-O_2_) (2.013 Å)^39^, and even than that in a (^DIPP^BDI)Mg(μ_2_-O_2_)Mg(^DIPP^BDI) complex (1.961 Å) with only three-coordinated O atoms^29^. The peroxide anion O_2_^2^^−^ in **5** can be further reduced to O^2^^−^ by reaction with **1** to give **2** (Supplementary Information).

In contrast to the highly selective oxidation of **1** to **5**, the oxidation of a MgN′′_2_/NaN′′ mixture with dry air gave poor yields (5–12%) of an inverse Mg_2_Na_2_N′′_4_ crown encapsulating a O_2_^2^^−^/O^2^^−^ mixture in a 32:68 ratio^40^. By comparison, in the very first report of an inverse crown ether complex, obtained after oxidation of a MgN′′_2_/LiN′′ mixture by air, isolated yields of 1–5% were also reported^41^. Oxidation of complex (^DIPP^BDI)MgMg (^DIPP^BDI) with O_2_ gave (^DIPP^BDI)Mg(μ_2_-O_2_)Mg(^DIPP^BDI), which could be isolated only in the form of impure reaction mixtures^29^. Similarly, we reacted the Mg^0^ complex **I** with dry air to obtain product mixtures. We attribute the highly selective oxidation of **1** to peroxide complex **5** to the stabilization of O_2_^2^^−^ in the inverse crown. A recent publication on the role of Ca^2+^ in photosystem II reports a heterometallic Ca/K peroxide complex and shows that alkali metal cations can be decisive for selectivity control^42^.

Reactions of **1** also selectively reduced S_8_, and depending on ratio, temperature and concentration, complexes of S^2^^−^ (**7**) or S_2_^2^^−^ (**8**) could be obtained in near quantitative conversions. Interestingly, reaction of the disulfide complex (**8**) with **1** gave the monosulfide complex **7** in 52% yield. The crystal structures of **7** and **8** (Supplementary Fig. 64) strongly resemble those of their O analogues **2** and **5** (Fig. 3b,c). Efficient encapsulation of these larger S anions without major structural changes shows that the ring is able to stretch itself. Severe disorder, especially in **7**, indicates that S^2^^−^ fits less well in the ring than O^2^^−^. In addition, reaction of the S^2^^−^ complex (**7**) with 0.125 equiv. of S_8_ gave quantitatively the S_2_^2^^−^ complex (**8**).

### Extension of the inverse crown

The efficient stabilization of the O^2^^−^ anion in **2** by the surrounding metal cations prevented further reaction with a second equivalent of N_2_O to give the hyponitrite dianion N_2_O_2_^2^^−^, an onward reactivity that is often observed and generally difficult to control^43^. Although **2** does not react instantaneously with a second equivalent of N_2_O, overnight exposure to an excess of N_2_O led to the formation of several products among which we could identify (BDI*)MgNa_4_N′′_3_(N_2_O_2_) (**6**). Realizing that **6** is an extension of the inverse crown by one NaN′′ unit, the same reaction was performed in the presence of 0.33 equiv. trimeric (NaN′′)_3_. This led to formation of **6** as the major product, which after crystallization could be isolated in 52% yield. The crystal structure of **6** (Fig. 3d) shows the selective formation of *cis*-N_2_O_2_^2^^−^ tightly bound to Mg^2+^ and encapsulated in the crown by four additional contacts to Na^+^ cations. This demonstrates that the six-membered Mg–Na–N–Na–N–Na ring in the original inverse crown **1** can be extended by incorporation of additional NaN′′ units and adapt itself to larger encapsulated anions. Similar dynamic behaviour is typically observed in the self-assembly of inverse crown complexes^10^ (for example, Fig. 1a,b). The facile conversion of O^2^^−^ in **2** to *cis*-N_2_O_2_^2^^−^ by reaction with N_2_O at room temperature stands in stark contrast with the harsh reaction conditions in the synthesis of [Na^+^]_2_[*cis*-N_2_O_2_^2^^−^] from Na_2_O and N_2_O that requires either heating to at least 360 °C (ref. ^44^) or high-energy ball-milling^45^.

## Conclusion

One of the amide anions in trimeric (NaN′′)_3_ can be quantitatively substituted for the magnesyl anion (BDI*)Mg^−^, giving cyclic (BDI*)MgNa_3_N′′_2_ (**1**). As double N′′^−^/(BDI*)Mg^−^ exchange is not observed, the formation of **1** is a highly selective reaction. Complex **1** is stable in alkanes to at least +60 °C, but decomposes in THF or in benzene. The redox-active inverse crown **1** efficiently reduces kinetically stable molecules, like N_2_O, but also epoxides, O_2_ or S_8_, by combining the strongly reducing power of a Mg^0^ centre with the stabilizing effect of a ring of Mg^2+^ and Na^+^ cations for anion complexation. This ring of cations is also responsible for the high selectivity of the reduction process. As it is shown that the ring size can adapt itself to the dimensions of the anion, it is anticipated that such bifunctional inverse crowns can be applied in the reduction of larger entities or the stabilization of unusual anions. Current work focuses on increasing the number of Mg^0^ centres in the ring to achieve multiple-electron transfer and mapping out the boundaries for these strongly reducing host–guest ring systems.

## Methods

### General considerations

All experiments were conducted in dry glassware under an inert nitrogen atmosphere by applying standard Schlenk techniques or glove boxes (MBraun) using freshly dried and degassed solvents. All solvents were degassed with nitrogen, dried over activated aluminium oxide (Innovative Technology, Pure Solv 400-4-MD, Solvent Purification System) and then stored under an inert atmosphere over molecular sieves (3 Å) unless noted otherwise. C_6_D_6_ (Sigma-Aldrich), cyclohexane-d_12_ (Deutero), methylcyclohexane-d_14_ (Deutero), propylene oxide (≥99%, Sigma-Aldrich), styrene oxide (97%, Sigma-Aldrich) and 1,2-epoxyhexane (97%, Sigma-Aldrich) were purchased as indicated, degassed and dried over molecular sieves (3 Å). NaN′′ (95%, Sigma-Aldrich) was washed with hexanes and dried under vacuum. N_2_O (N25, Messer), *trans*-stilbene oxide (99%, Acros Organics), *cis*-stilbene oxide (97%, Sigma-Aldrich) and S_8_ (flakes, ≥99.99%, Sigma-Aldrich) were not further purified.

NMR spectra were measured on Bruker Avance III HD 400 MHz and Bruker Avance III HD 600 MHz spectrometers. Chemical shifts (*δ*) are denoted in parts per million (ppm) and coupling constants, in hertz (Hz). For describing signal multiplicities, common abbreviations are used: s (singlet), t (triplet) and m (multiplet). Spectra were referenced to the solvent residual signal (SiMe_4_ = 0 ppm). Assignments of NMR signals in the ^1^H and ^13^C{^1^H}/^13^C-APT NMR spectra (APT, Attached Proton Test) are based on two-dimensional NMR spectroscopy (Heteronuclear Single Quantum Coherence, Heteronuclear Multiple Bond Correlation, Correlated Spectroscopy).

Elemental analysis was performed with a Hekatech Eurovector EA3000 analyser. Gas chromatography–mass spectrometry measurements were performed on a Thermo Scientific Trace 1310 gas chromatography system (carrier gas, helium) with detection by a Thermo Scientic ISQ LT single quadrupole mass spectrometer. A Phenomenex Zebron ZB-5 column of dimensions 0.25 mm × 30 m with a film thickness of 0.25 μm was used. The samples (1 μl) were injected with an Instant Connect split/splitless (SSL) module in S3 split mode (injector temperature, 280 °C; split ratio, 0.9; carrier gas flow, 1.2 ml min^–1^). Temperature programs were started at 40 °C (hold 1 min) followed by heating ramps, optimized for ideal separation, ending at 330 °C (hold 5 min). Conditions for mass spectrometry were as follows: ion source temperature, 280 °C; ionizing energy, 70 eV; and mass range, 20–500 (mass to charge ratio, *m*/*z*). The molecular identity was confirmed by comparison with entries in the NIST/EPA/NIH Mass Spectral Library (v.2.2, built 10 June 2014).

### Starting materials

The following compounds were synthesized according to literature procedures: [(BDI*)MgNa]_2_ (**I**; ref. ^19^).

### Synthetic procedures

#### Synthesis of (BDI*)MgNa_3_N′′_2_ (1)

[(BDI*)MgNa]_2_ (**I**) (100 mg, 75.6 μmol, 1.0 equiv.) and (NaN′′)_3_ (55.5 mg, 101 μmol, 1.33 equiv.) were suspended in cyclohexane (6 ml), and a colour change from dark brown to dark red was observed upon stirring for 10 min at room temperature. Unreacted solids were filtered off and the filtrate was evaporated to dryness. The obtained reddish oil was stripped with pentane (three times, 6 ml) to quantitatively obtain essentially pure **1** in the form of an orange-red powder. Isolation of the product gave 153 mg of **1** (149 μmol, 99%). Dark-red crystals, suitable for single-crystal X-ray diffraction, were obtained by storing a concentrated solution of **1** in methylcyclohexane at –30 °C. The ^1^H NMR spectra of the raw product and the crystallized product are essentially the same.

The ^1^H NMR results are as follows (cyclohexane-d_12_, 600 MHz, 298 K): *δ* = 7.11–7.10 (m, 4H, *meta-*C**H**_arom_), 6.98–6.95 (m, 2H, *para-*C**H**_arom_), 5.25 (s, 1H, C**H-**backbone), 2.94–2.88 (m, 4H, C(**H**)Et_2_), 2.05–1.97, 1.92–1.84 (two signals; m, 4H, CH(C**H**_**2**_CH_3_)_2_), 1.74–1.63 (m, 8H, CH(C**H**_**2**_CH_3_)_2_), 1.23 (s, 18H, C**H**_**3**_^*t*^Bu), 0.97 (t, ^3^*J*_HH_ = 7.2 Hz, 12H, CH(CH_2_C**H**_**3**_)_2_), 0.93 (t, ^3^*J*_HH_ = 7.4 Hz, 12H, CH(CH_2_C**H**_**3**_)_2_), −0.12 (s, 36H, Si**Me**_**3**_), all in parts per million (ppm).

The ^13^C{^1^H} NMR results are as follows (cyclohexane-d_12_, 151 MHz, 298 K): *δ* = 173.7 (2C, **C**N-backbone), 152.7 (2C, N–**C**_arom_), 140.2 (4C, *ortho*-**C**_arom_), 126.0 (4C, *meta*-**C**_arom_), 121.5 (2C, *para*-**C**_arom_), 96.3 (1C, **C**H-backbone), 44.4 (2C, **C**Me_3_^*t*^Bu), 41.5 (4C, **C**(H)Et_2_), 33.4 (6C, **C**H_3_^*t*^Bu), 27.7, 24.7 (two signals; 4C, CH(**C**H_2_CH_3_)_2_), 12.1, 11.4 (two signals; 4C, CH(CH_2_**C**H_3_)_2_), 7.2 (12C, Si**Me**_**3**_), all in ppm.

Elemental analysis for C_55_H_105_MgN_4_Na_3_Si_4_ (*M* = 1,028.09 g mol^–1^) was as follows: calculated C, 64.26; H, 10.29; N, 5.45%; found C, 64.27; H, 10.20; N, 5.44%.

#### Synthesis of (BDI*)MgNa_3_N′′_2_O (2)

A solution of (BDI*)MgNa_3_N′′_2_ (**1**) (29.5 mg, 28.7 μmol, 1.0 equiv.) in methylcyclohexane-d_14_ (600 μl) was degassed (two times, freeze–pump–thaw) and the vacuum was backfilled with N_2_O at −80 °C. Upon stirring, the dark-red solution changed its colour to yellow, indicating full consumption of **1**. All volatiles were removed in vacuo, and the waxy residue was stripped with pentanes (two times, 3 ml) and washed with hexanes (1 ml), quantitatively yielding essentially pure **2** as an off-white powder. Isolation of the product gave 26.4 mg of **2** (25.3 μmol, 88%). Crystals suitable for single-crystal X-ray diffraction were obtained by storing a saturated solution of **2** in methylcyclohexane at −30 °C.

The ^1^H NMR results are as follows (cyclohexane-d_12_, 600 MHz, 298 K): *δ* = 7.21–7.16 (m, 6H, C**H**_arom_), 5.40 (s, 1H, C**H-**backbone), 2.87–2.82 (m, 4H, C(**H**)Et_2_), 1.98–1.90, 1.89–1.82 (two signals; m, 4H, CH(C**H**_**2**_CH_3_)_2_), 1.75–1.65 (m, 8H, CH(C**H**_**2**_CH_3_)_2_), 1.23 (s, 18H, C**H**_**3**_^*t*^Bu), 1.09 (t, ^3^*J*_HH_ = 7.3 Hz, 12H, CH(CH_2_C**H**_**3**_)_2_), 0.97 (t, ^3^*J*_HH_ = 7.4 Hz, 12H, CH(CH_2_C**H**_**3**_)_2_), –0.18 (s, 36H, Si**Me**_**3**_), all in ppm.

The ^13^C{^1^H} NMR results are as follows (cyclohexane-d_12_, 151 MHz, 298 K): *δ* = 177.2 (2C, **C**N-backbone), 149.2 (2C, N–**C**_arom_), 140.6 (4C, *ortho*-**C**_arom_), 126.4 (4C, *meta*-**C**_arom_), 124.2 (2C, *para*-**C**_arom_), 96.4 (1C, **C**H-backbone), 44.9 (2C, **C**Me_3_^*t*^Bu), 41.8 (4C, **C**(H)Et_2_), 33.0 (6C, **C**H_3_^*t*^Bu), 29.9, 25.3 (two signals; 4C, CH(**C**H_2_CH_3_)_2_), 12.5, 12.1 (two signals; 4C, CH(CH_2_**C**H_3_)_2_), 6.9 (12C, Si**Me**_**3**_), all in ppm.

Elemental analysis for C_55_H_105_MgN_4_Na_3_OSi_4_ (*M* = 1,044.09 g mol^–1^) was as follows: calculated C, 63.27; H, 10.14; N, 5.37%; found C, 63.45; H, 9.90; N, 4.96%.

#### Synthesis of (BDI*)MgNa_3_N′′_2_ (O_2_) (5)

A solution of (BDI*)MgNa_3_N′′_2_ (**1**) (30.0 mg, 29.2 μmol, 1.0 equiv.) in methylcyclohexane (2 ml) was degassed (two times, freeze–pump–thaw) and the vacuum was backfilled with dry air at −80 °C. Upon stirring, the dark-red solution changed its colour to yellow, indicating full consumption of **1**. All volatiles were removed in vacuo, and the residue was washed with pentanes (1 ml), quantitatively yielding essentially pure **5** as an off-white powder. Isolation of the product gave 24.7 mg of **5** (23.3 μmol, 80%). Crystals suitable for single-crystal X-ray diffraction were obtained by storing a saturated solution of **5** in hexanes at −30 °C.

The ^1^H NMR results are as follows (methylcyclohexane-d_14_, 600 MHz, 298 K): *δ* = 7.22–7.17 (m, 6H, C**H**_arom_), 5.35 (s, 1H, C**H-**backbone), 2.91–2.86 (m, 4H, C(**H**)Et_2_), 1.95–1.88 (m, 4H, CH(C**H**_**2**_CH_3_)_2_), 1.75–1.67 (m, 8H, CH(C**H**_**2**_CH_3_)_2_), 1.61–1.57 (m, 4H, CH(C**H**_**2**_CH_3_)_2_), 1.26 (s, 18H, C**H**_**3**_^*t*^Bu), 0.98 (t, ^3^*J*_HH_ = 7.3 Hz, 12H, CH(CH_2_C**H**_**3**_)_2_), 0.94 (t, ^3^*J*_HH_ = 7.4 Hz, 12H, CH(CH_2_C**H**_**3**_)_2_), −0.16 (s, 36H, Si**Me**_**3**_), all in ppm.

The ^13^C{^1^H} NMR results are as follows (methylcyclohexane-d_14_, 151 MHz, 298 K): *δ* = 176.9 (2C, **C**N-backbone), 147.9 (2C, N–**C**_arom_), 140.7 (4C, *ortho*-**C**_arom_), 126.3 (4C, *m*eta-**C**_arom_), 123.7 (2C, *para*-**C**_arom_), 95.9 (1C, **C**H-backbone), 44.5 (2C, **C**Me_3_^*t*^Bu), 41.2 (4C, **C**(H)Et_2_), 32.7 (6C, **C**H_3_^*t*^Bu), 28.2, 24.6 (two signals; 4C, CH(**C**H_2_CH_3_)_2_), 12.0, 11.1 (two signals; 4C, CH(CH_2_**C**H_3_)_2_), 6.5 (12C, Si**Me**_**3**_), all in ppm.

Elemental analysis for C_55_H_105_MgN_4_Na_3_O_2_Si_4_ (*M* = 1,060.09 g mol^–1^) was as follows: calculated C, 62.32; H, 9.98; N, 5.29%; found C, 62.19; H, 10.03; N, 5.60%.

#### Synthesis of (BDI*)MgNa_3_N′′_2_(N_2_O_2_) (6)

A solution of (BDI*)MgNa_3_N′′_2_O (**2**) (31.1 mg, 29.8 μmol, 1.0 equiv.) and (NaN′′)_3_ (5.50 mg, 10.0 μmol, 0.33 equiv.) in C_6_D_6_ (3 ml) was degassed (two times, freeze–pump–thaw) and the vacuum was backfilled with N_2_O at room temperature. The reaction mixture was stirred for 18 h at room temperature, and all volatiles were removed in vacuo. The obtained off-white powder was recrystallized from hexanes at −30 °C, yielding colourless crystals of **6** (19.7 mg, 15.5 μmol, 52%), which were suitable for single-crystal X-ray diffraction.

The ^1^H NMR results are as follows (cyclohexane-d_12_, 600 MHz, 298 K): *δ* = 7.23–7.21 (m, 2H, *para*-C**H**_arom_), 7.17–7.16 (m, 4H, *meta-*C**H**_arom_), 5.67 (s, 1H, C**H-**backbone), 2.90–2.85 (m, 4H, C(**H**)Et_2_), 1.99–1.91, 1.73–1.66, 1.59–1.53, 1.36–1.30 (four signals; m, 4H, CH(C**H**_**2**_CH_3_)_2_), 1.15 (s, 18H, C**H**_**3**_^*t*^Bu), 1.00–0.95 (m, 24H, CH(CH_2_C**H**_**3**_)_2_), −0.07 (s, 36H, Si**Me**_**3**_), −0.09 (s, 18H, Si**Me**_**3**_), all in ppm.

The ^13^C{^1^H} NMR results are as follows (cyclohexane-d_12_, 151 MHz, 298 K): *δ* = 179.0 (2C, **C**N-backbone), 152.0 (2C, N–**C**_arom_), 142.2 (4C, *ortho*-**C**_arom_), 125.3 (4C, *m*eta-**C**_arom_), 123.2 (2C, *para*-**C**_arom_), 98.4 (1C, **C**H-backbone), 45.2 (2C, **C**Me_3_^*t*^Bu), 42.3 (4C, **C**(H)Et_2_), 33.7 (6C, **C**H_3_^*t*^Bu), 28.4, 25.2 (two signals; 4C, CH(**C**H_2_CH_3_)_2_), 12.8, 12.0 (two signals; 4C, CH(CH_2_**C**H_3_)_2_), 7.0 (6C, Si**Me**_**3**_), 6.9 (12C, Si**Me**_**3**_), all in ppm.

Elemental analysis for C_61_H_123_MgN_7_Na_4_O_2_Si_6_ (*M* = 1,271.48 g mol^–1^) was as follows: calculated C, 57.62; H, 9.75; N, 7.71%; found C, 58.17; H, 9.58; N, 7.59%.

#### Synthesis of (BDI*)MgNa_3_N′′_2_S (7)

To a cooled (–30 °C) solution of (BDI*)MgNa_3_N′′_2_ (**1**) (31.1 mg, 30.2 μmol, 1.0 equiv.) in methylcyclohexane (25 ml), S_8_ (1.0 mg, 3.90 μmol, 0.129 equiv.) was added. Upon vigorous stirring, the dark-red solution changed its colour to yellow, indicating full consumption of **1**. The reaction mixture was filtered immediately and the filtrate was dried in vacuo. The waxy residue was stripped with pentanes (2 × 1 ml), quantitatively yielding essentially pure (BDI*)MgNa_3_N′′_2_S (**7**) as an off-white powder. Isolation of the product gave 30.5 mg of **7** (28.8 μmol, 95%). Crystals suitable for single-crystal X-ray diffraction were obtained by storing a saturated solution of **7** in hexanes at −30 °C. Both cooling and high dilution are crucial for quantitative formation of **7**. When the concentration was increased and the reaction was run at room temperature, conversion was unselective, and also (BDI*)MgNa_3_N′′_2_S_2_ (**8**) formed.

The ^1^H NMR results are as follows (cyclohexane-d_12_, 600 MHz, 298 K): *δ* = 7.13–7.05 (m, 6H, C**H**_arom_), 5.33 (s, 1H, C**H-**backbone), 2.90–2.86 (m, 4H, C(**H**)Et_2_), 1.95–1.84, 1.75–1.65 (two signals; m, 8H, CH(C**H**_**2**_CH_3_)_2_), 1.23 (s, 18H, C**H**_**3**_^*t*^Bu), 0.98–0.94 (m, 24H, CH(CH_2_C**H**_**3**_)_2_), −0.16 (s, 36H, Si**Me**_**3**_), all in ppm.

The ^13^C{^1^H} NMR results are as follows (cyclohexane-d_12_, 151 MHz, 298 K): *δ* = 177.2 (2C, **C**N-backbone), 149.5 (2C, N–**C**_arom_), 141.3 (4C, *ortho*-**C**_arom_), 125.9 (4C, *meta*-**C**_arom_), 121.9 (2C, *para*-**C**_arom_), 96.2 (1C, **C**H-backbone), 44.8 (2C, **C**Me_3_^*t*^Bu), 41.6 (4C, **C**(H)Et_2_), 33.1 (6C, **C**H_3_^*t*^Bu), 27.6, 24.8 (two signals; 4C, CH(**C**H_2_CH_3_)_2_), 11.6, 11.3 (two signals; 4C, CH(CH_2_**C**H_3_)_2_), 6.8 (12C, Si**Me**_**3**_), all in ppm.

Elemental analysis for C_55_H_105_MgN_4_Na_3_SSi_4_ (*M* = 1,060.15 g mol^–1^) was as follows: calculated C, 62.31; H, 9.98; N, 5.28%; found C, 62.23; H, 10.38; N, 5.11%.

#### Synthesis of (BDI*)MgNa_3_N′′_2_S_2_ (8)

For procedure A, to a solution of (BDI*)MgNa_3_N′′_2_ (**1**; 31.1 mg, 30.2 μmol, 1.0 equiv.) in cyclohexane-d_12_ (600 μl), S_8_ (2.0 mg, 7.80 μmol, 0.26 equiv.) was added. Upon stirring, the dark-red solution changed its colour to yellow, indicating full consumption of **1**. The reaction mixture was stirred for 16 h and filtered, and the filtrate was dried in vacuo. The waxy residue was stripped with pentanes (2 × 1 ml) and washed with cold (−30 °C) pentanes (500 μl), yielding essentially pure (BDI*)MgNa_3_N′′_2_S_2_ (**8**; 32.0 mg, 29.3 μmol, 90%) as an off-white powder. Crystals suitable for single-crystal X-ray diffraction were obtained by storing a saturated solution of **2** in hexanes at −30 °C.

For procedure B, to a solution of (BDI*)MgNa_3_N′′_2_S (**7**; 30.5 mg, 28.8 μmol, 1.0 equiv.) in cyclohexane-d_12_ (600 μl), S_8_ (1.0 mg, 3.90 μmol, 0.135 equiv.) was added. The reaction mixture was stirred for 16 h and filtered, and the filtrate was dried in vacuo. Work-up according to the same procedure as in A yielded essentially pure (BDI*)MgNa_3_N′′_2_S_2_ (**8**; 29.9 mg, 27.4 μmol, 95%) as an off-white powder.

The ^1^H NMR results are as follows (cyclohexane-d_12_, 600 MHz, 298 K): *δ* = 7.14–7.08 (m, 6H, C**H**_arom_), 5.29 (s, 1H, C**H-**backbone), 3.05–3.02 (m, 4H, C(**H**)Et_2_), 1.94–1.80 (m, 12H, CH(C**H**_**2**_CH_3_)_2_), 1.71–1.65 (m, 4H, CH(C**H**_**2**_CH_3_)_2_), 1.27 (s, 18H, C**H**_**3**_^*t*^Bu), 0.91 (t, ^3^*J*_HH_ = 7.4 Hz, 12H, CH(CH_2_C**H**_**3**_)_2_), 0.86 (t, ^3^*J*_HH_ = 7.3 Hz, 12H, CH(CH_2_C**H**_**3**_)_2_), –0.11 (s, 36H, Si**Me**_**3**_), all in ppm.

The ^13^C{^1^H} NMR results are as follows (cyclohexane-d_12_, 151 MHz, 298 K): *δ* = 177.5 (2C, **C**N-backbone), 148.7 (2C, N–**C**_arom_), 142.0 (4C, *ortho*-**C**_arom_), 126.6 (4C, *meta*-**C**_arom_), 122.1 (2C, *para*-**C**_arom_), 96.3 (1C, **C**H-backbone), 44.9 (2C, **C**Me_3_^*t*^Bu), 41.4 (4C, **C**(H)Et_2_), 32.2 (6C, **C**H_3_^*t*^Bu), 26.7, 24.2 (two signals; 4C, CH(**C**H_2_CH_3_)_2_), 10.9, 10.5 (two signals; 4C, CH(CH_2_**C**H_3_)_2_), 6.9 (12C, Si**Me**_**3**_), all in ppm.

Elemental analysis for C_55_H_105_MgN_4_Na_3_S_2_Si_4_ (*M* = 1,092.21 g mol^–1^) was as follows: calculated C, 60.48; H, 9.69; N, 5.13%; found C, 60.79; H, 10.00; N, 5.11%.

### Crystal structure determination

Suitable single crystals of compounds **1**, **2**, **4**, **5**, **6**, **7**, **8**, (BDI*)Na(THF) and (BDI*)Na(THF)_3_ were embedded in protective perfluoropolyalkyether oil (viscosity, 1,800 cSt; ABCR) on a microscope slide, and a single specimen was selected and subsequently transferred to the cold nitrogen gas stream of the diffractometer.

The intensity data were collected at 100 K using Cu Kα radiation (wavelength *λ* = 1.54184 Å) on an Agilent SuperNova dual radiation diffractometer with microfocus X-ray sources and mirror optics. The measured data were processed with the CrysAlisPro software package from Rigaku Oxford Diffraction using different version for compounds **4** (ref. ^46^), compounds **1** and **2** (ref. ^47^), compounds **5**–**8** (ref. ^48^) and (BDI*)Na(THF)^49^. Data were corrected for Lorentz and polarization effects, and an empirical absorption correction using spherical harmonics as well as a numerical absorption correction based on Gaussian integration over a multifaceted crystal model were applied. Using Olex2 (ref. ^50^), the structures were solved by dual-space methods (SHELXT)^51^ and refined by full-matrix least-squares procedures on *F*^2^ using SHELXL (ref. ^52^). All non-hydrogen atoms were refined with anisotropic displacement parameters. Most H atoms were placed in geometrically calculated positions and refined by using a riding model where each H atom was assigned a fixed isotropic displacement parameter with a value equal to 1.2*U*_eq_ (CH or CH_2_) or 1.5*U*_eq_ (CH_3_) of its parent C atom.

### Computational details

All calculations were carried out using Gaussian 16A (ref. ^53^). All methods were used as implemented. All structures were fully optimized at a B3PW91-GD3BJ/def2svp level of theory, which includes Grimme D3 dispersion correction using Becke–Johnson dampening (GD3BJ)^54–58^. All structures were characterized as true minima (Nimag = 0) or as transition states (Nimag = 1) by frequency calculations on the same level of theory. Energies were determined at a B3PW91-GD3BJ/def2tzvp level of theory. The same level of theory was used for NPA charge calculations with NBO6 (ref. ^59^). QTAIM analysis was carried out using AIMAll (v.17) with the wave functions obtained from the B3PW91-GD3BJ/def2tzvp level of theory^60,61^.

## Online content

Any methods, additional references, Nature Portfolio reporting summaries, source data, extended data, supplementary information, acknowledgements, peer review information; details of author contributions and competing interests; and statements of data and code availability are available at 10.1038/s41557-024-01724-5.

## Supplementary information

Supplementary Information Supplementary synthetic methods, NMR spectra, crystal structure details and details for DFT calculations.

Supplementary Data 1 The *XYZ* coordinates for the optimized geometries of all calculated compounds.

Supplementary Data 2 Crystallographic data for complex 1 (CCDC 2365518).

Supplementary Data 3 Crystallographic data for complex 2 (CCDC 2365519).

Supplementary Data 4 Crystallographic data for complex 4 (CCDC 2365520).

Supplementary Data 5 Crystallographic data for complex 5 (CCDC 2365521).

Supplementary Data 6 Crystallographic data for complex 6 (CCDC 2365522).

Supplementary Data 7 Crystallographic data for complex 7 (CCDC 2387079).

Supplementary Data 8 Crystallographic data for complex 8 (CCDC 2387080).

Supplementary Data 9 Crystallographic data for complex (BDI*)Na(THF) (CCDC 2366895).

Supplementary Data 10 Crystallographic data for complex (BDI*)Na(THF)_3_ (CCDC 2366896).

## Data Availability

All data supporting the findings of this study are available within the Article and its Supplementary Information. Crystallographic data for the structures reported in this Article have been deposited at the Cambridge Crystallographic Data Centre (CCDC) under deposition numbers 2365518 (**1**), 2365519 (**2**), 2365520 (**4**), 2365521 (**5**), 2365522 (**6**), 2366895 ((BDI*)Na(THF)), 2366896 ((BDI*)Na(THF)_3_), 2387079 (**7**) and 2387080 (**8**). These data can be obtained free of charge from the CCDC via www.ccdc.cam.ac.uk/data_request/cif. Spectroscopic data that support the findings of this study as well as complementary crystallographic and computational details are included in the Supplementary Information. Raw data are also available from the corresponding author on reasonable request.
