# Supplementary material for: Redox-active inverse crowns for small molecule activation
Source: Nat Chem. 2025 Feb 17;17(5):703–9. doi: 10.1038/s41557-024-01724-5 (PMC12055576; doi:10.1038/s41557-024-01724-5)
Supplement: Supplementary file 1 — Supplementary synthetic methods, NMR spectra, crystal structure details and details for DFT calculations. [file 41557_2024_1724_MOESM1_ESM.pdf]

nature chemistry

Supplementary information https://doi.org/10.1038/s41557-024-01724-5

# Redox-active inverse crowns for small molecule activation

In the format provided by the authors and unedited

## Table of Contents

1. Additional complex synthesis and reactivity .......... 2
2. Spectroscopic data .......... 6
3. Crystal structure determination .......... 43
4. DFT calculations .......... 57
5. References .......... 60

# 1. Additional complex synthesis and reactivity

### [(BDI*)MgNa]$_2$ (I)

(BDI*)MgI (1.02 g, 1.33 mmol) and Na/NaCl (2.52 g, 5 wt%, 5.50 mmol) were suspended in benzene (30 ml) and stirred at room temperature for 14 h. The resulting dark brown suspension was filtered and the residue was extracted with benzene (2 × 20 ml). The combined filtrate and extracts were evaporated to dryness and the remaining black coloured solid was washed subsequently with hexane (3 ml) and cold pentane (−20 °C, 2 × 1 ml). **I** was obtained as dark brown powder that was dried under a high vacuum (420 mg, 0.318 mmol, 48%).

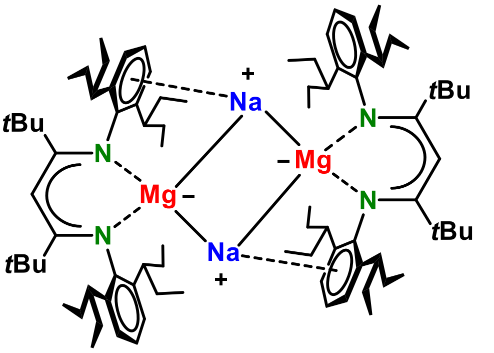


$^{1}$H NMR (600.13 MHz, $C_6D_6$, 298 K): $\delta$ = 1.00–1.03 (m, 48H, $CH_3$), 1.24 (s, 36H, $C(CH_3)_3$), 1.69–1.76 (m, 8H, $CH_2$), 1.80–1.88 (m, 8H, $CH_2$), 1.92–1.98 (m, 8H, $CH_2$), 1.99–2.07 (m, 8H, $CH_2$), 3.04–3.08 (m, 8H, CH), 5.21 (s, 2H, CH-backbone), 6.92–6.96 (m, 4H, CH-arom), 7.00 (d, 7.5 Hz, 8H, CH-arom) ppm. $^{13}$C NMR (150.92 MHz, $C_6D_6$, 298 K): $\delta$ = 11.1 ($CH_3$), 11.7 ($CH_3$), 24.6 ($CH_2$), 25.2 ($CH_2$), 33.4 (C(**C**H$_3$)$_3$), 40.9 (CH), 43.9 (**C**(CH$_3$)$_3$), 95.3 (CH-backbone), 121.3 (C-arom), 125.2 (C-arom), 139.4 (C-arom), 150.8 (C-arom), 172.3 (CN-backbone) ppm. Elemental analysis. Calculated for $C_{86}H_{138}Mg_2Na_2N_4$ ($M$ = 1,322.67 g mol$^{-1}$): C 78.10, H 10.52, N 4.24, Mg 3.68, Na 3.48. Found: C 77.91, H 10.47, N 4.21, Mg 3.63, Na 3.42.

### Alternative route to of (BDI*)MgNa$_3$N''$_2$O (2)

To a solution of (BDI*)MgNa$_3$N''$_2$ (**1**) (31.1 mg, 30.3 $\mu$mol, 1.0 eq) in methylcyclohexane (BDI*)MgNa$_3$N''$_2$(O$_2$) (**5**) (31.1 mg, 29.8 $\mu$mol, 1.0 eq) was added and the resulting suspension was stirred for 16 h at room temperature. All volatiles were removed *in vacuo* and the waxy residue was stripped with pentanes (two times 3 mL). **2** (6.3 mg, 6.05 $\mu$mol, **20%**) was isolated by recrystallization from a concentrated hexanes solution at –30 °C.

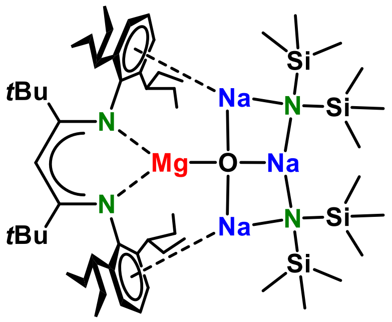


### Alternative route to of (BDI*)MgNa$_3$N''$_2$S (7)

To a solution of (BDI*)MgNa$_3$N''$_2$ (**1**) (31.1 mg, 30.3 $\mu$mol, 1.0 eq) in methylcyclohexane (BDI*)MgNa$_3$N''$_2$(S$_2$) (**8**) (33.0 mg, 30.2 $\mu$mol, 1.0 eq) was added and the resulting solution was stirred for 20 min at room temperature. All volatiles were removed *in vacuo.* **7** (32.0 mg, 30.2 $\mu$mol, **50%**) was isolated by recrystallization from a concentrated hexanes solution at –30 °C.

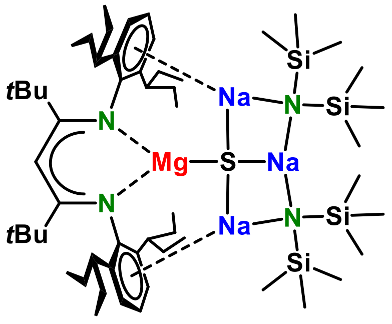

**Attempted synthesis of [(BDI*)MgO$^-$Na$^+$]$_2$ (3) leading to the isolation of 4**

A solution of [(BDI*)MgNa]$_2$ (**I**) (31.3 mg, 23.7 $\mu$mol) in toluene-$d_8$ (500 $\mu$L) was degassed (2 x freeze-pump-thaw) and the vacuum was backfilled with $N_2O$ at –80 °C. Upon stirring, the dark brown solution immediately changed its color to yellow, indicating full consumption of **I**. $^1$H NMR spectroscopy (**Figure 48**) suggested the formation of at least five different products, from which only complex **4** could be identified by single crystal X-ray diffraction (**Figure 1**). All attempts to isolate **4** as a pure compound failed, due to the simultaneous precipitation of other products upon crystallization attempts.

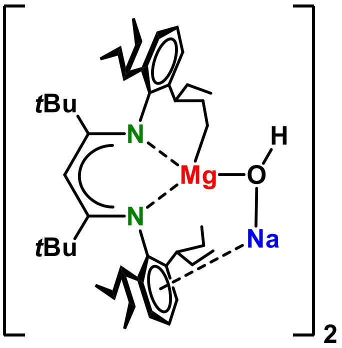


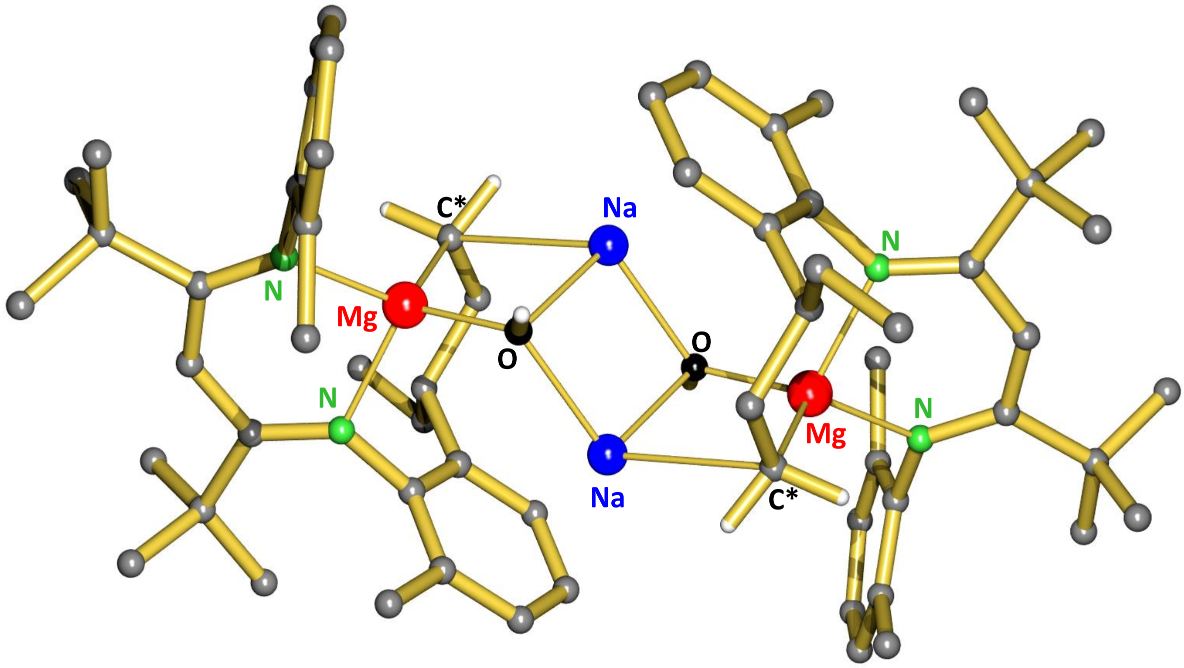


**Figure 1.** Crystal structure of complex **4**. Most H atoms and part of the Et-substituents are not shown for clarity.

**Decomposition of (BDI*)MgNa$_3$N''$_2$ (1) in the presence of THF**

To a solution of (BDI*)MgNa$_3$N''$_2$ (**1**) (31.1 mg, 30.3 $\mu$mol, 1.0 eq) in cyclohexane-d$_{12}$ (500 $\mu$L) THF (10 $\mu$L, 123 $\mu$mol, 4.0 eq) was added. Upon stirring, the dark-red solution immediately changed its color to yellow and a grey, metallic precipitate formed. The solid was isolated by filtration and washed with hexanes. The filtrate and the obtained solid were both dried *in vacuo*.

The dried filtrate was dissolved in $C_6D_6$ and $^1$H NMR spectroscopy (**Figure 45**) and single crystal X-ray diffraction (**Figure 68** & **Figure 69**) revealed the formation of (BDI*)Na(THF)$_x$ and (NaN'')$_3$ in a ratio of 1:0.66. The grey powdery solid did not dissolve in alkanes, aromatics or THF but reacted violently with aqueous diluted HCl under formation of sparkles and therefore likely consists of Mg$^0$ which according to the mass balance should be formed: **1** + x THF ⟶ (BDI*)Na(THF)$_x$ + 0.66 (NaN'')$_3$ + Mg$^0$.

**Decomposition of (BDI*)MgNa$_3$N''$_2$ (1) in the presence of benzene**

A solution of (BDI*)MgNa$_3$N''$_2$ (**1**) (31.1 mg, 30.3 $\mu$mol) in $C_6H_6$ (4 mL) was stirred for 7 days at room temperature. During this time, the red color of **1** changed to yellow, indicating the full consumption of **1**. A white precipitate and the formation of small amounts of a metal mirror at the glassware was observed after the reaction was finished.

The white precipitate was isolated by filtration. Upon addition of aqueous HCl to this compound, hydrogen gas was liberated. This led us to conclude that NaH and/or $MgH_2$ must have been formed during this reaction.

The metal mirror was quenched with $D_2O$ and $^{23}$Na NMR spectroscopy (**Figure 46**) gave a clear signal suggesting the presence of Na ions, while $^{25}$Mg NMR was silent. However, due to the low sensitivity of $^{25}$Mg NMR spectroscopy, the presence of $Mg^0$ within the metal mirror cannot be excluded.

The filtrate was evaporated to dryness and dissolved in $C_6D_6$. $^1$H NMR analysis identified, besides some minor BDI*-containing species, the following main products: (BDI*)MgPh (circa 50%), (BDI*)Na (circa 25%) and (NaN'')$_3$ (**Figure 47**).

**Deoxygenation of epoxides with (BDI*)MgNa$_3$N''$_2$ (1)**

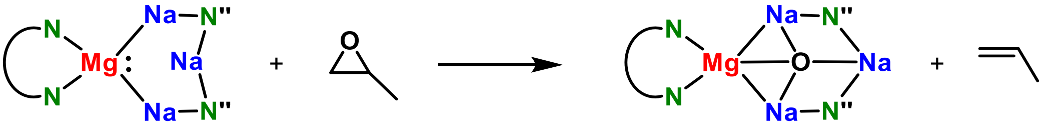


To a solution of (BDI*)MgNa$_3$N''$_2$ (**1**) (31.1 mg, 30.3 $\mu$mol, 1.0 eq) in cyclohexane-$d_{12}$ propylene oxide (2.1 $\mu$L, 30.3 $\mu$mol, 1.0 eq) was added. The solution immediately changed its color from dark-red to yellow. Based on $^1$H NMR spectroscopy, **1** and propylene oxide were quantitatively converted to (BDI*)MgNa$_3$N''$_2$O (**2**) and propylene (**Figure 50**).

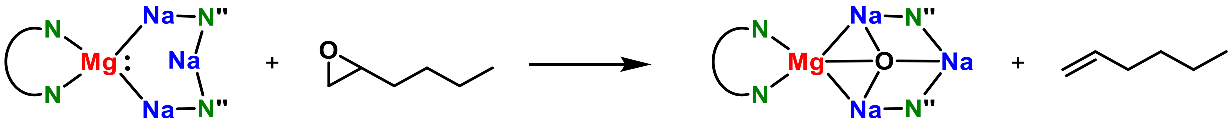


To a solution of (BDI*)MgNa$_3$N''$_2$ (**1**) (31.1 mg, 30.3 $\mu$mol, 1.0 eq) in cyclohexane-$d_{12}$ 1,2-epoxyhexane (3.7 $\mu$L, 30.3 $\mu$mol, 1.0 eq) was added. The solution immediately changed its color from dark-red to yellow. Based on $^1$H NMR spectroscopy, **1** and 1,2-epoxyhexane were quantitatively converted to (BDI*)MgNa$_3$N''$_2$O (**2**) and 1-hexene (**Figure 51**).

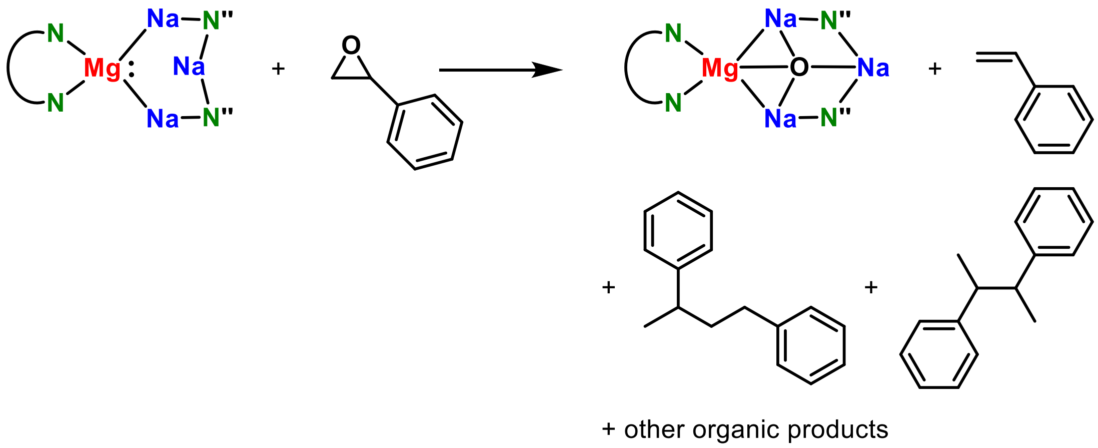


To a solution of (BDI*)MgNa$_3$N''$_2$ (**1**) (31.1 mg, 30.3 $\mu$mol, 1.0 eq) in cyclohexane-$d_{12}$ styrene oxide (3.5 $\mu$L, 30.3 $\mu$mol, 1.0 eq) was added. The dark-red solution immediately changed its color from dark-red to yellow. Based on $^1$H NMR spectroscopy, **1** was quantitatively converted into (BDI*)MgNa$_3$N''$_2$O (**2**). Due to the tendency of styrene to oligomerize, several organic products, including styrene itself could be identified by $^1$H NMR spectroscopy and GC-MS (**Figure 52** – **Figure 57**).

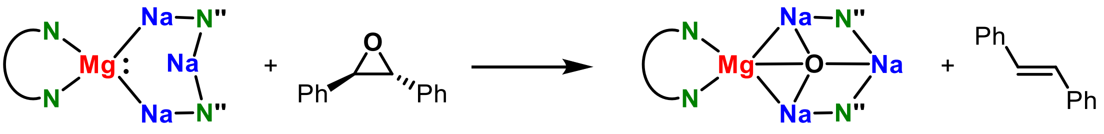


To a solution of (BDI*)MgNa$_3$N''$_2$ (**1**) (31.1 mg, 30.3 $\mu$mol, 1.0 eq) in cyclohexane-$d_{12}$ *trans*-stilbene oxide (6.0 mg, 30.6 $\mu$mol, 1.0 eq) was added. The solution immediately changed its color from dark-red to yellow. Based on $^1$H NMR spectroscopy, **1** and *trans*-stilbene oxide were quantitatively converted to (BDI*)MgNa$_3$N''$_2$O (**2**) and *trans*-stilbene (**Figure 58**).

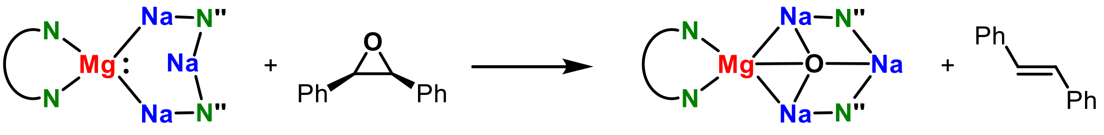


To a solution of (BDI*)MgNa$_3$N''$_2$ (**1**) (31.1 mg, 30.3 $\mu$mol, 1.0 eq) in cyclohexane-$d_{12}$ *cis*-stilbene oxide (6.0 mg, 30.6 $\mu$mol, 1.0 eq) was added. The solution immediately changed its color from dark-red to yellow. Based on $^1$H NMR spectroscopy, **1** and *cis*-stilbene oxide were quantitatively converted to (BDI*)MgNa$_3$N''$_2$O (**2**) and *trans*-stilbene (**Figure 59**).

## 2. Spectroscopic data

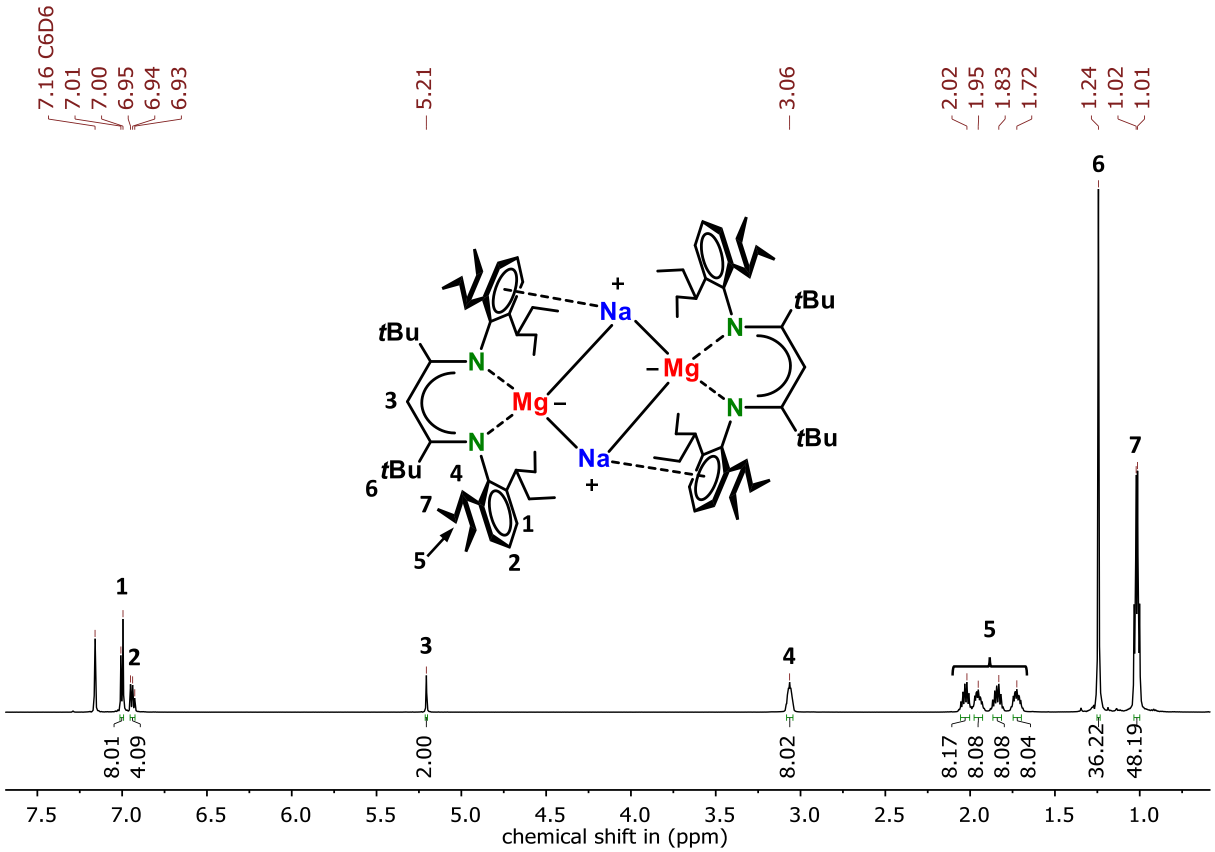


**Figure 2.** $^{1}$H NMR spectrum (600 MHz, 298 K, $C_6D_6$) of [(BDI*)MgNa]$_2$ (**I**).

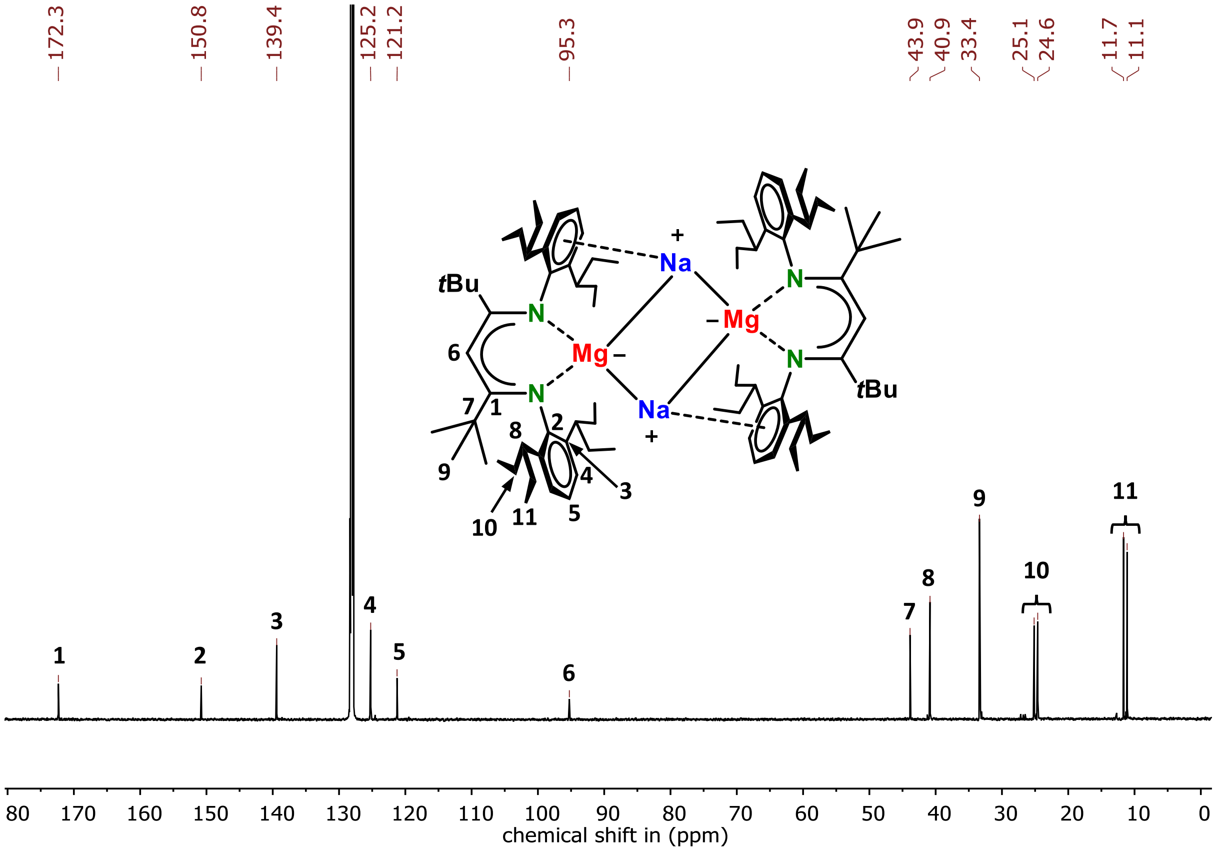


**Figure 3.** $^{13}C\{^1H\}$ NMR spectrum (151MHz, 298 K, $C_6D_6$) of [(BDI*)MgNa]$_2$ (**I**).

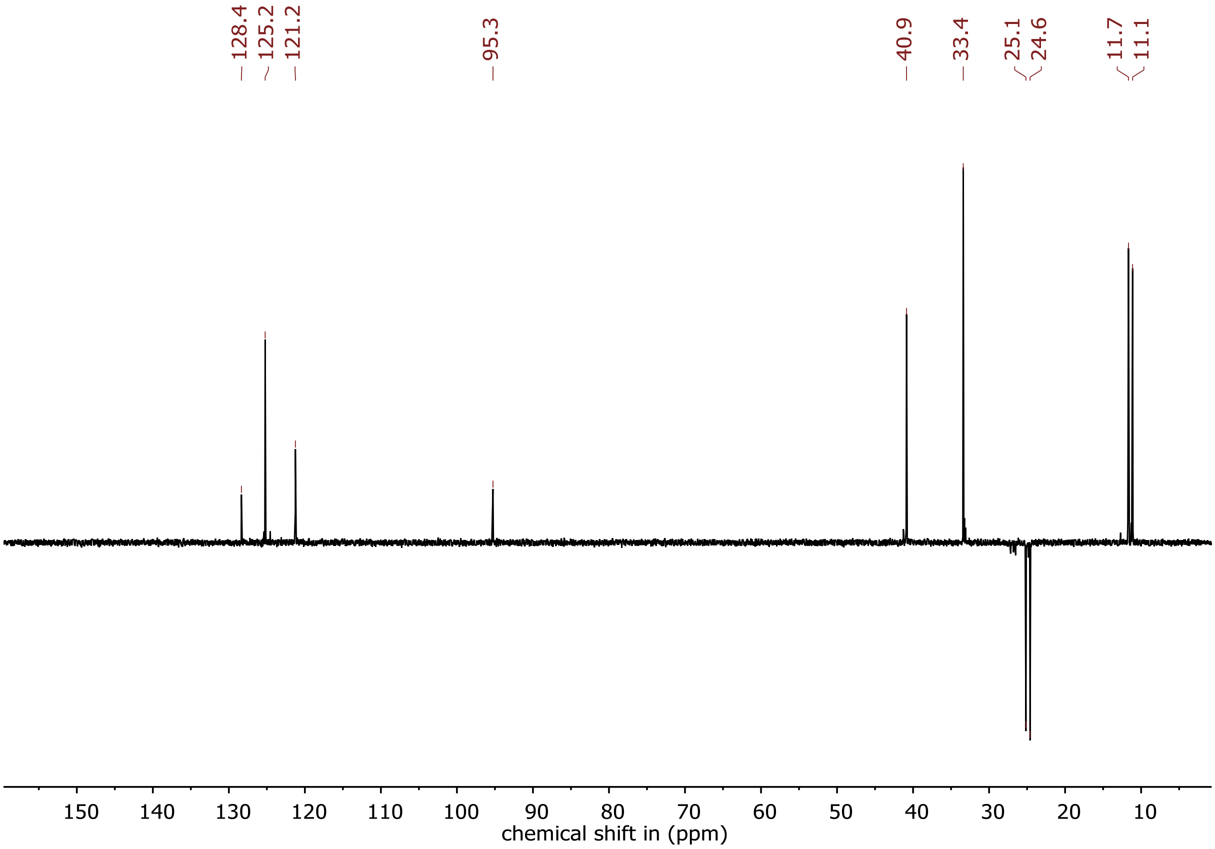


**Figure 4.** $^{13}C$ (DEPT 135) NMR spectrum (151 MHz, 298 K, $C_6D_6$) of [(BDI*)MgNa]$_2$ (**I**).

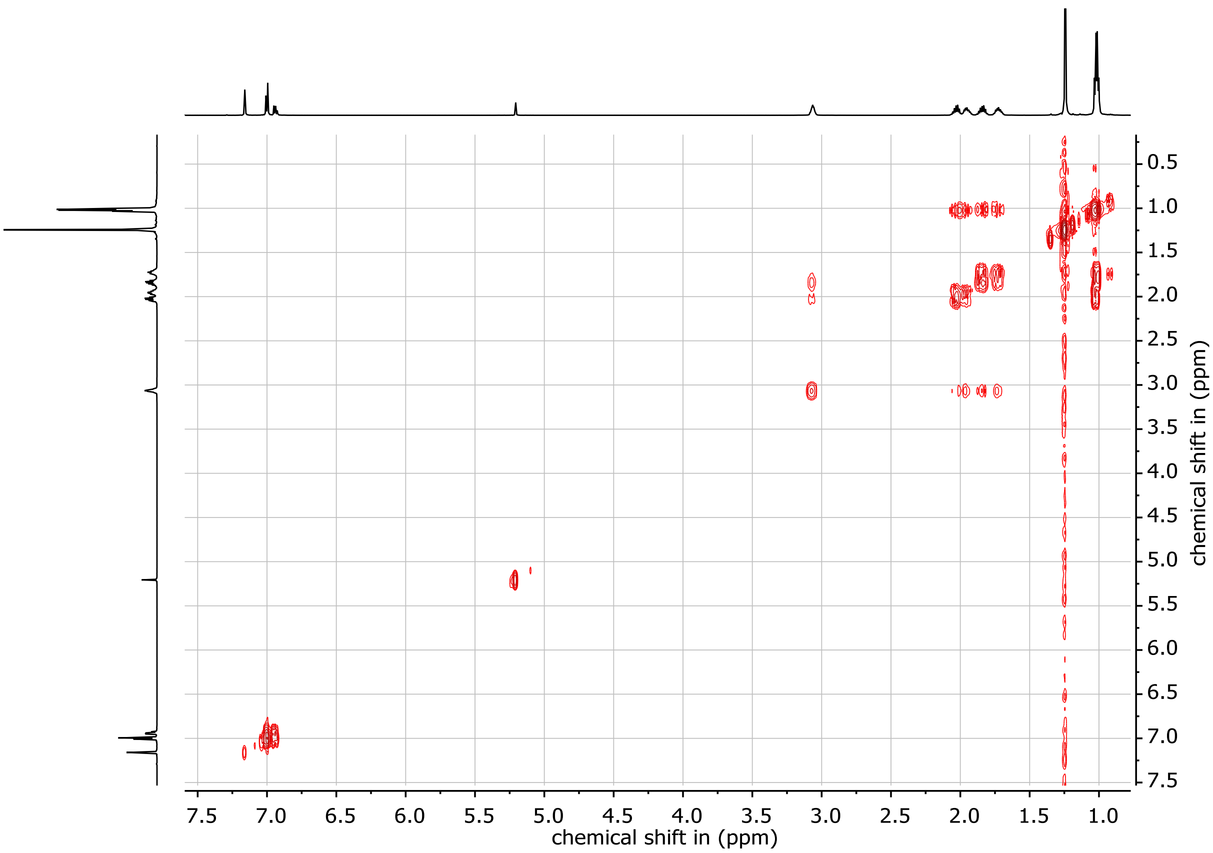


**Figure 5.** 2D-COSY NMR spectrum (600 MHz, 298 K, $C_6D_6$) of [(BDI*)MgNa]$_2$ (**I**).

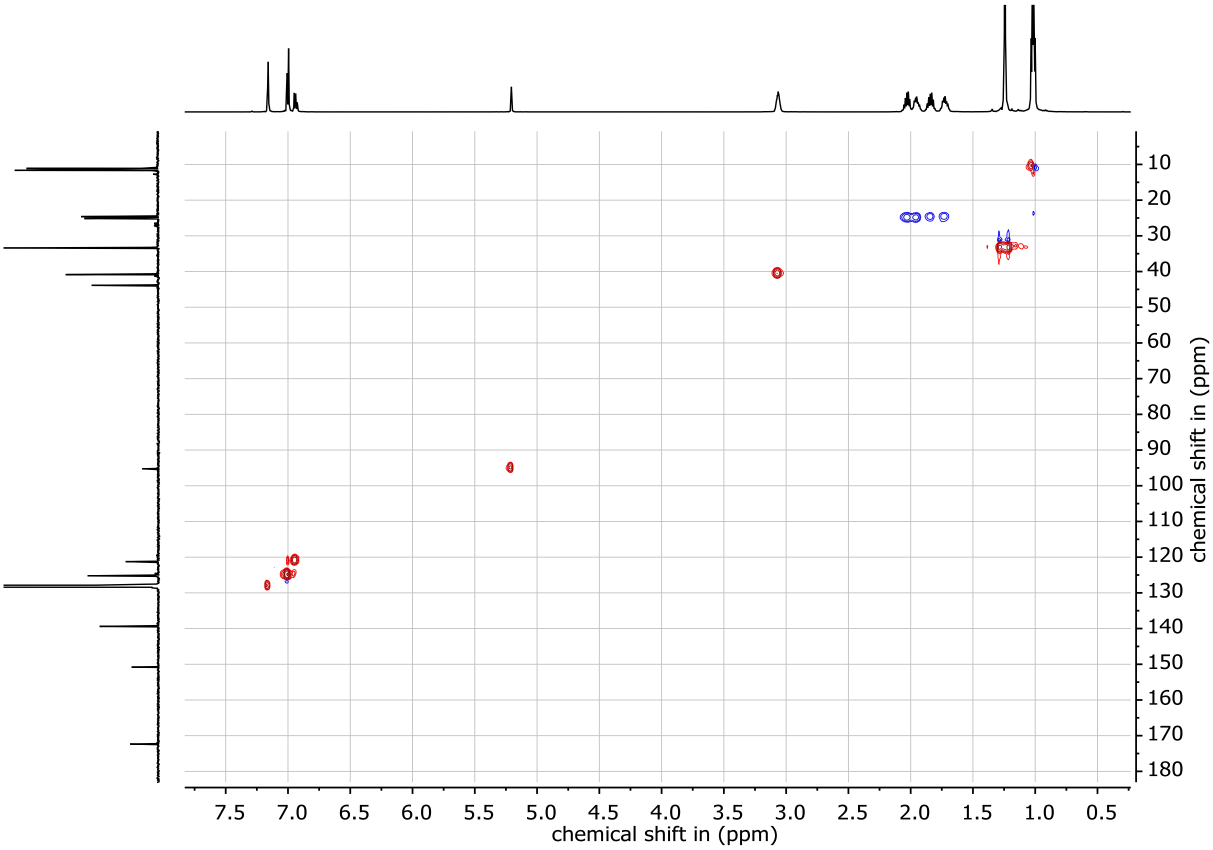


**Figure 6.** 2D-HSQC NMR spectrum (600/151 MHz, 298 K, $C_6D_6$) of [(BDI*)MgNa]$_2$ (**I**).

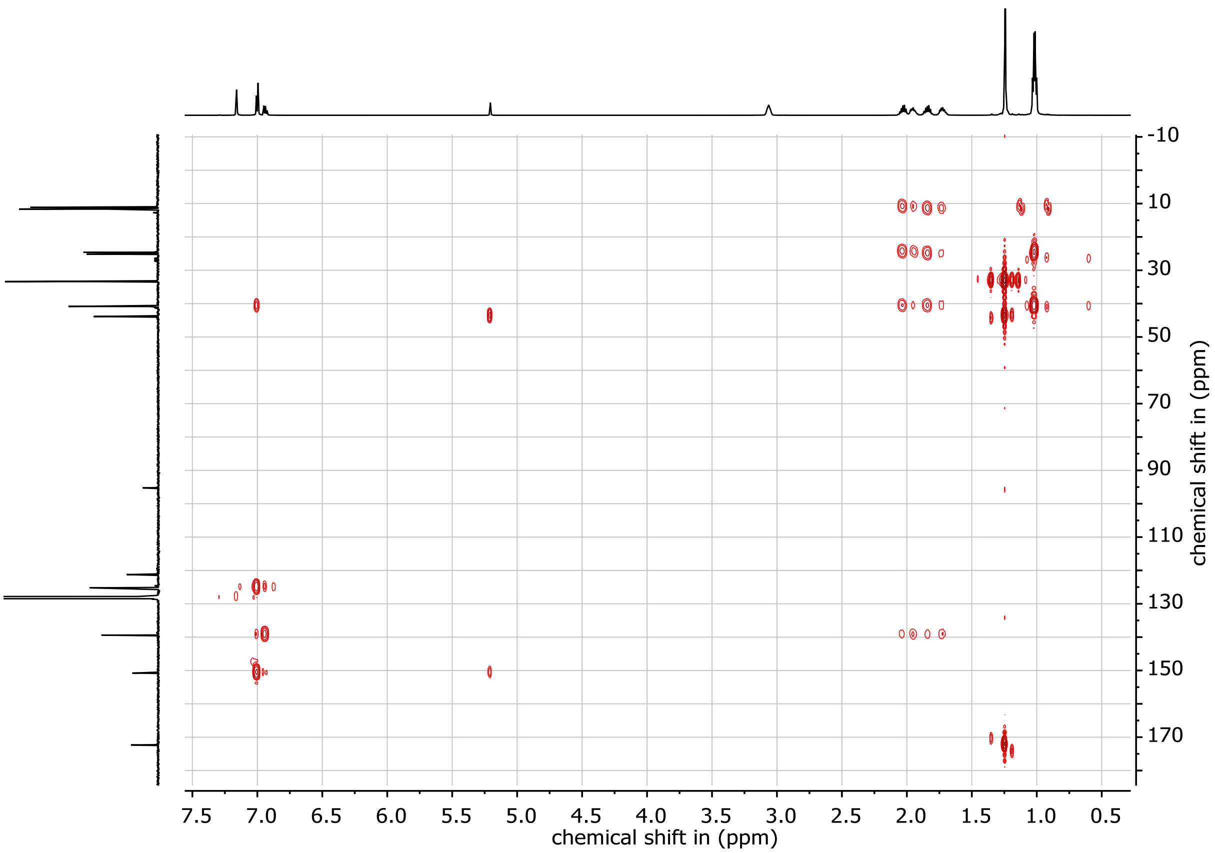


**Figure 7.** 2D-HMBC NMR spectrum (600/151 MHz, 298 K, $C_6D_6$) of [(BDI*)MgNa]$_2$ (**I**).

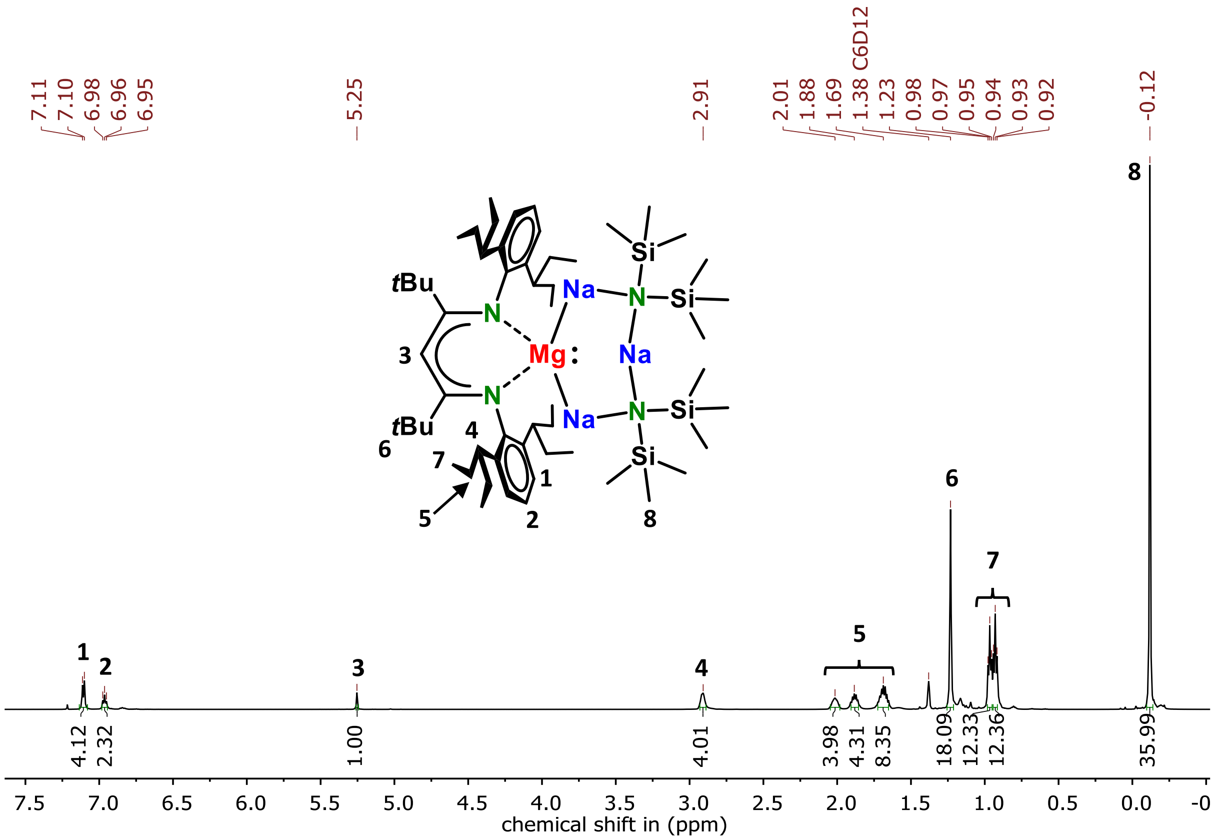


**Figure 8.** $^{1}$H NMR spectrum (600 MHz, 298 K, $C_6D_{12}$) of (BDI*)$MgNa_3N''_2$ (**1**).

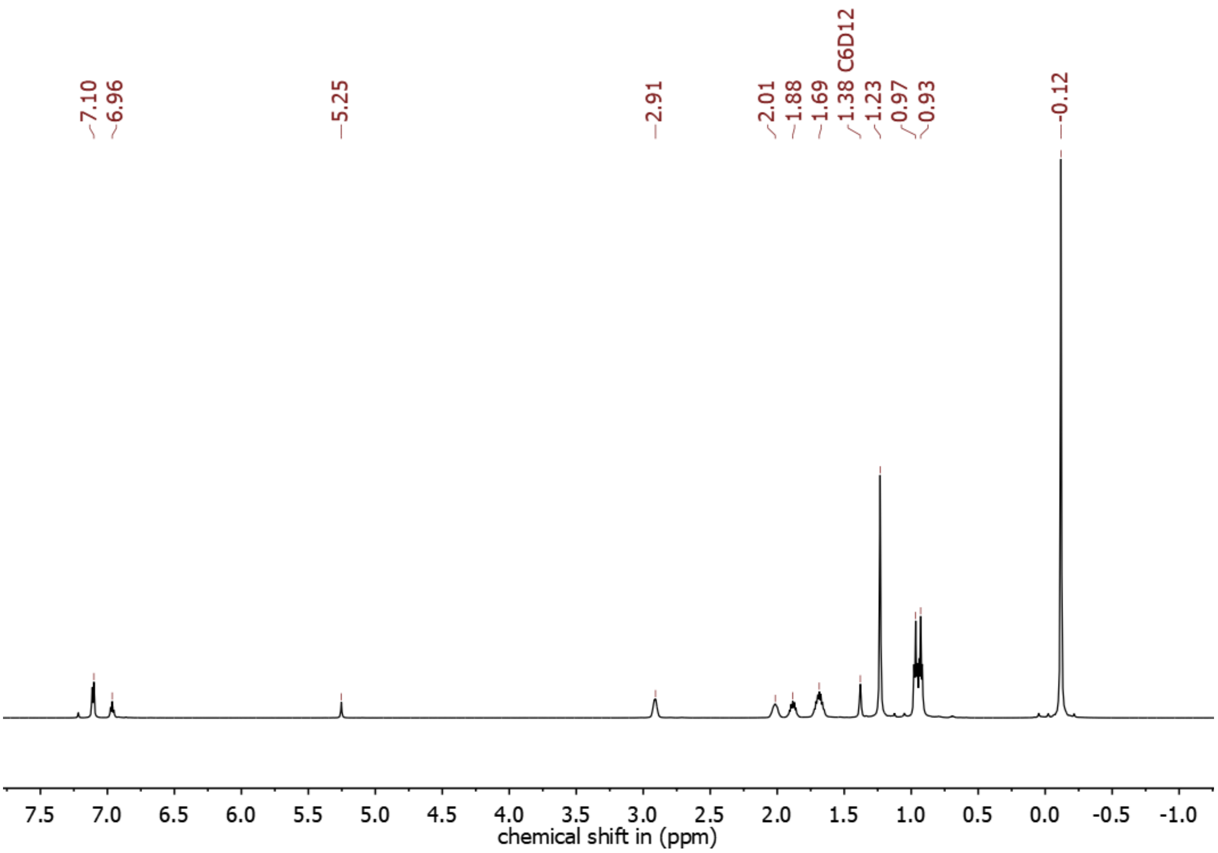


**Figure 9.** $^{1}H$ NMR spectrum (600 MHz, 298 K, $C_6D_{12}$) of crude (BDI*)$MgNa_3N''_2$ (**1**).

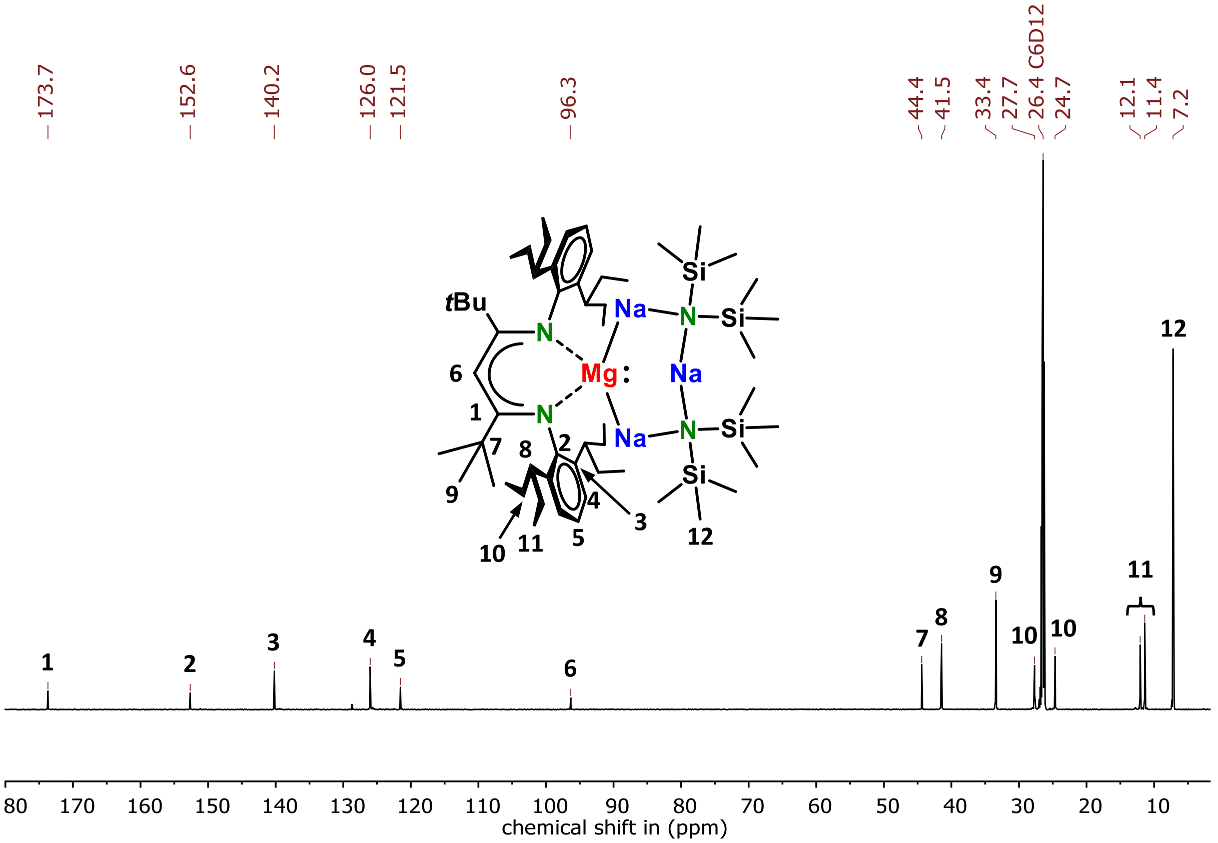


**Figure 10.** $^{13}C\{^{1}H\}$ NMR spectrum (150 MHz, 298 K, $C_6D_{12}$) of (BDI*)$MgNa_3N''_2$ (**1**).

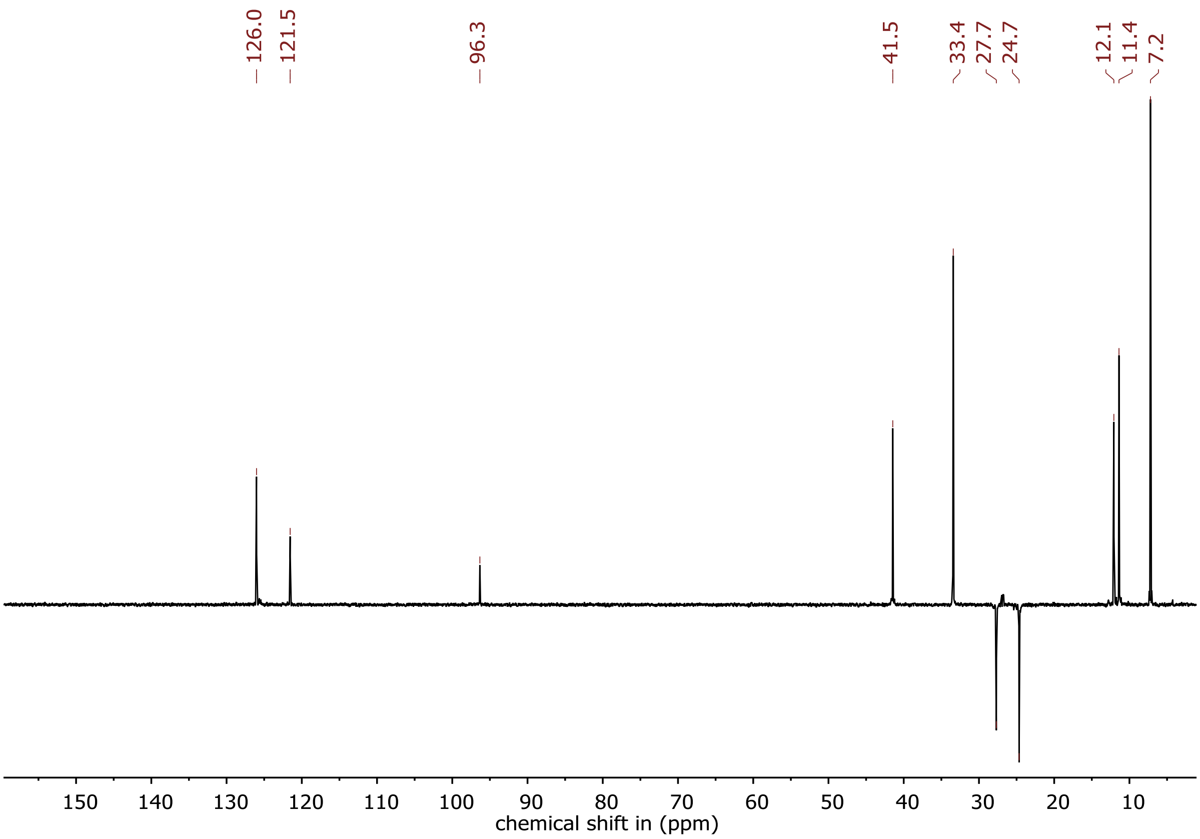


**Figure 11.** $^{13}C$ (DEPT 135) NMR spectrum (151 MHz, 298 K, $C_6D_{12}$) of (BDI*)$MgNa_3N''_2$ (**1**).

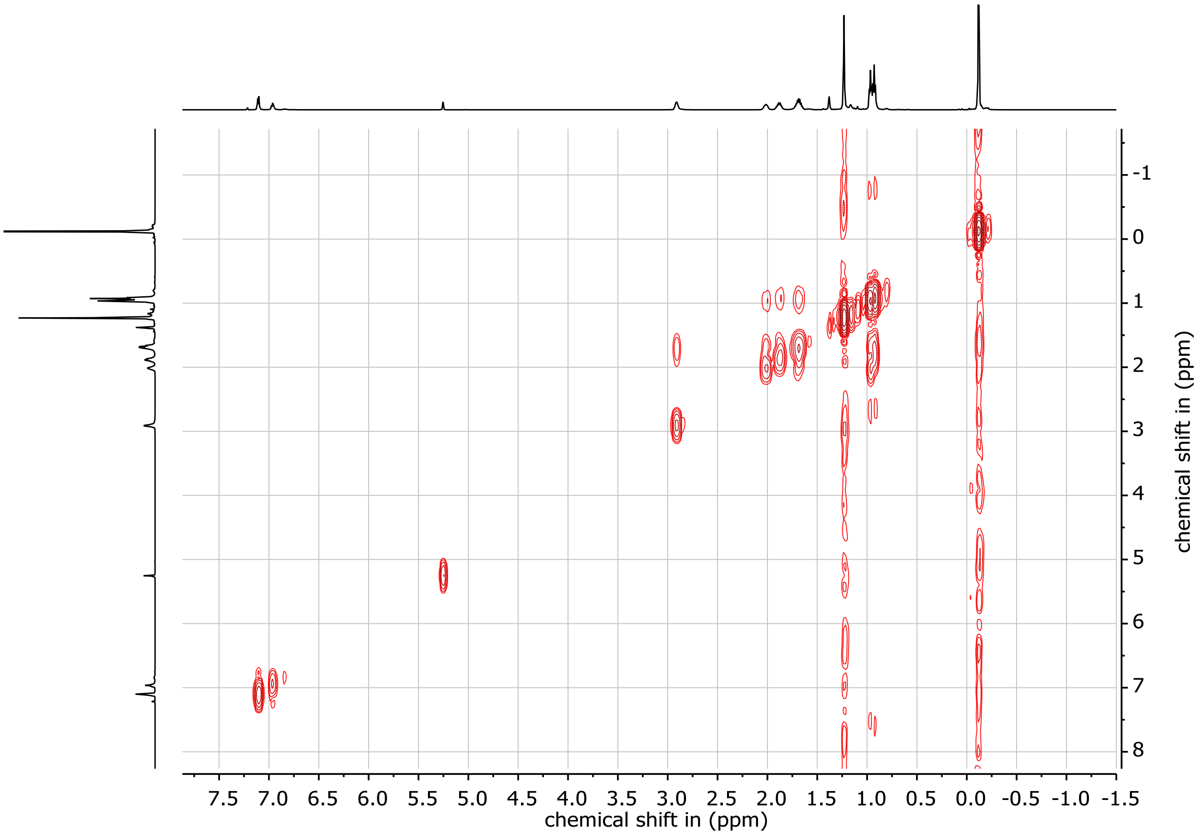


**Figure 12.** 2D-COSY NMR spectrum (600 MHz, 298 K, $C_6D_{12}$) of (BDI*)$MgNa_3N''_2$ (**1**).

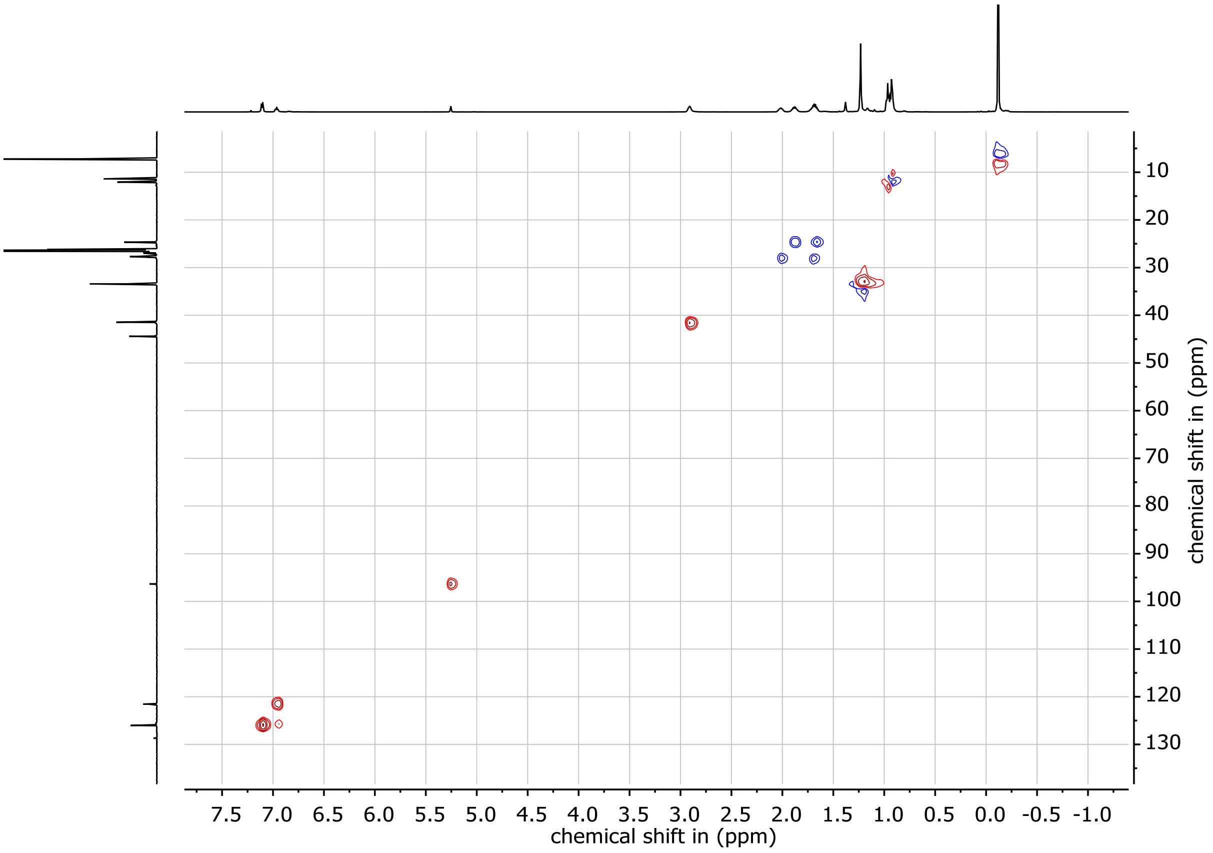


**Figure 13.** 2D-HSQC NMR spectrum (600/151 MHz, 298 K, $C_6D_{12}$) of (BDI*)$MgNa_3N''_2$ (**1**).

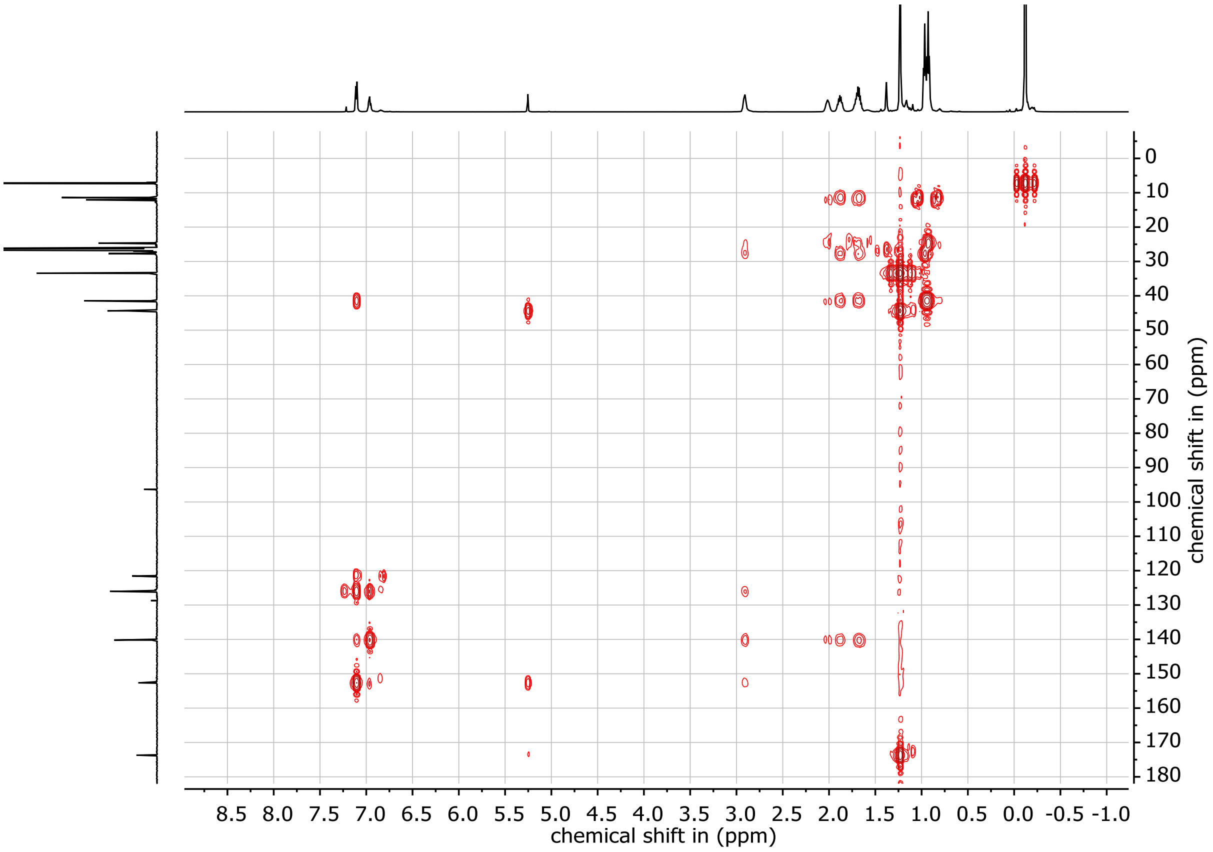


**Figure 14.** 2D-HMBC NMR spectrum (600/151 MHz, 298 K, $C_6D_{12}$) of (BDI*)$MgNa_3N''_2$ (**1**).

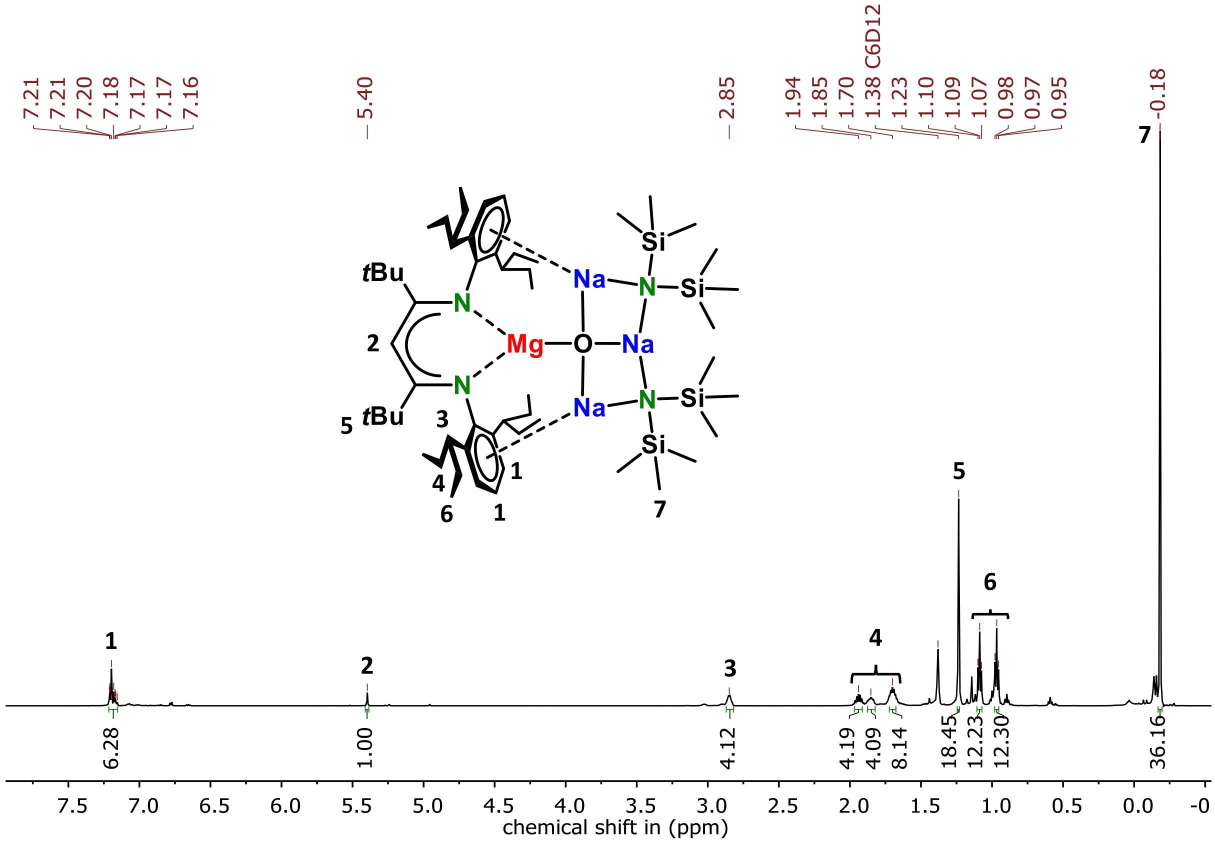


**Figure 15.** $^1$H NMR spectrum (600 MHz, 298 K, $C_6D_{12}$) of (BDI*)$MgNa_3N''_2O$ (**2**).

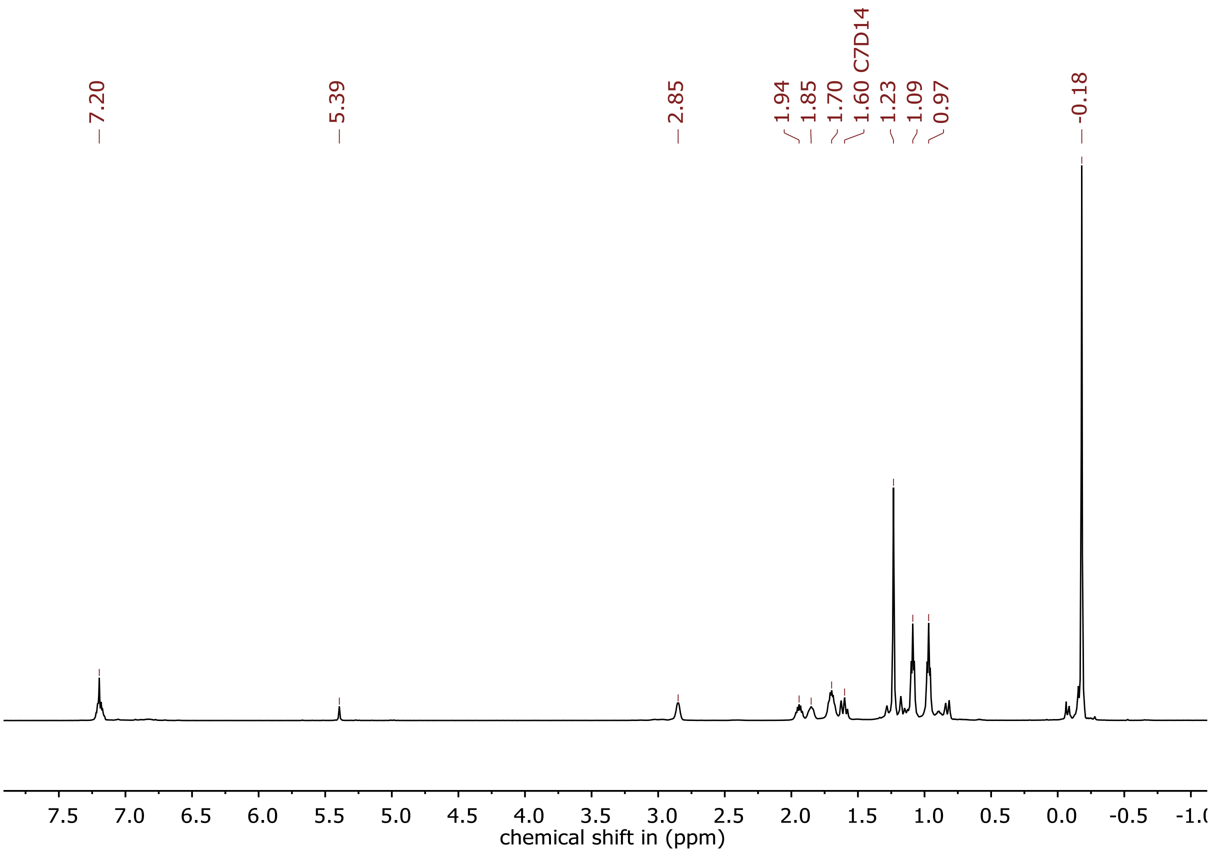


**Figure 16.** $^{1}H$ NMR spectrum (600 MHz, 298 K, $C_7D_{14}$) of crude (BDI*)$MgNa_3N''_2O$ (**2**).

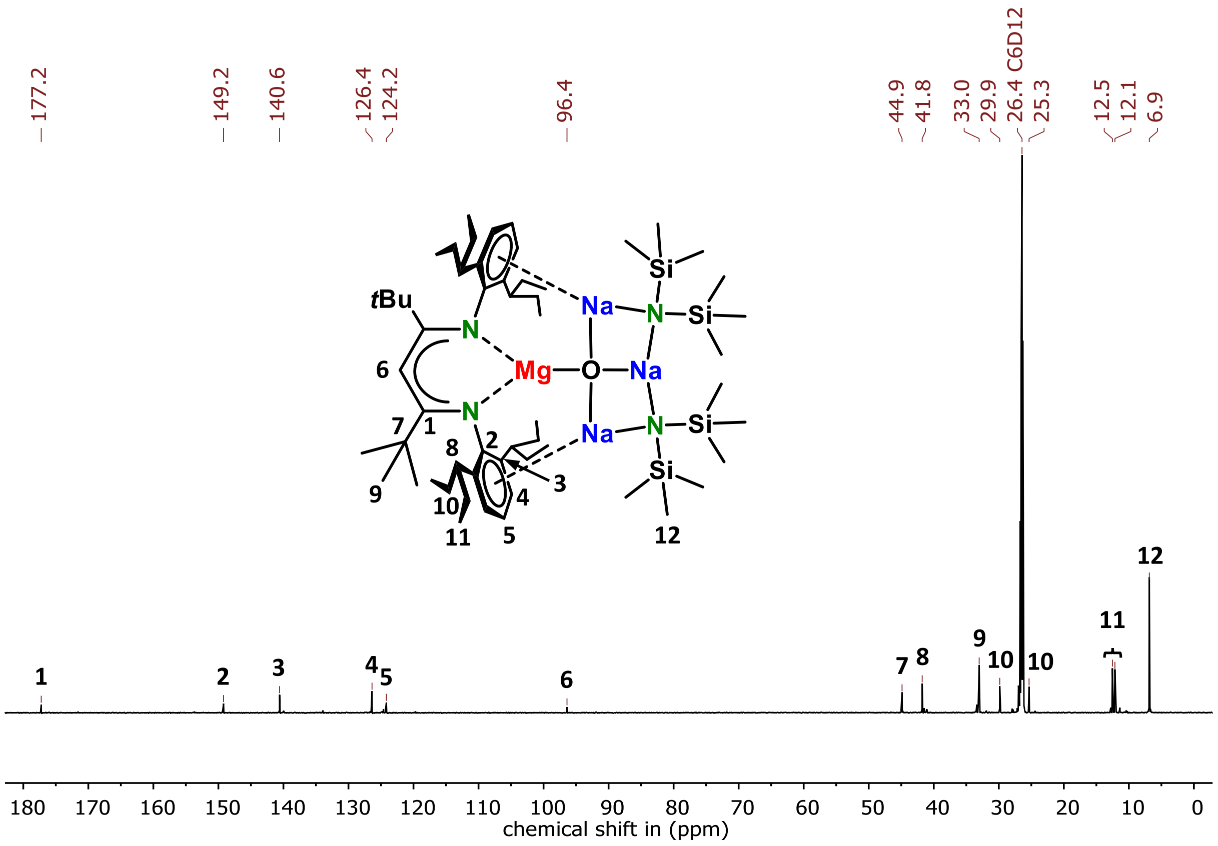


**Figure 17.** $^{13}C\{^{1}H\}$ NMR spectrum (150 MHz, 298 K, $C_6D_{12}$) of (BDI*)$MgNa_3N''_2O$ (**2**).

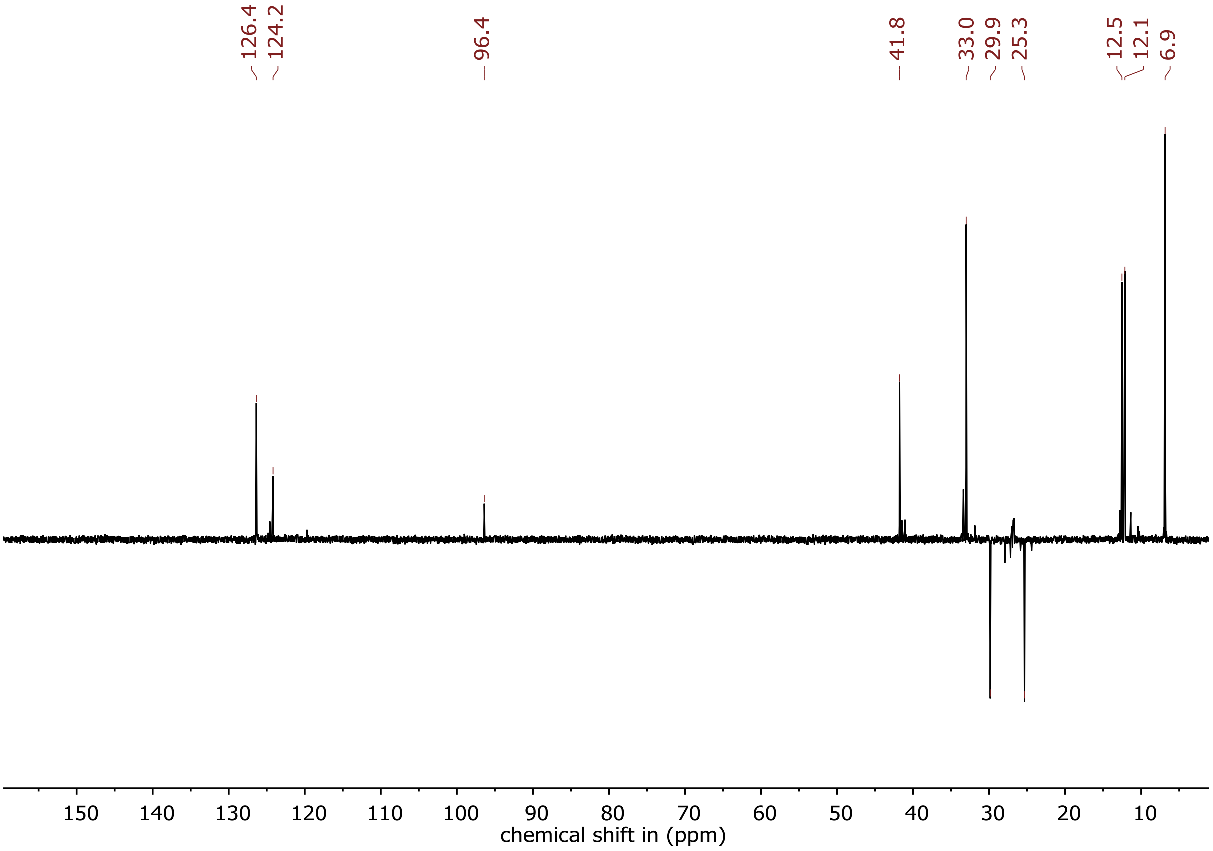


**Figure 18.** $^{13}C$ (DEPT 135) NMR spectrum (151 MHz, 298 K, $C_6D_{12}$) of (BDI*)MgNa$_3$N''$_2$O (**2**).

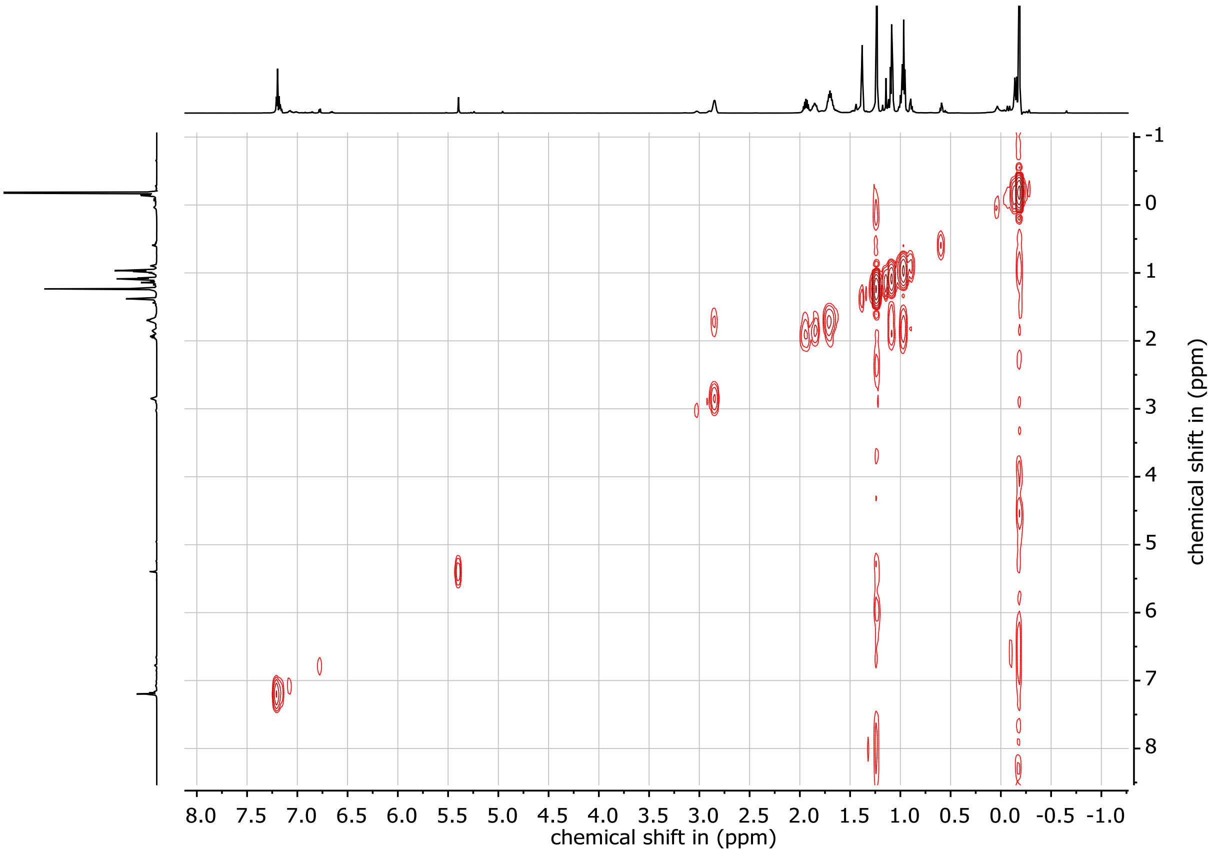


**Figure 19.** 2D-COSY NMR spectrum (600 MHz, 298 K, $C_6D_{12}$) of (BDI*)MgNa$_3$N''$_2$O (**2**).

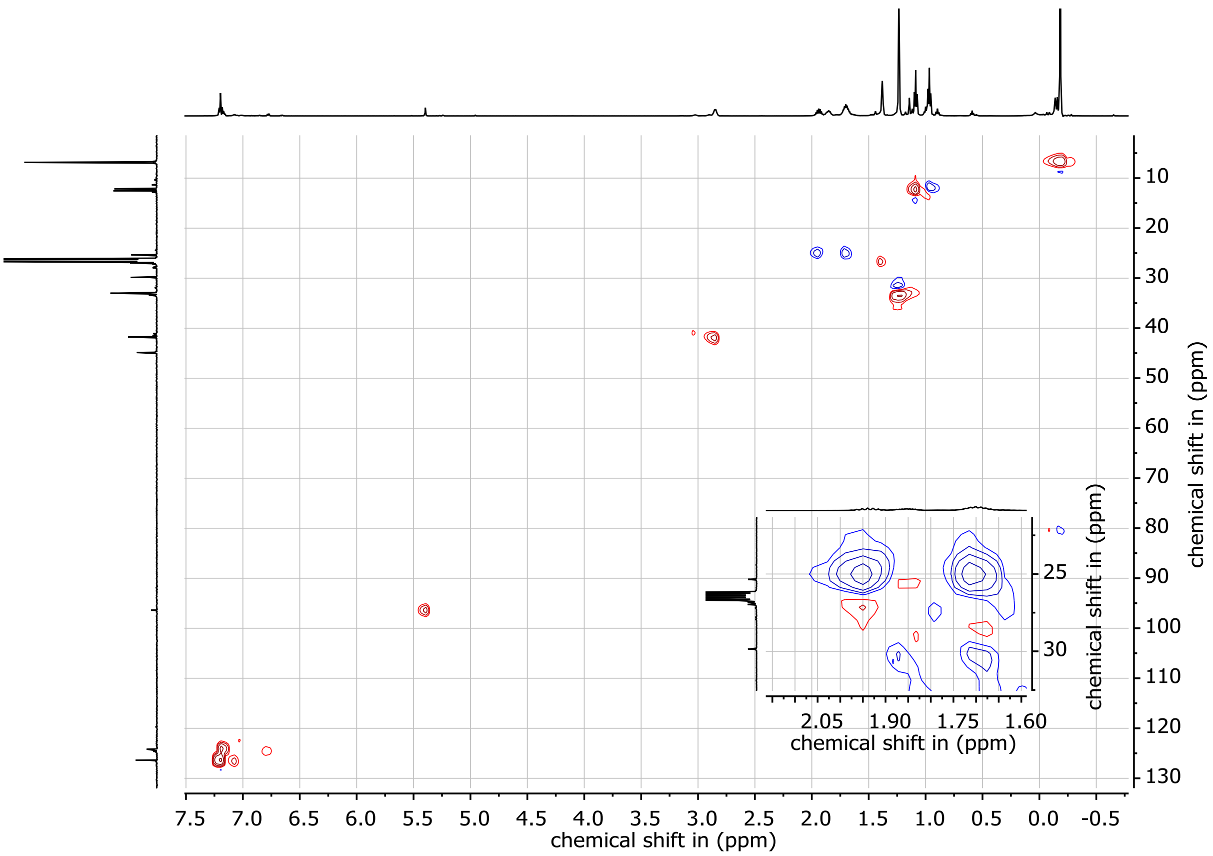


**Figure 20.** 2D-HSQC NMR spectrum (600/151 MHz, 298 K, $C_6D_{12}$) of (BDI*)MgNa$_3$N''$_2$O (**2**).

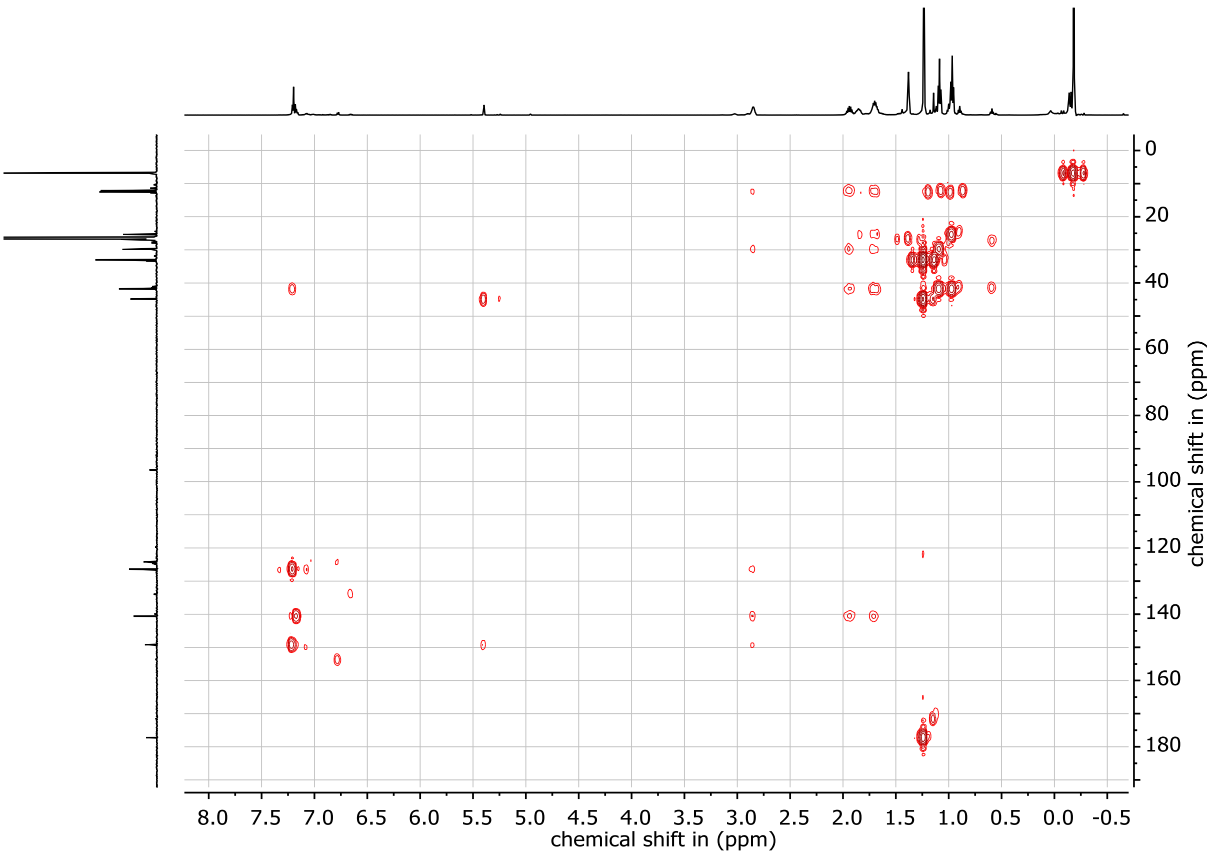


**Figure 21.** 2D-HMBC NMR spectrum (600/151 MHz, 298 K, $C_6D_{12}$) of (BDI*)MgNa$_3$N''$_2$O (**2**).

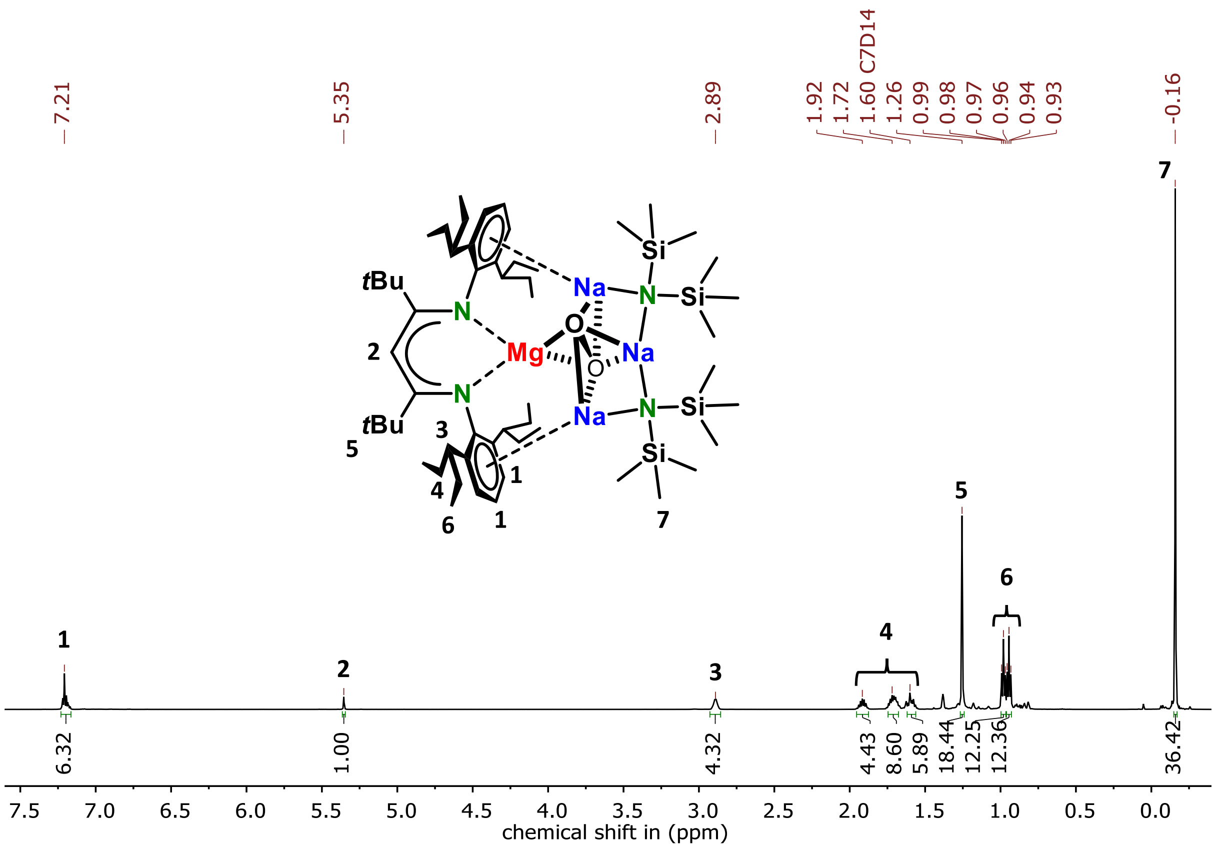


**Figure 22.** $^{1}$H NMR spectrum (600 MHz, 298 K, $C_7D_{14}$) of (BDI*)$MgNa_3N''_2(O_2)$ (**5**).

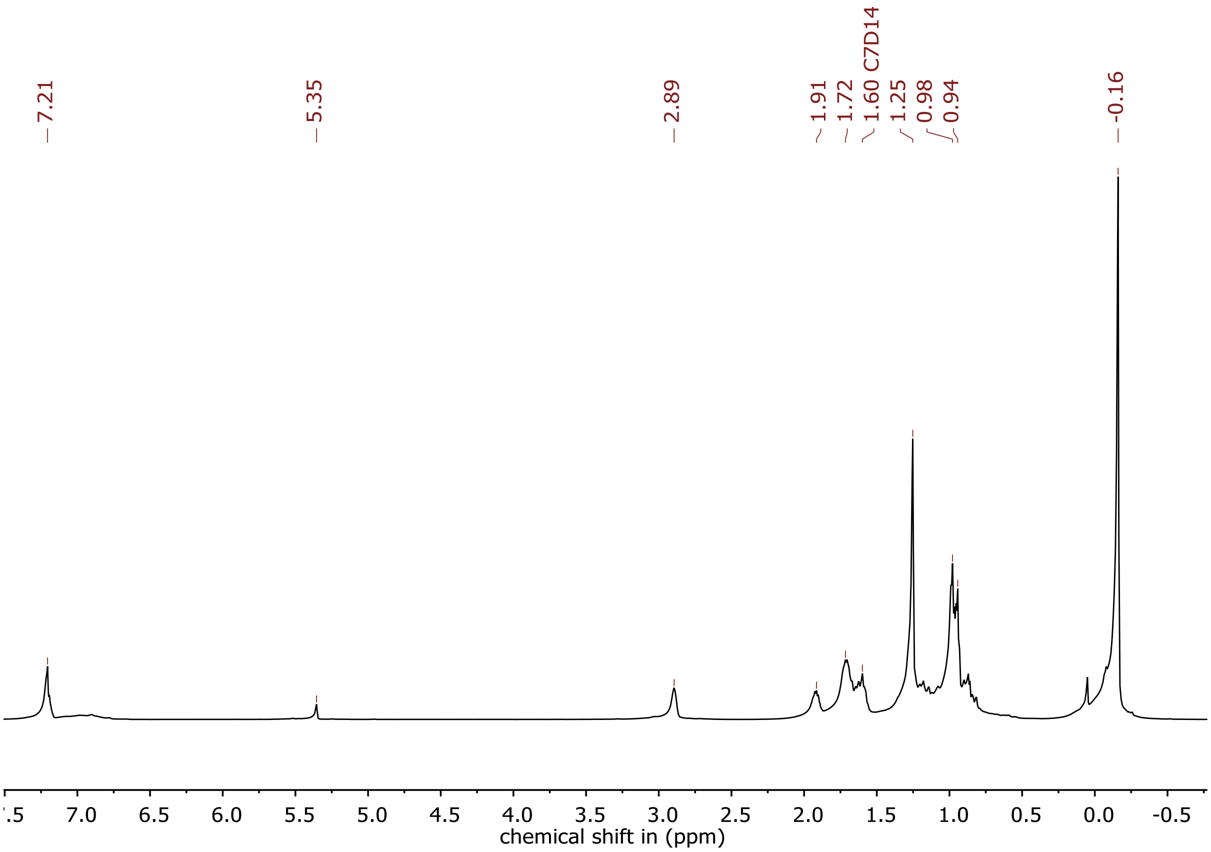


**Figure 23.** $^{1}$H NMR spectrum (600 MHz, 298 K, $C_7D_{14}$) of crude (BDI*)$MgNa_3N''_2(O_2)$ (**5**).

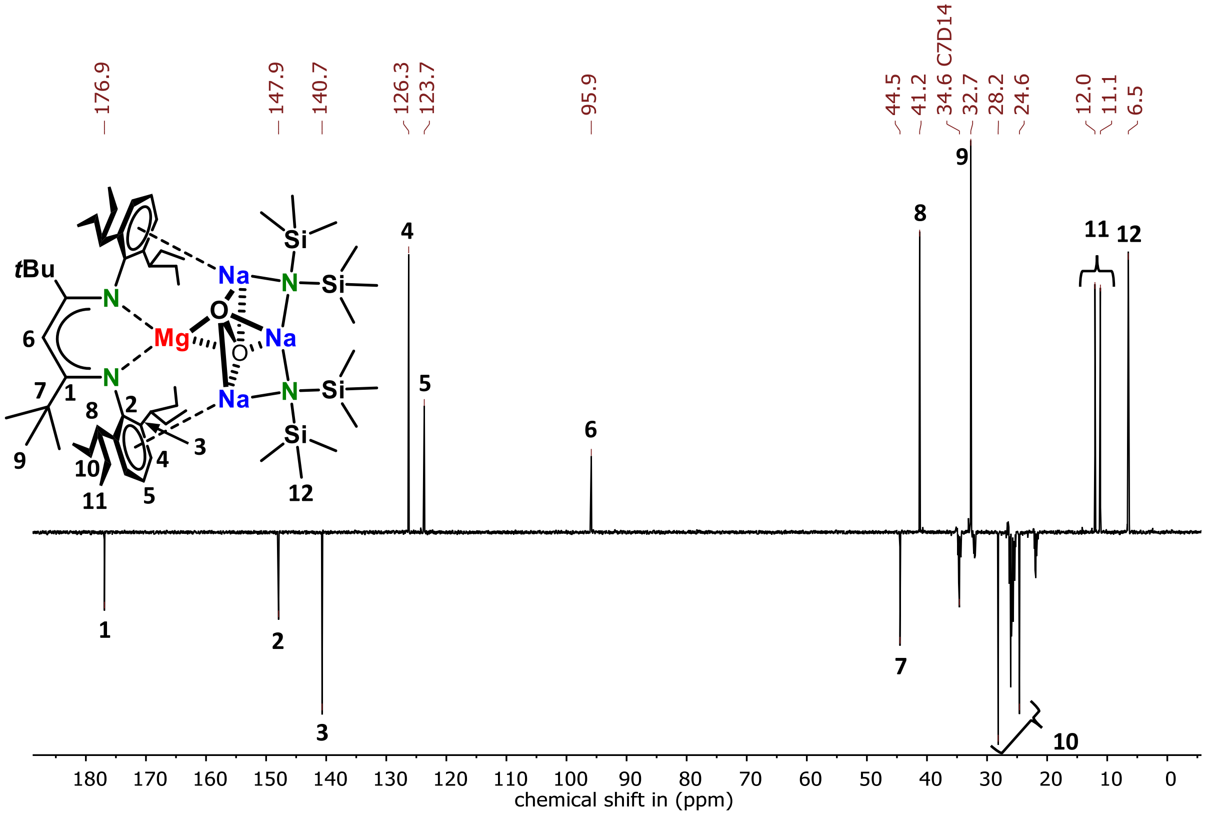


**Figure 24.** $^{13}$C-APT NMR spectrum (150 MHz, 298 K, $C_7D_{14}$) of (BDI*)MgNa$_3$N''$_2$(O$_2$) (**5**).

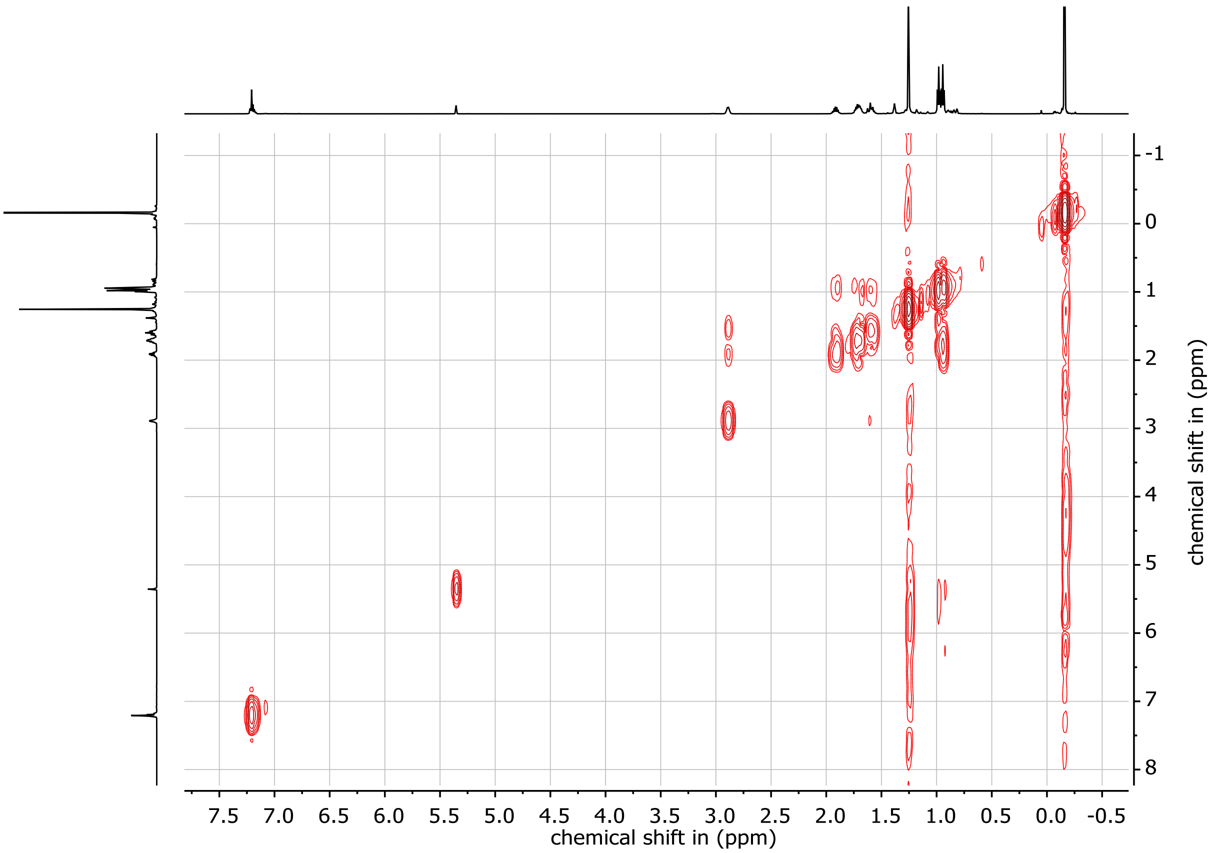


**Figure 25.** 2D-COSY NMR spectrum (600 MHz, 298 K, $C_7D_{14}$) of (BDI*)MgNa$_3$N''$_2$(O$_2$) (**5**).

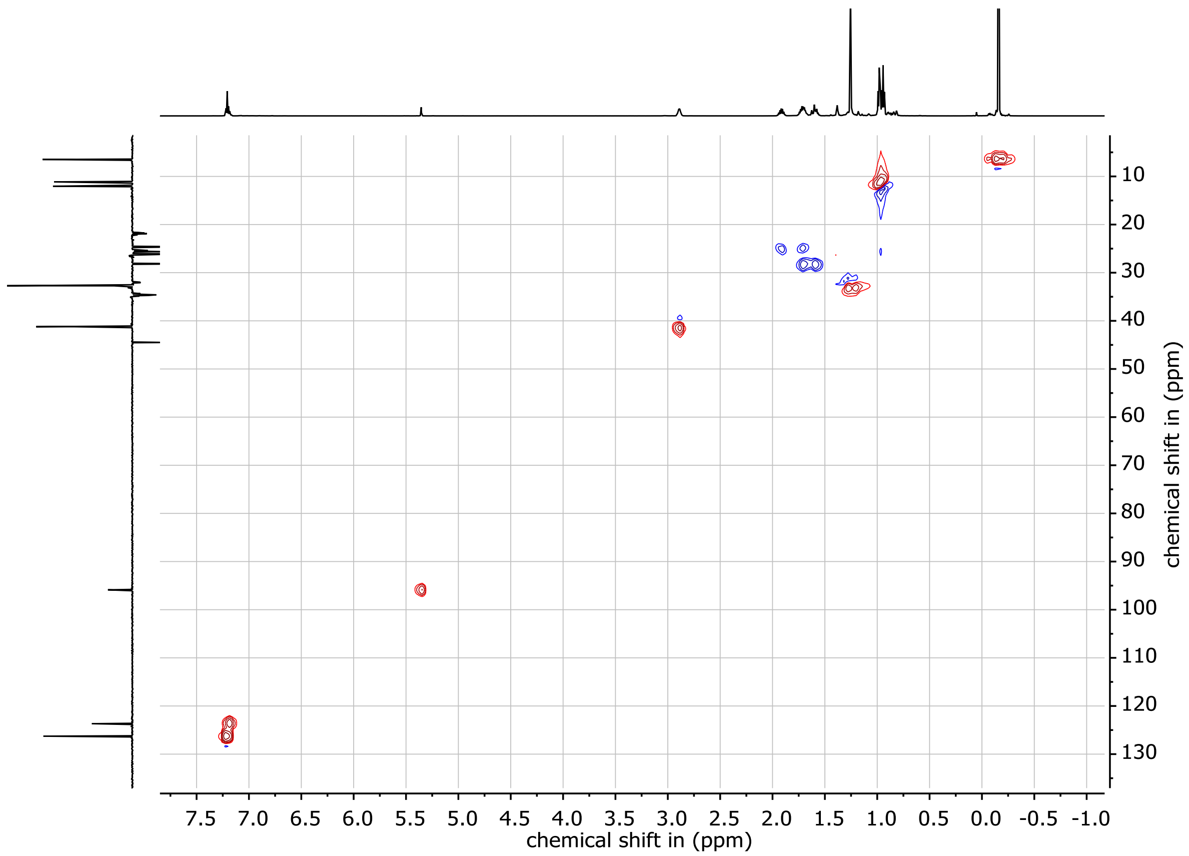


**Figure 26.** 2D-HSQC NMR spectrum (600/151 MHz, 298 K, $C_7D_{14}$) of (BDI*)MgNa$_3$N''$_2$(O$_2$) (**5**).

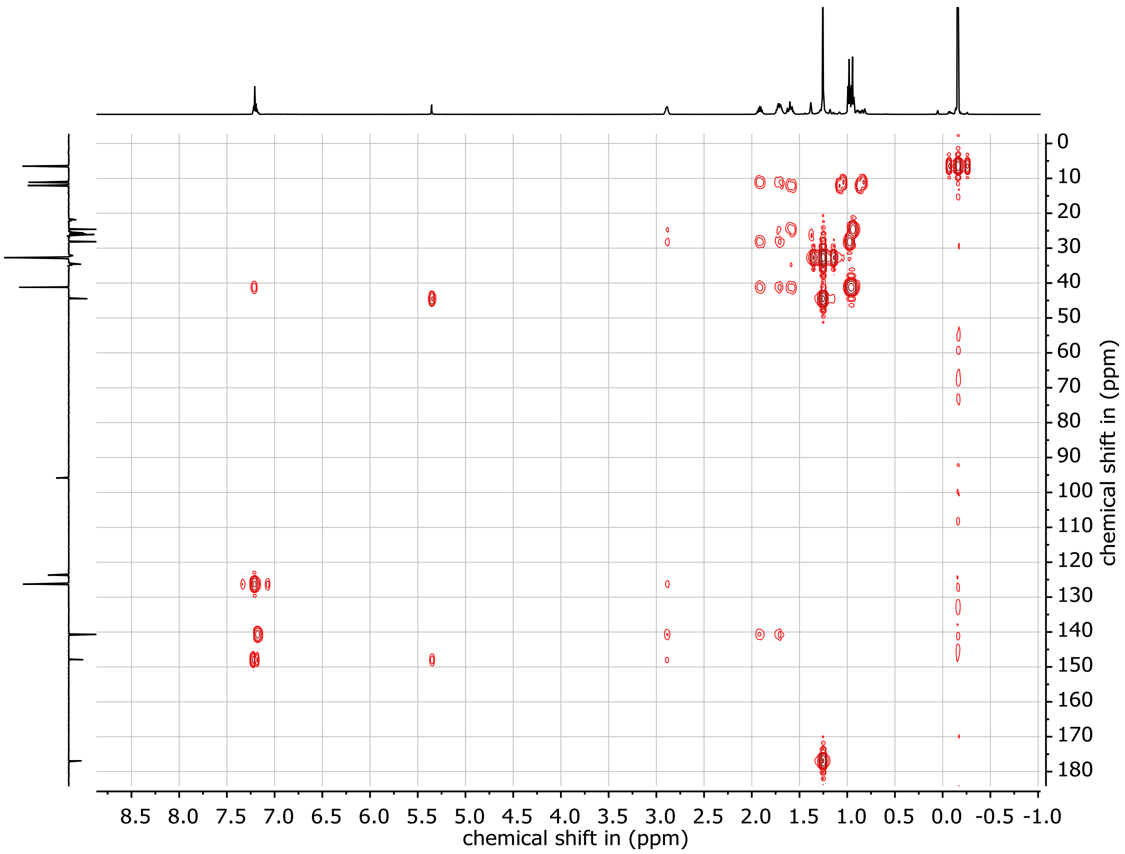


**Figure 27.** 2D-HMBC NMR spectrum (600/151 MHz, 298 K, $C_7D_{14}$) of (BDI*)MgNa$_3$N''$_2$(O$_2$) (**5**).

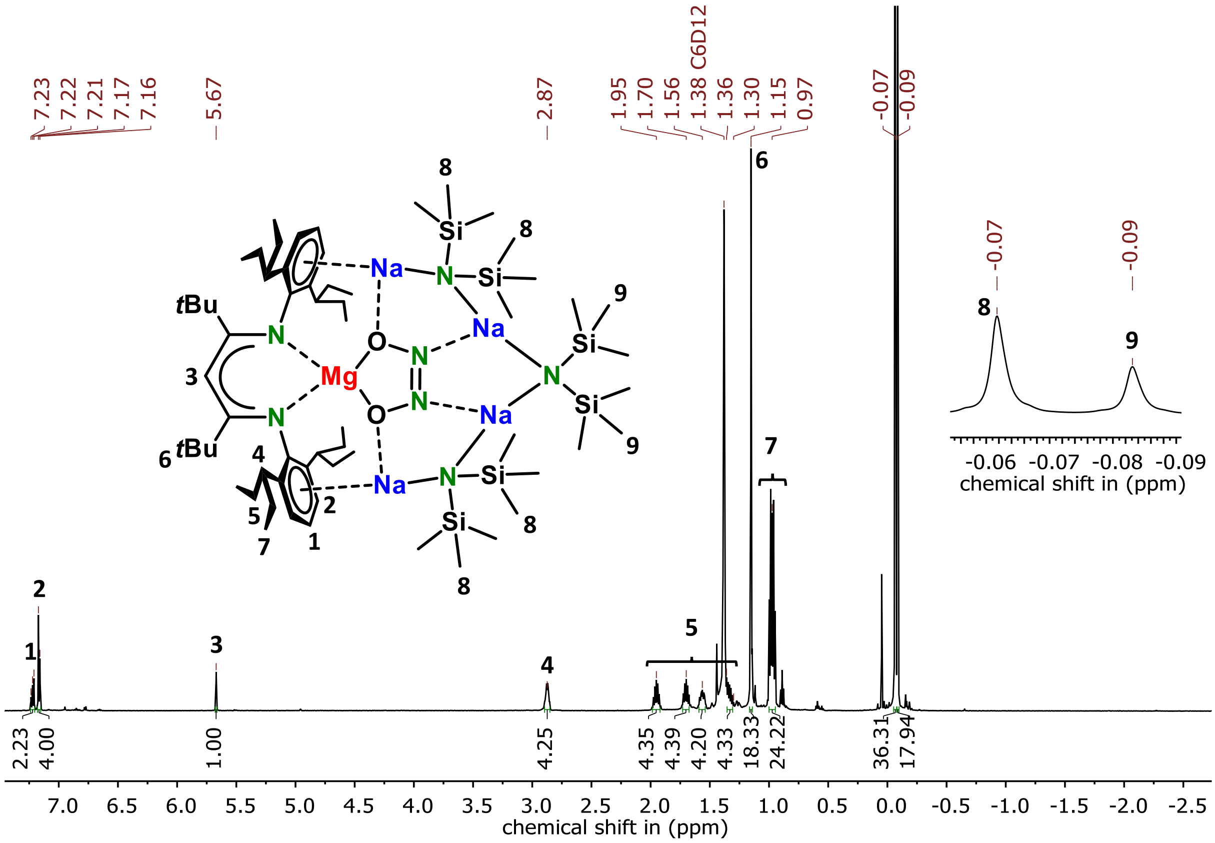

**Figure 28.** $^{1}H$ NMR spectrum (600 MHz, 298 K, $C_6D_{12}$) of (BDI*)$MgNa_4N''_3(N_2O_2)$ (**6**).

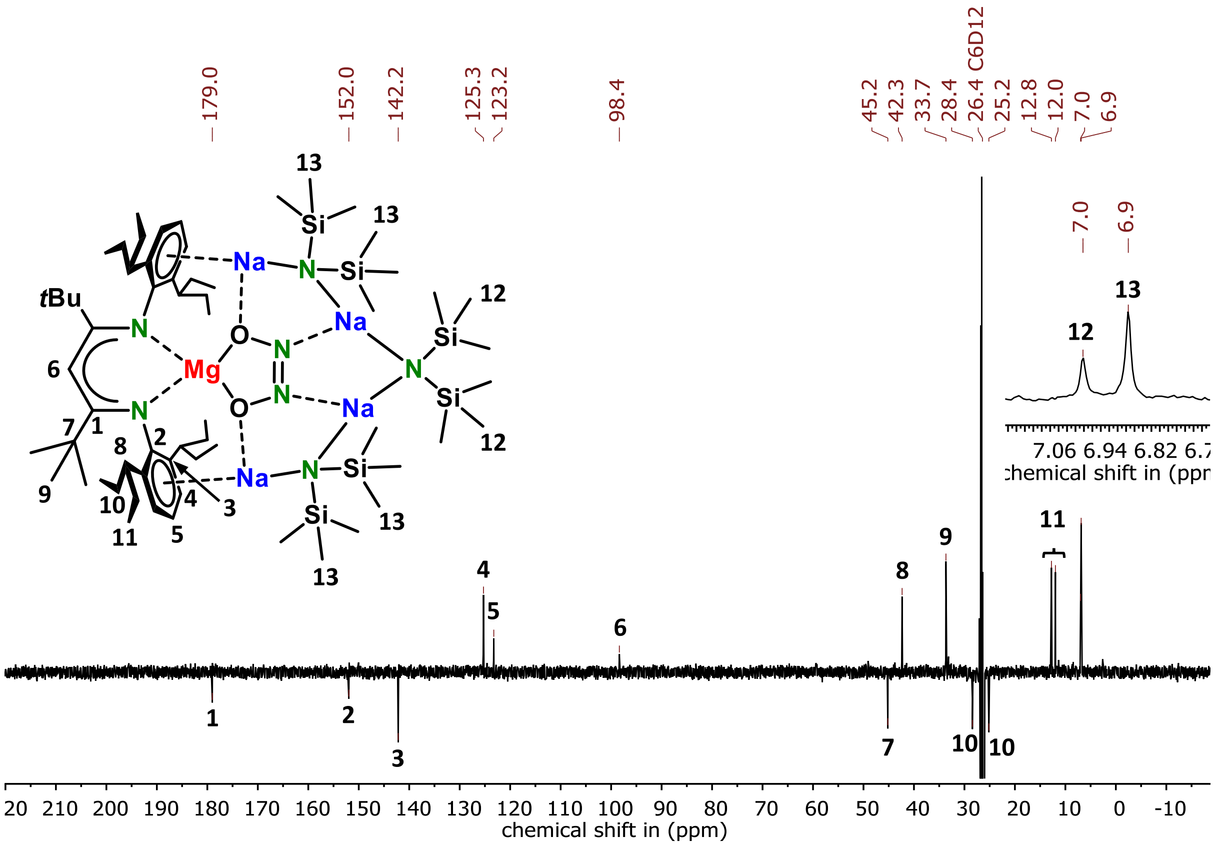

**Figure 29.** $^{13}C$-APT NMR spectrum (150 MHz, 298 K, $C_6D_{12}$) of (BDI*)$MgNa_4N''_3(N_2O_2)$ (**6**).

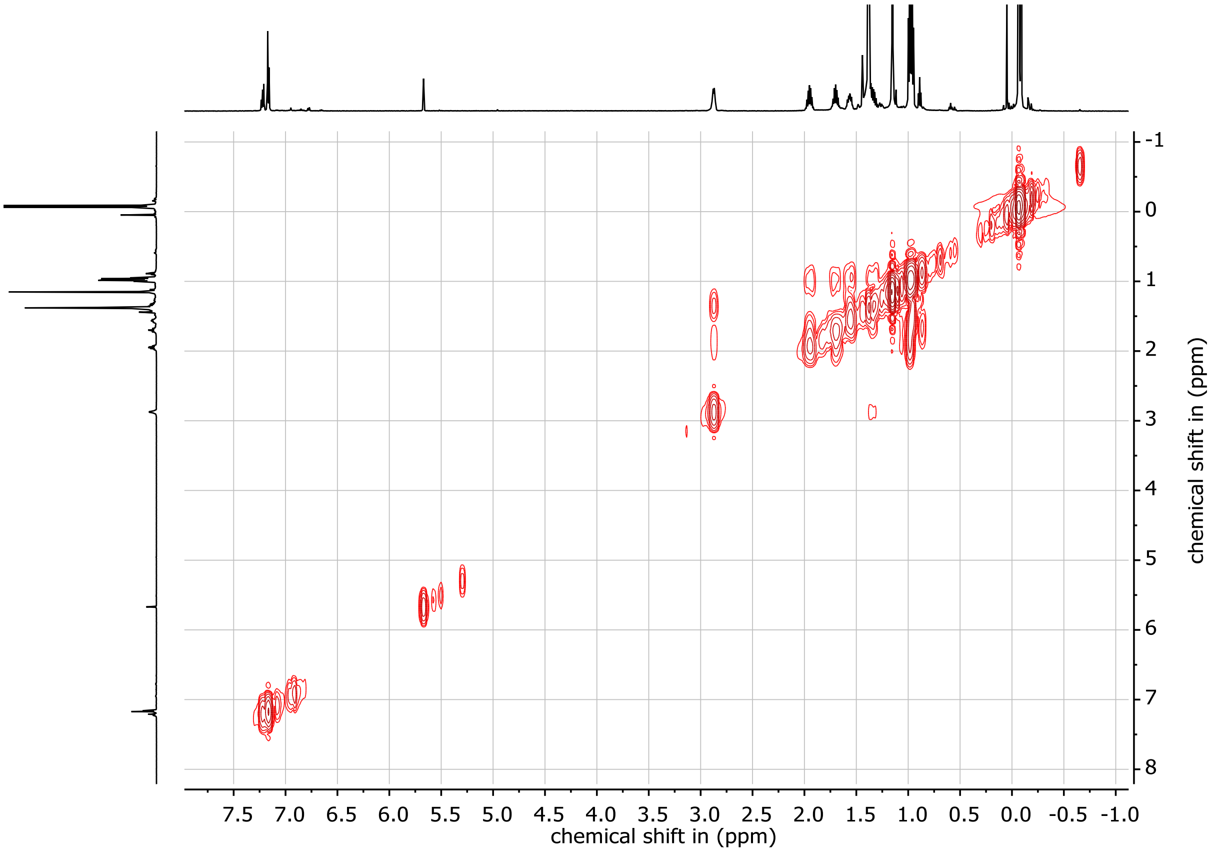


**Figure 30.** 2D-COSY NMR spectrum (600 MHz, 298 K, $C_6D_{12}$) of (BDI*)$MgNa_4N''_3(N_2O_2)$ (**6**).

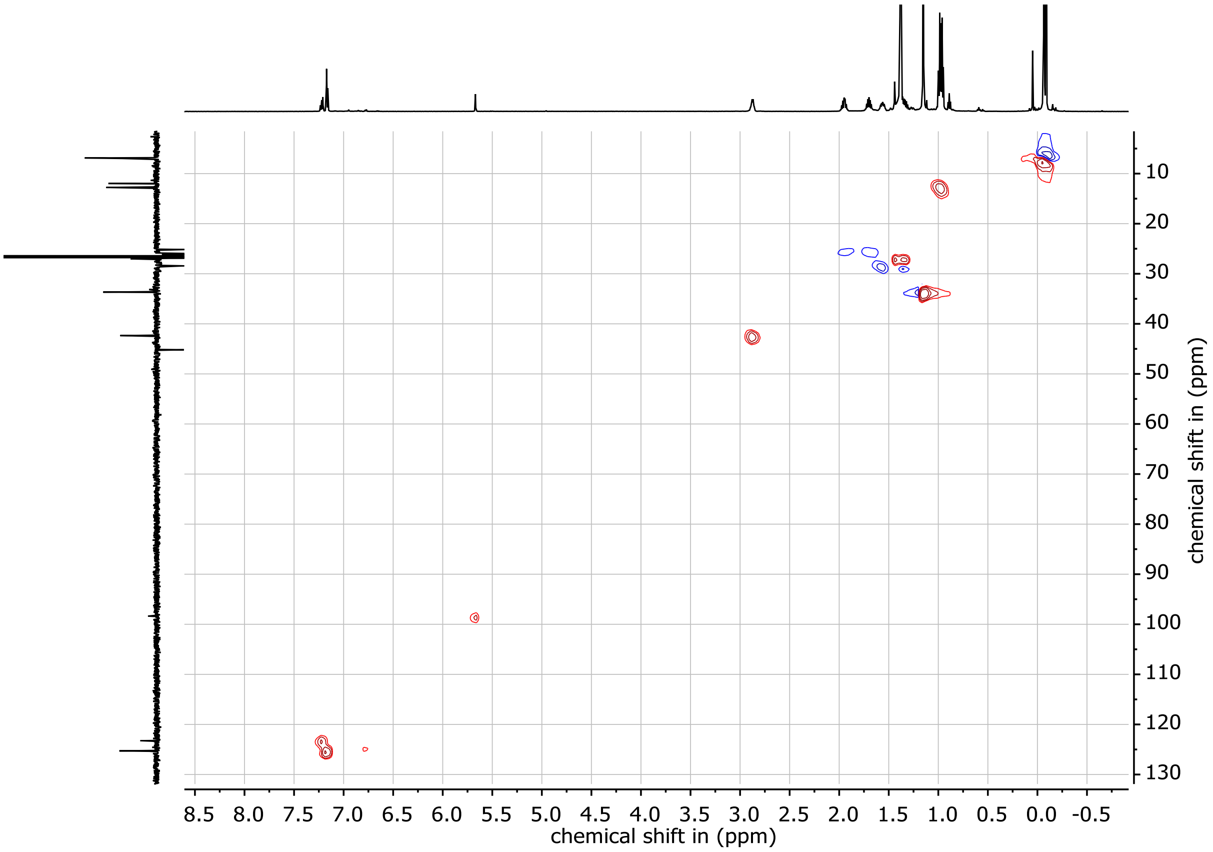


**Figure 31.** 2D-HSQC NMR spectrum (600/151 MHz, 298 K, $C_6D_{12}$) of (BDI*)$MgNa_4N''_3(N_2O_2)$ (**6**).

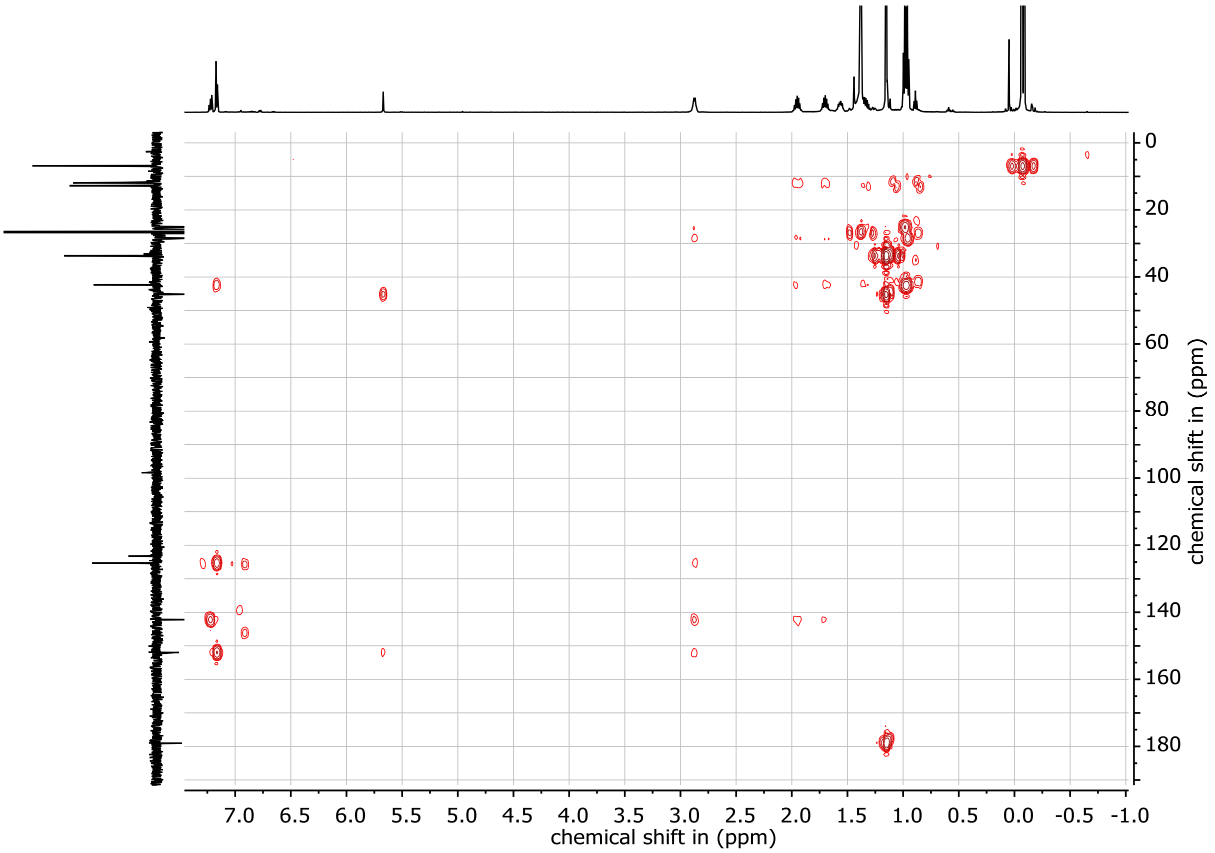


**Figure 32.** 2D-HMBC NMR spectrum (600/151 MHz, 298 K, $C_6D_{12}$) of (BDI*)$MgNa_4N''_3(N_2O_2)$ (**6**).

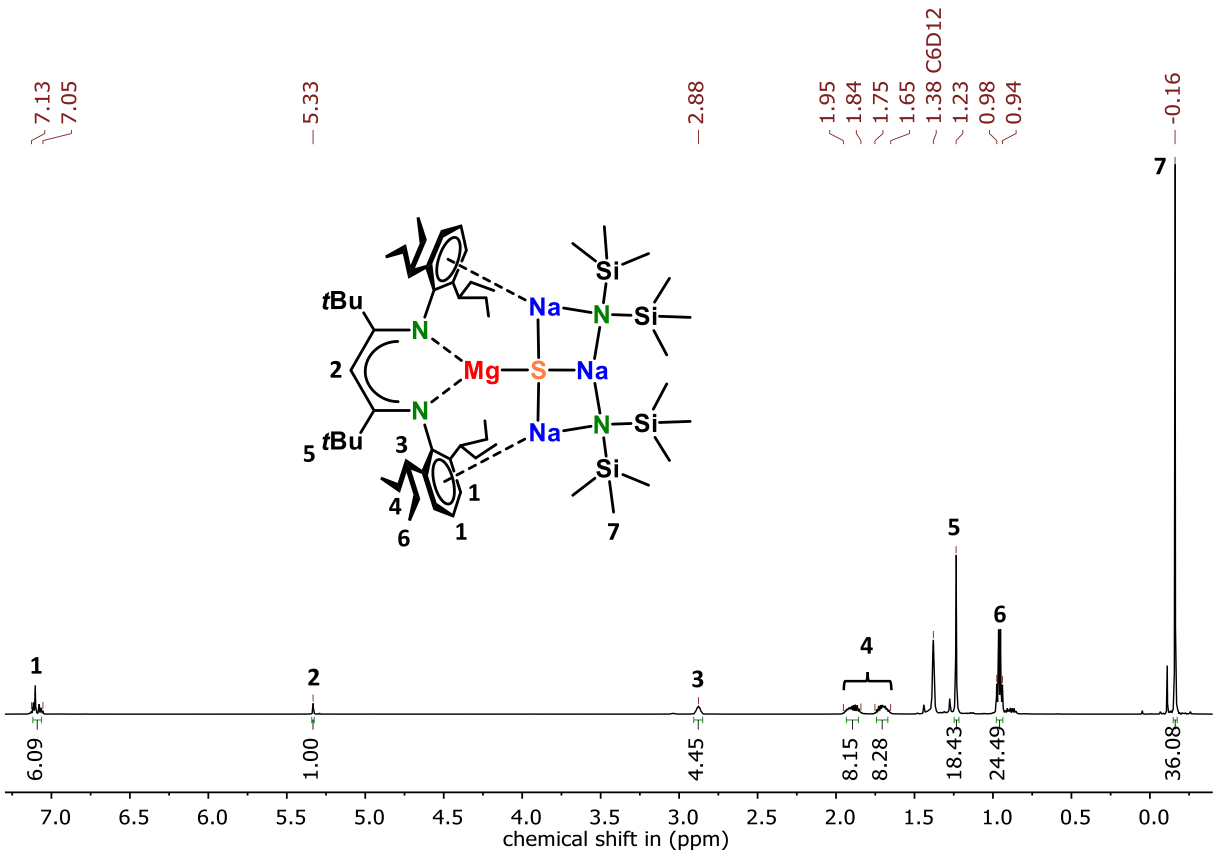


**Figure 33.** $^1$H NMR (600 MHz, 298 K, $C_6D_{12}$) spectrum of (BDI*)$MgNa_3N''_2S$ (**7**).

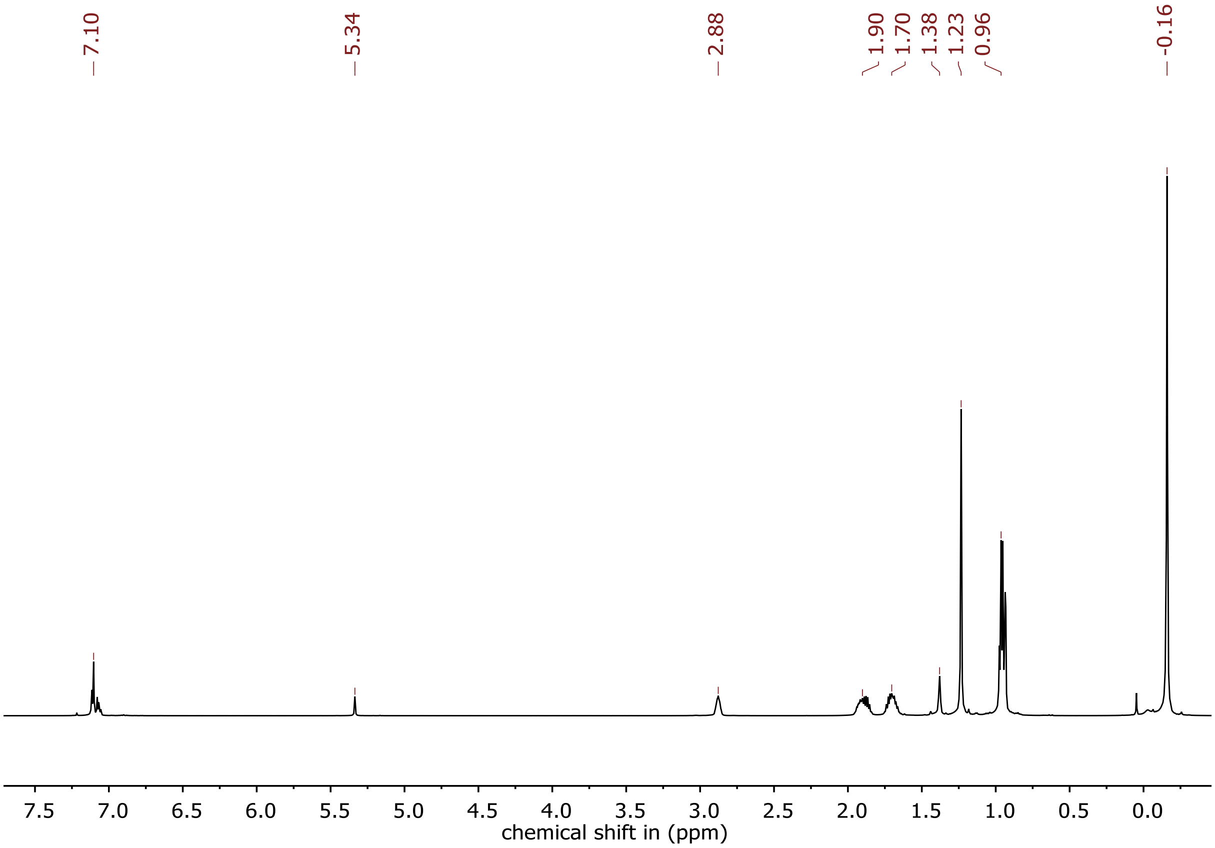


**Figure 34.** $^{1}$H NMR spectrum (600 MHz, 298 K, $C_6D_{12}$) of crude (BDI*)$MgNa_3N''_2S$ (**7**).

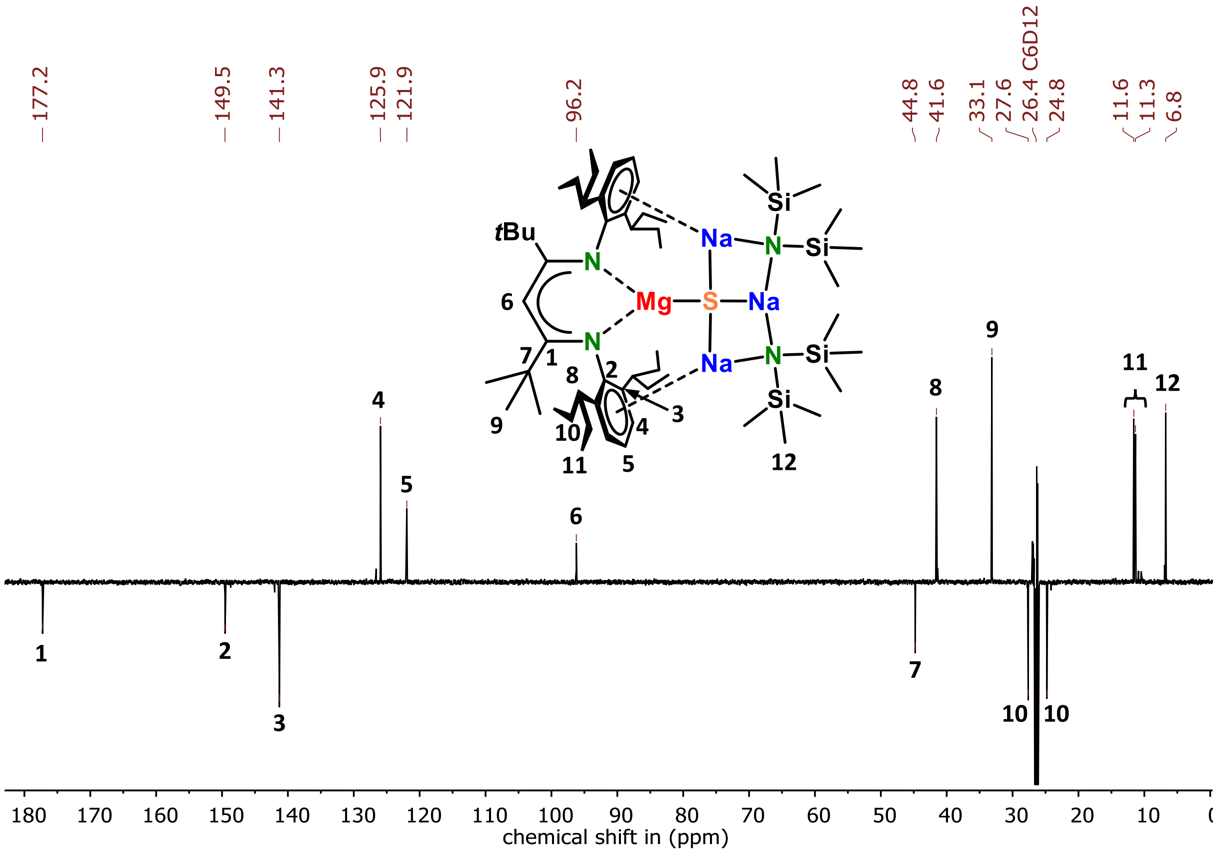


**Figure 35.** $^{13}$C-APT NMR (151 MHz, 298 K, $C_6D_{12}$) spectrum of (BDI*)$MgNa_3N''_2S$ (**7**).

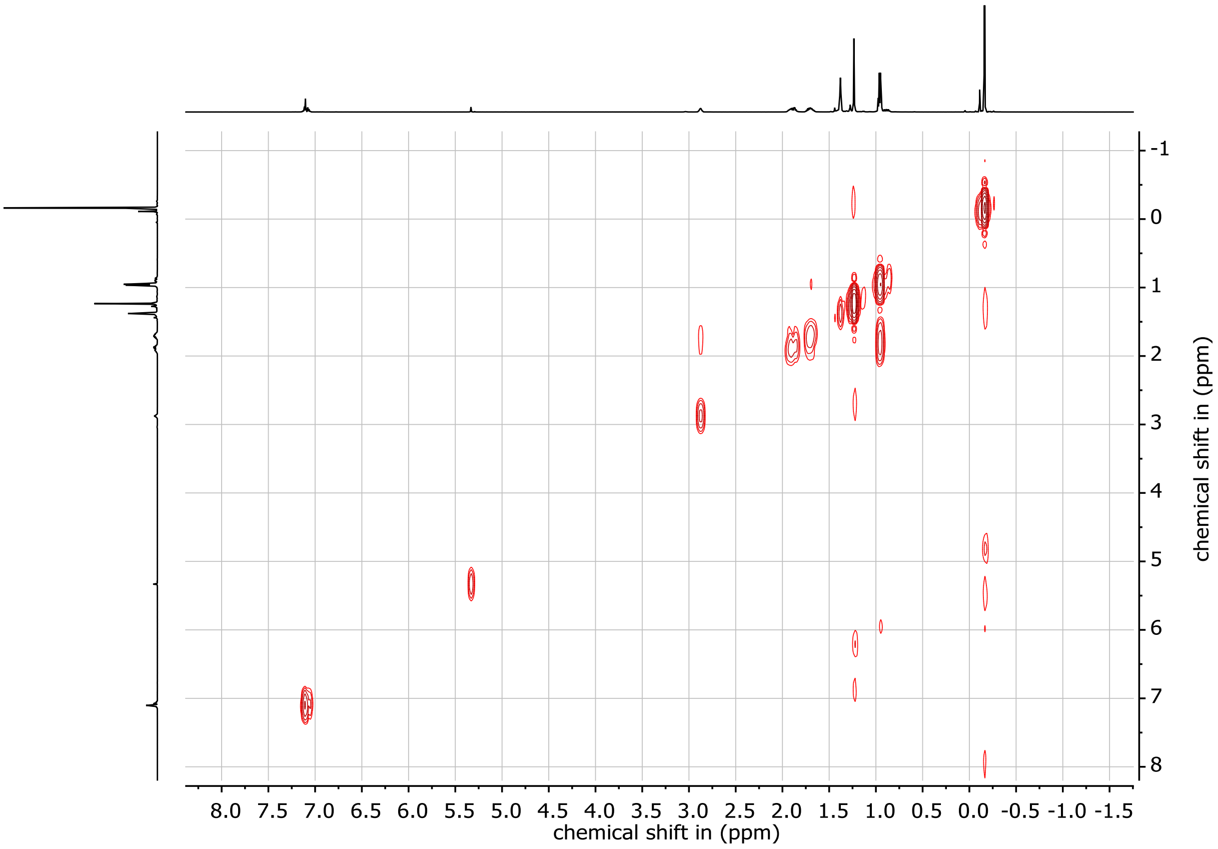


**Figure 36.** 2D-COSY NMR (600 MHz, 298 K, $C_6D_{12}$) spectrum of (BDI*)$MgNa_3N''_2S$ (**7**).

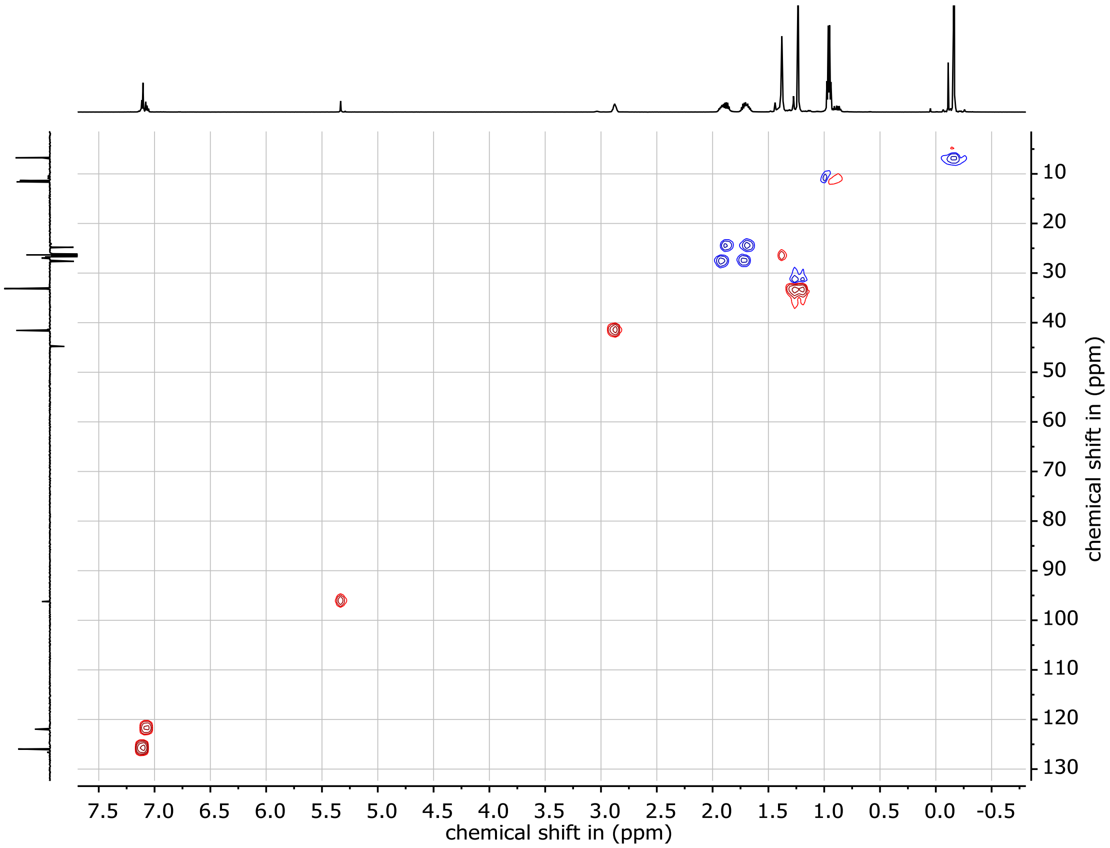


**Figure 37.** 2D-HSQC NMR spectrum (600/151 MHz, 298 K, $C_6D_{12}$) of (BDI*)$MgNa_3N''_2S$ (**7**).

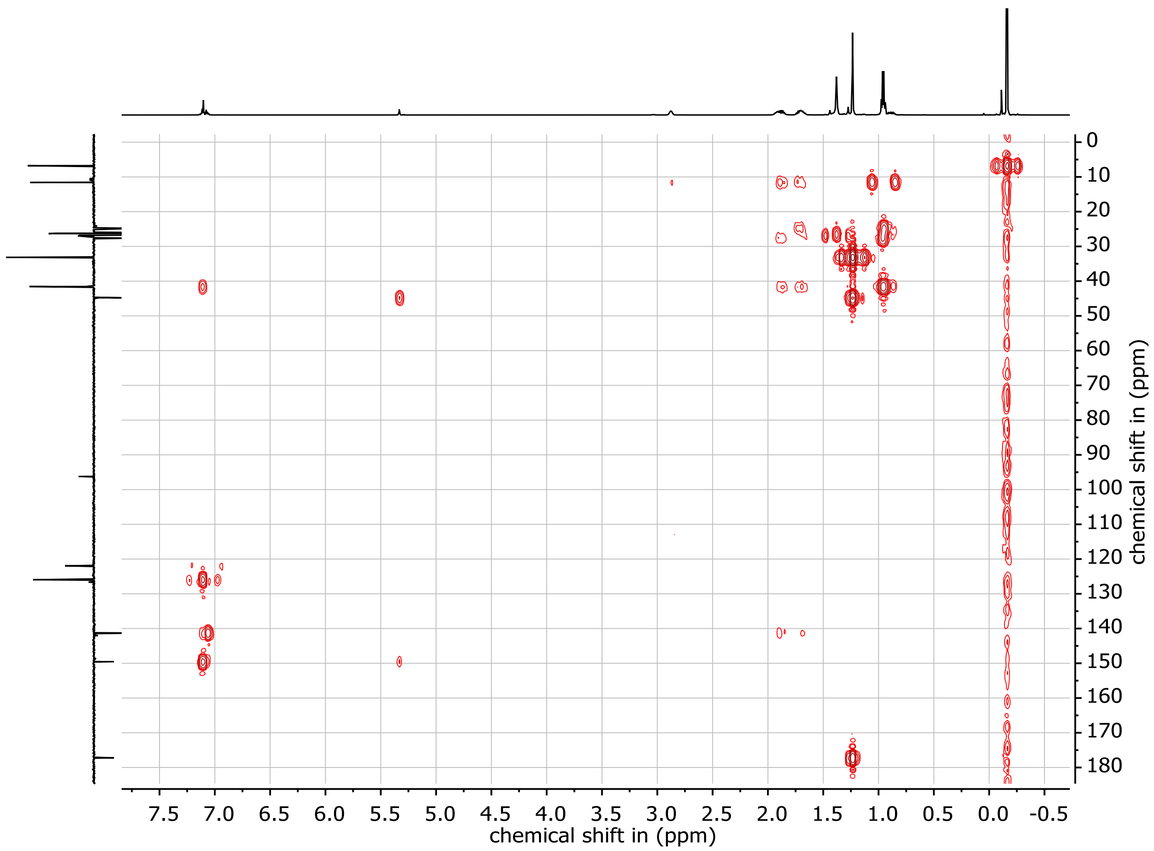


**Figure 38.** 2D-HMBC NMR spectrum (600/151 MHz, 298 K, $C_6D_{12}$) of (BDI*)MgNa$_3$N''$_2$S (**7**).

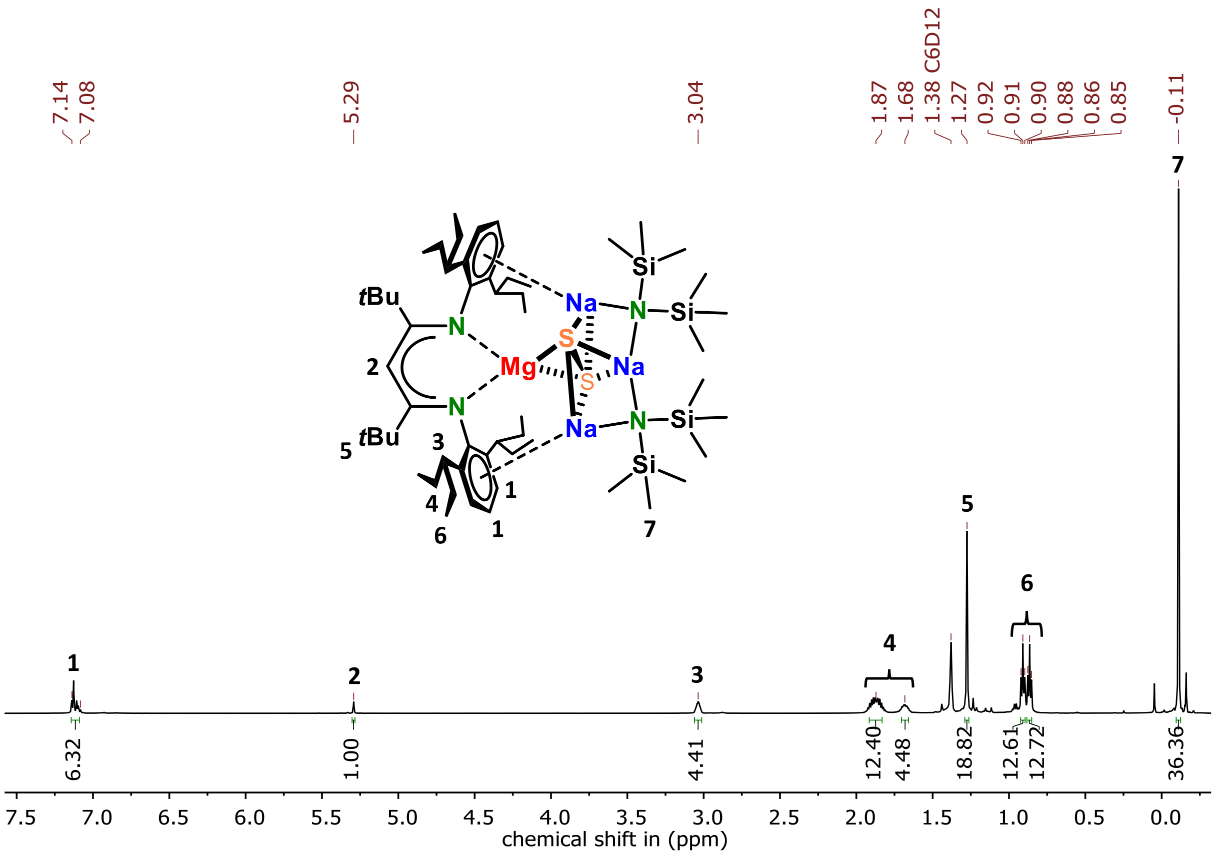


**Figure 39.** $^{1}$H NMR (600 MHz, 298 K, $C_6D_{12}$) spectrum of (BDI*)MgNa$_3$N''$_2$(S$_2$) (**8**).

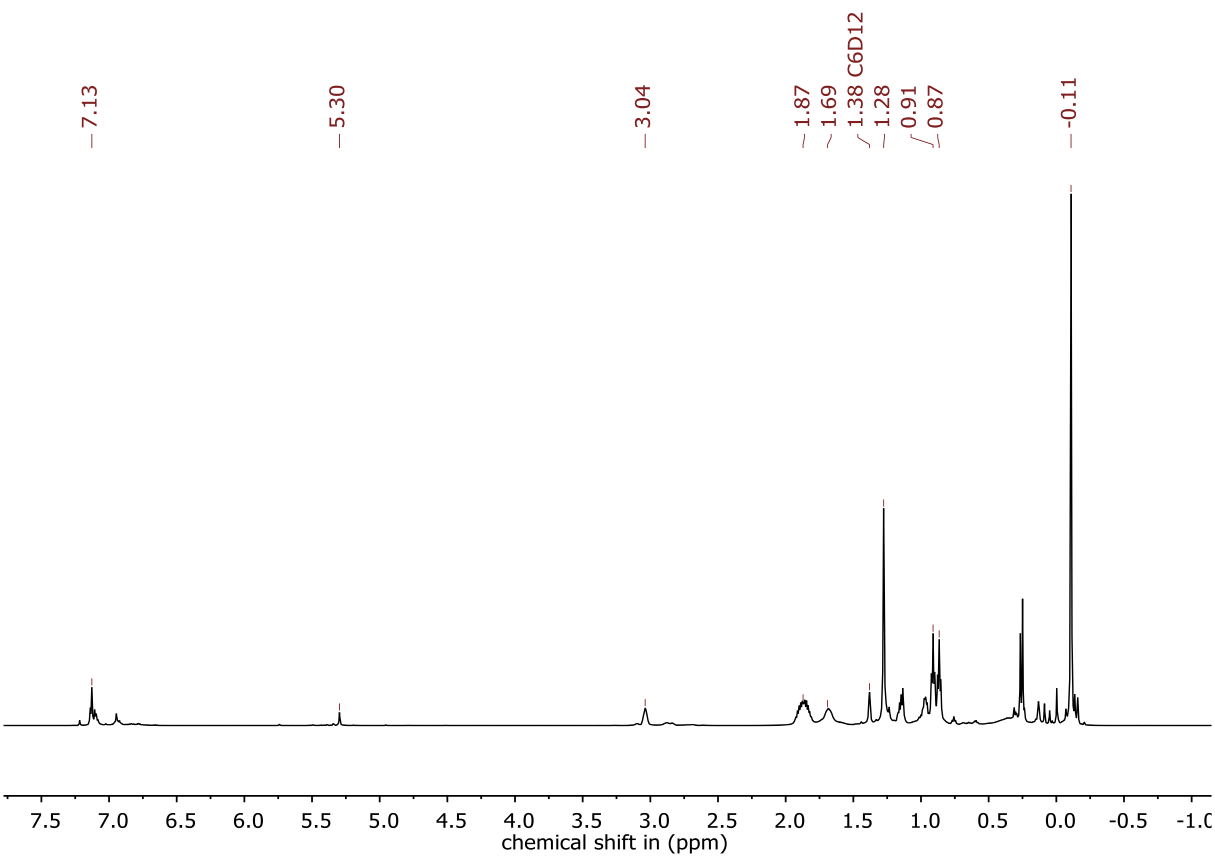


**Figure 40.** $^{1}$H NMR (600 MHz, 298 K, $C_6D_{12}$) spectrum of crude (BDI*)$MgNa_3N''_2(S_2)$ (**8**).

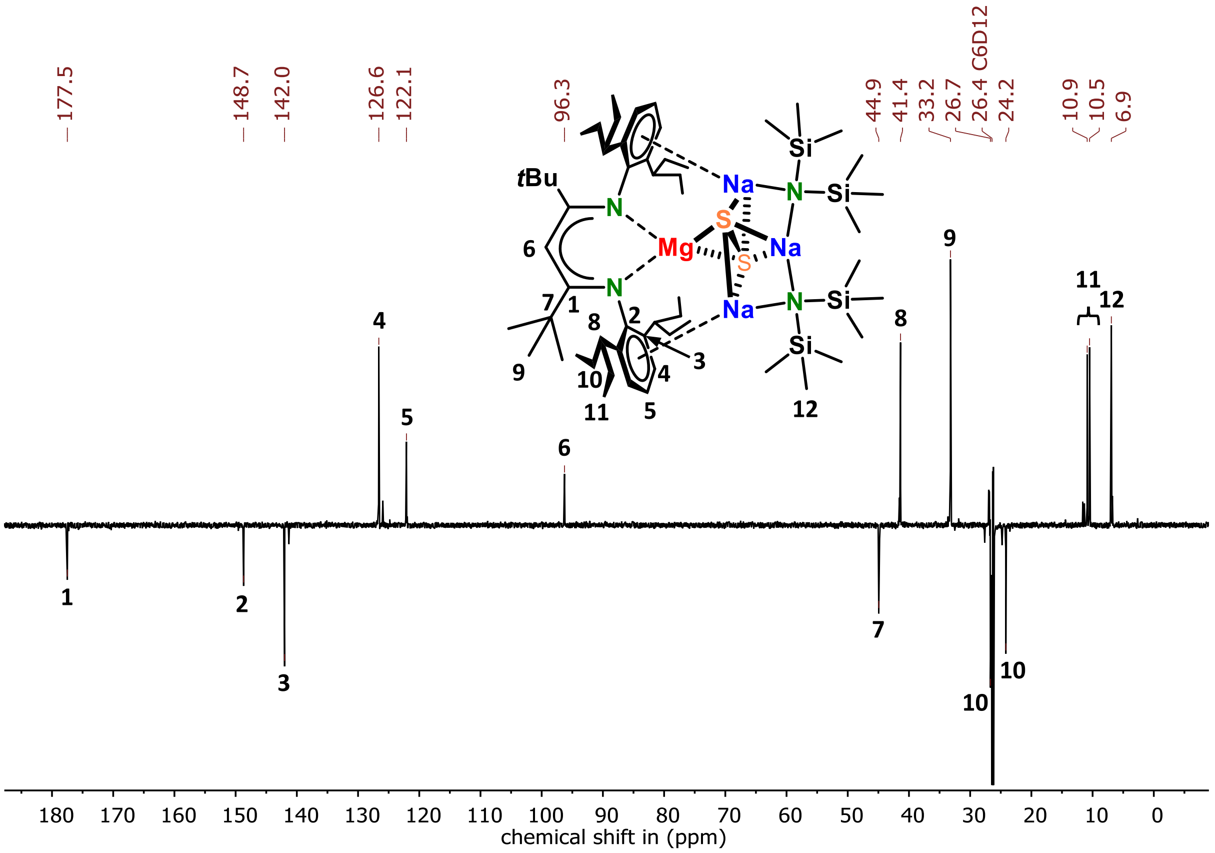


**Figure 41.** $^{13}$C-APT NMR (151 MHz, 298 K, $C_6D_{12}$) spectrum of (BDI*)$MgNa_3N''_2(S_2)$ (**8**).

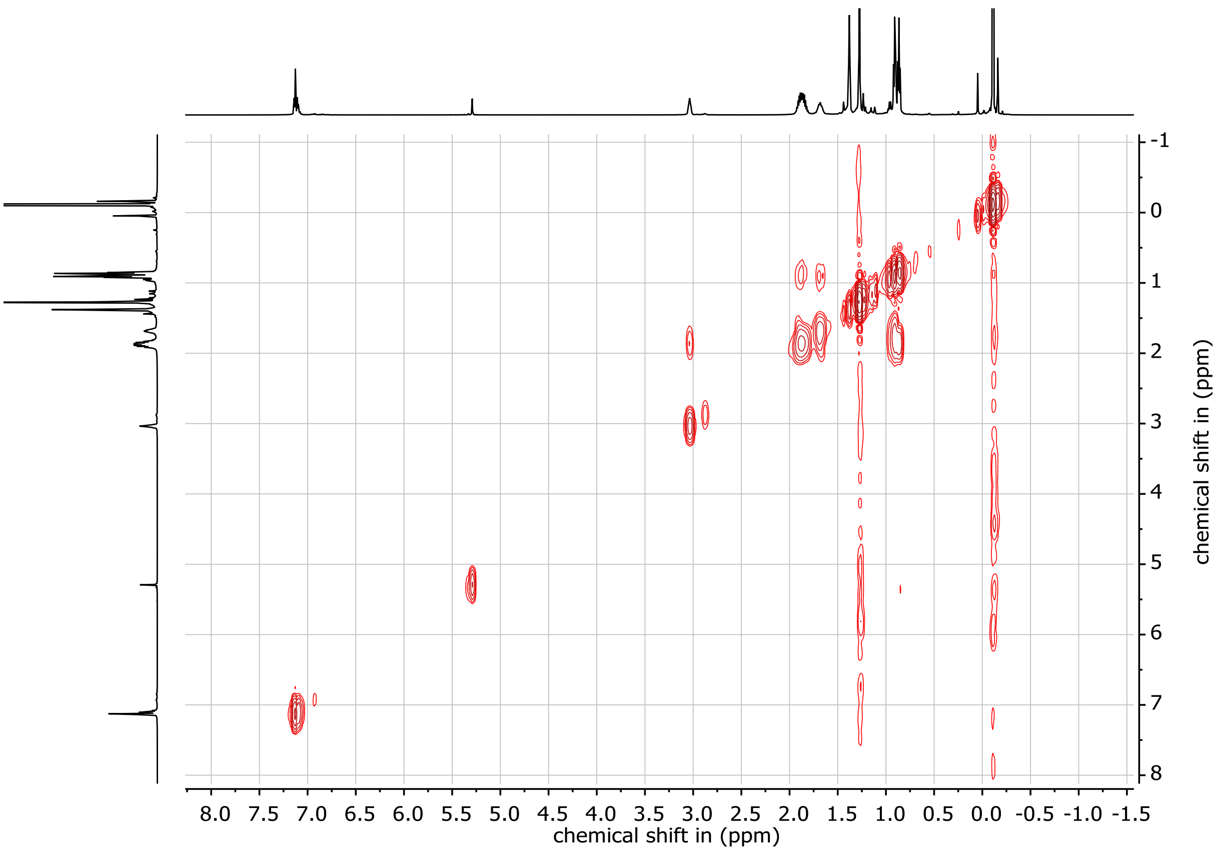


**Figure 42.** 2D-COSY NMR (600 MHz, 298 K, $C_6D_{12}$) spectrum of (BDI*)MgNa$_3$N''$_2$(S$_2$) (**8**).

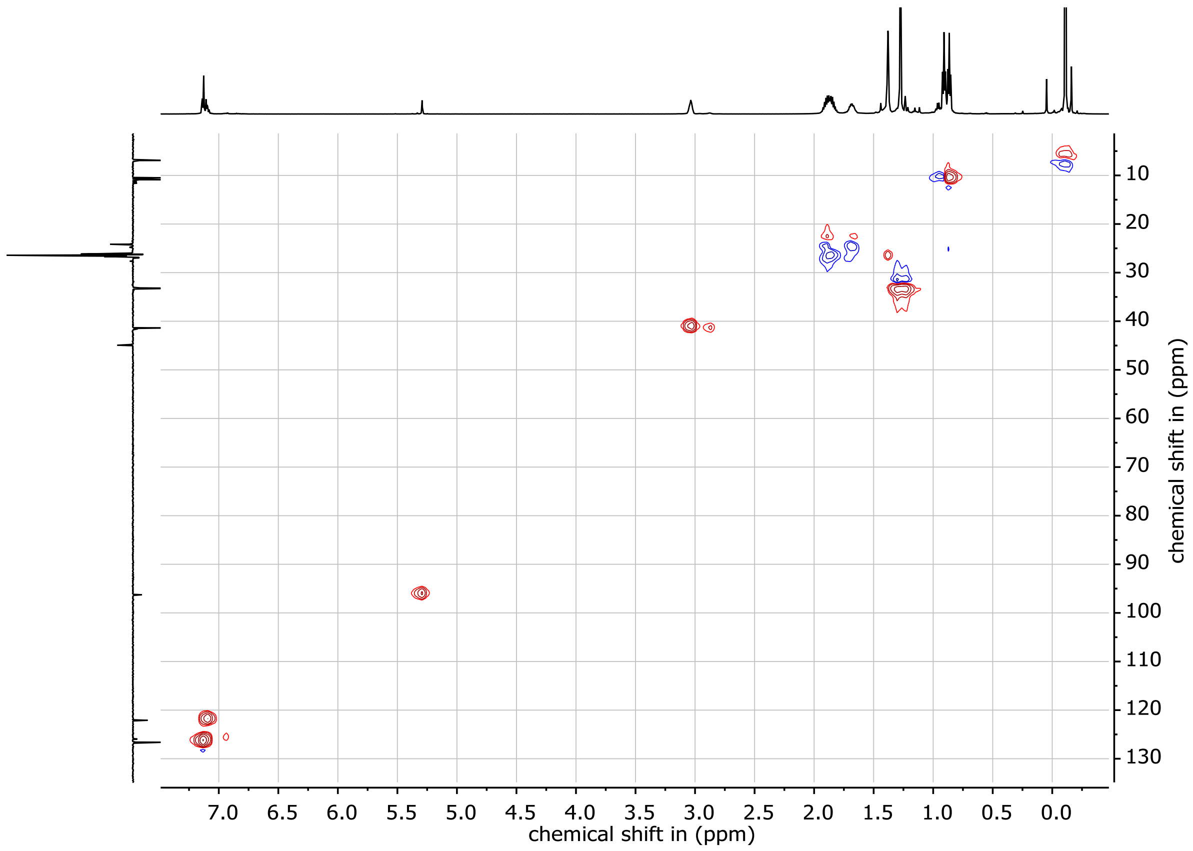


**Figure 43.** 2D-HSQC NMR spectrum (600/151 MHz, 298 K, $C_6D_{12}$) of (BDI*)MgNa$_3$N''$_2$(S$_2$) (**8**).

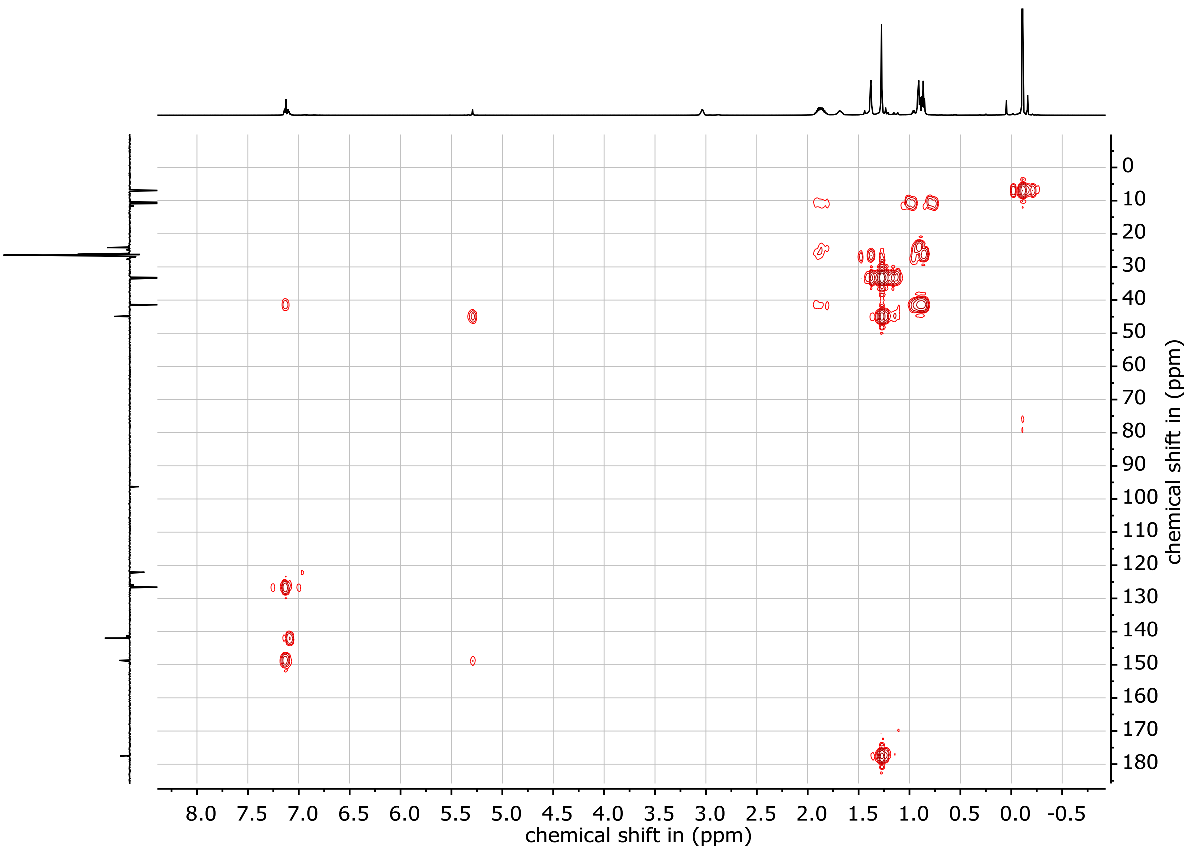


**Figure 44.** 2D-HMBC NMR spectrum (600/151 MHz, 298 K, $C_6D_{12}$) of (BDI*)$MgNa_3N''_2(S_2)$ (**8**).

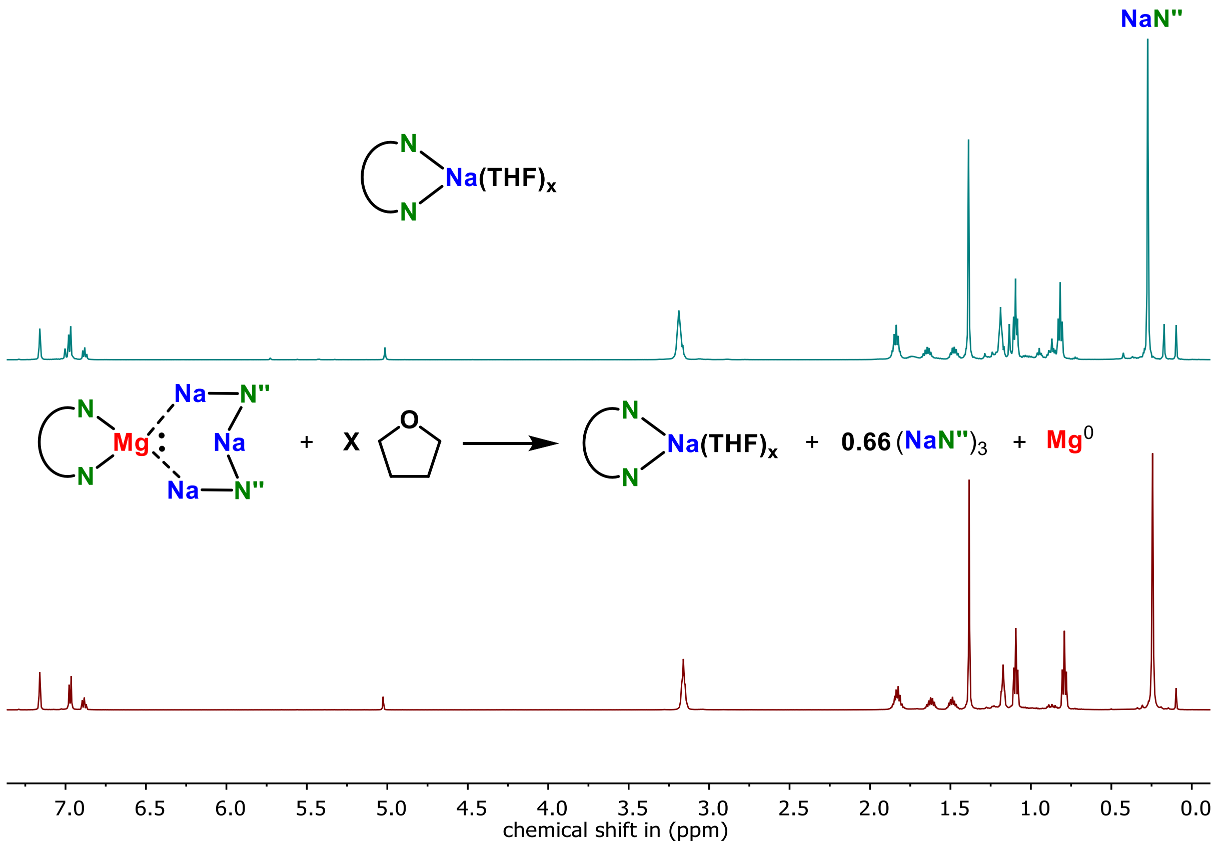


**Figure 45.** $^{1}$H NMR spectrum (600 MHz, 298 K, $C_6D_6$) after stirring a solution of (BDI*)$MgNa_3N''_2$ (**1**) in THF for 1 min (bottom). The signals correspond with those of a mixture of (BDI*)Na$(THF)_x$ and $(NaN'')_3$ (top).

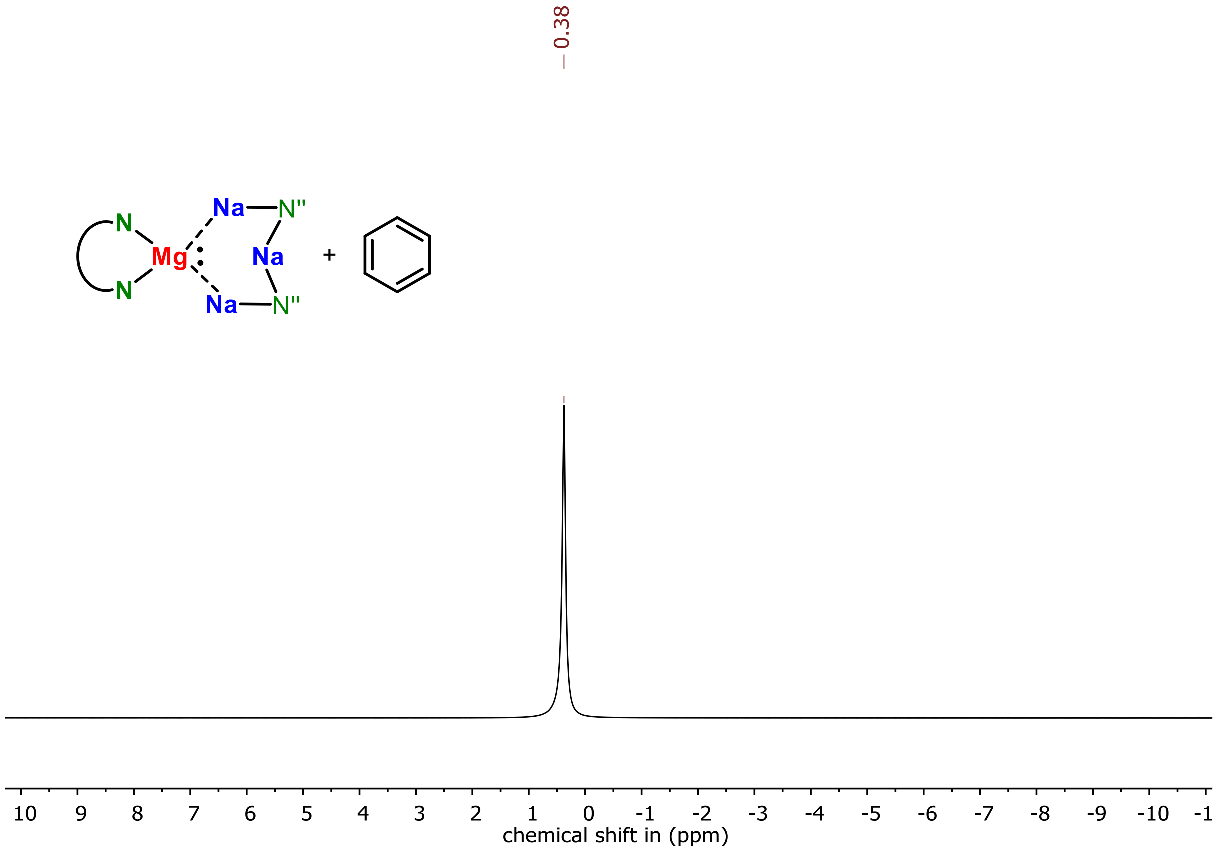


**Figure 46.** $^{23}$Na NMR spectrum (600 MHz, 298 K, $D_2O$) of the formed metal mirror quenched in $D_2O$ after stirring a solution of (BDI*)$MgNa_3N''_2$ (**1**) in benzene for 7 days.

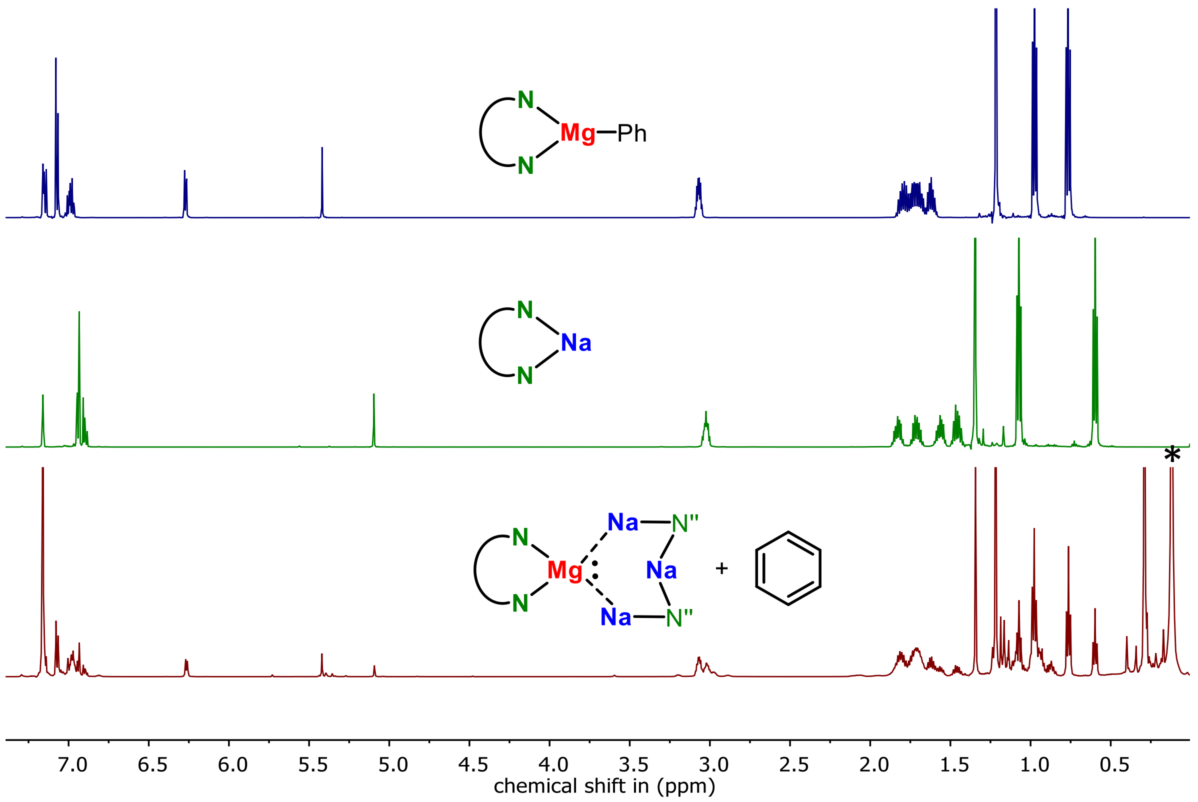


**Figure 47.** $^{1}$H NMR spectrum (600 MHz, 298 K, $C_6D_6$) after stirring a solution of (BDI*)$MgNa_3N''_2$ (**1**) in benzene for 7 days (bottom). (BDI*)MgPh (top), (BDI*)Na (middle), $(NaN'')_3$ (marked with *) and several other (BDI*) based products can be observed.

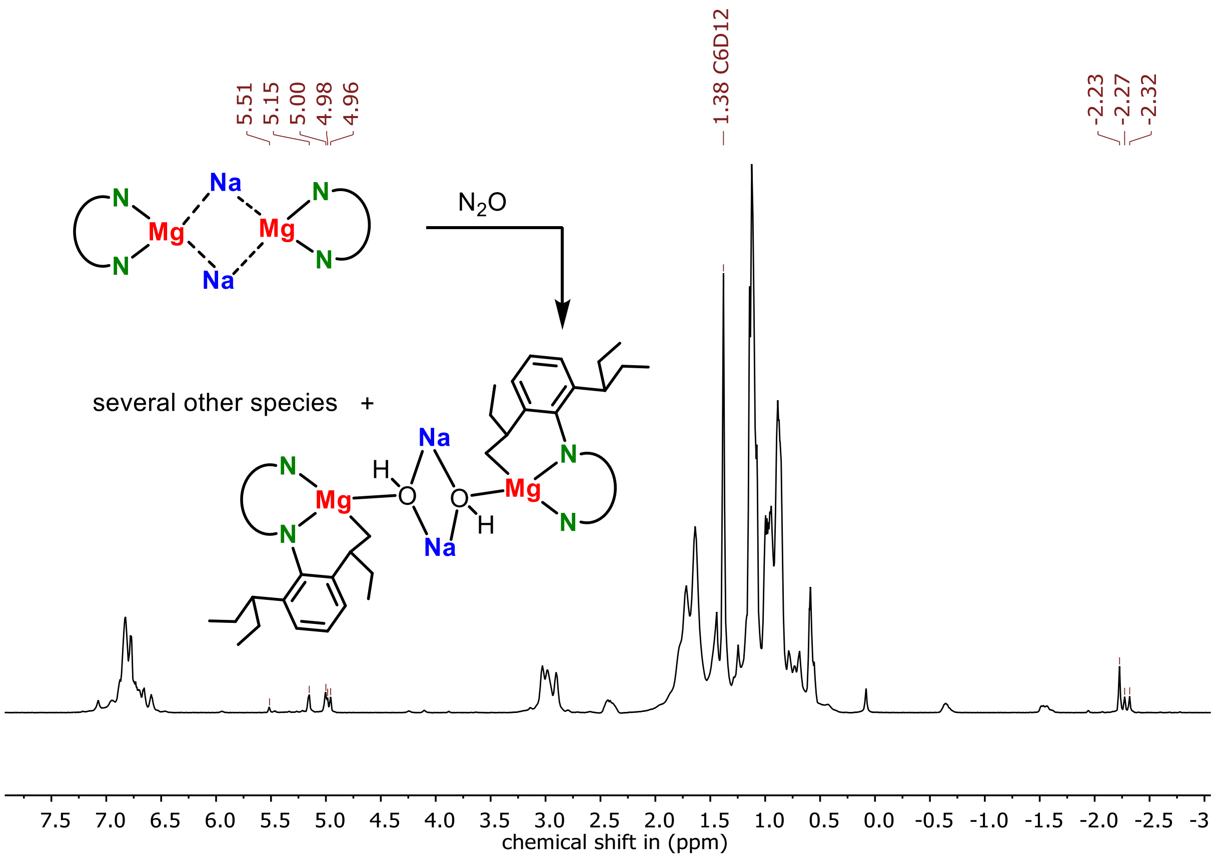


**Figure 48.** $^1$H NMR spectrum (600 MHz, 298 K, $C_6D_{12}$) of the attempted synthesis of [(BDI*)MgO$^-$Na$^+$]$_2$ (**3**) leading to the formation of at least five species, from which only **4** could be identified by single crystal X-ray diffraction.

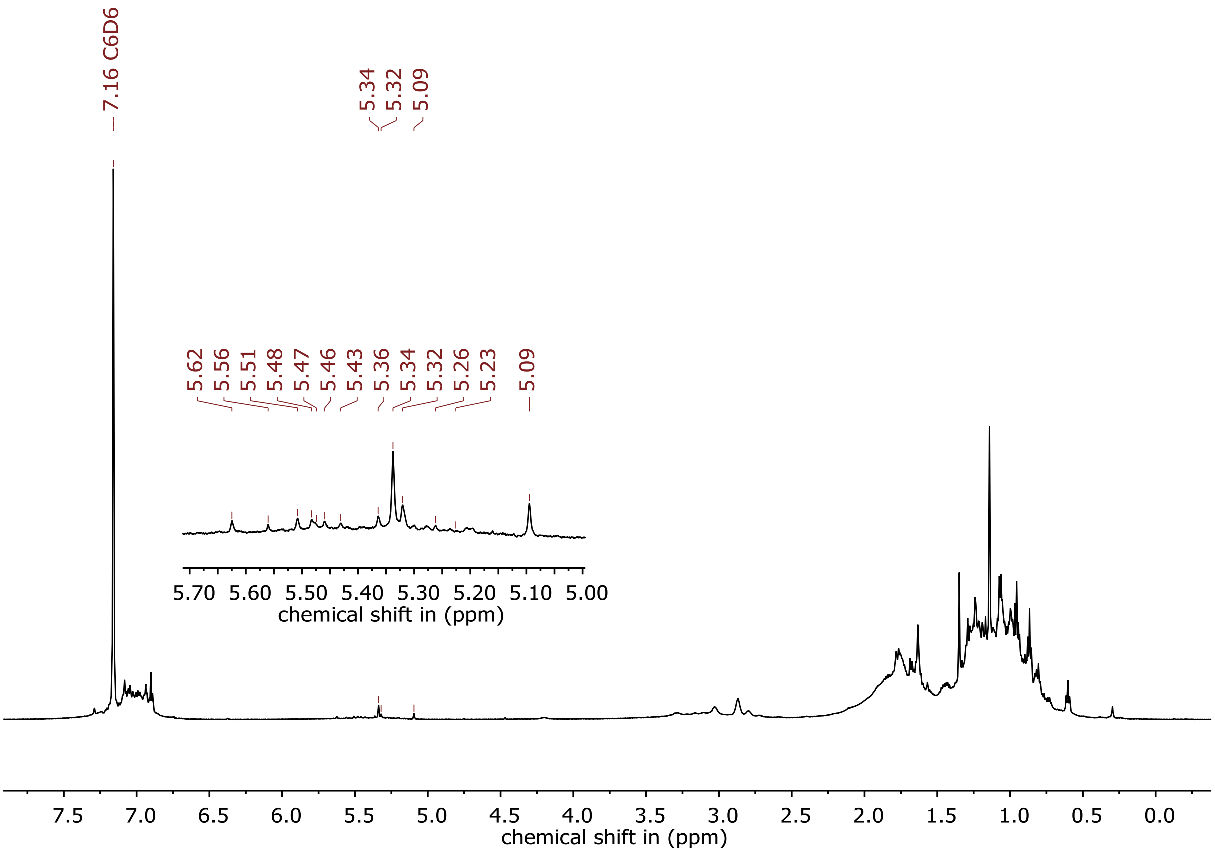


**Figure 49.** $^1$H NMR spectrum (600 MHz, 298 K, $C_6D_6$) of the reaction of [(BDI*)MgNa]$_2$ (**I**) with dry air. Very broad signals and multiplets suggest decomposition and formation of various products.

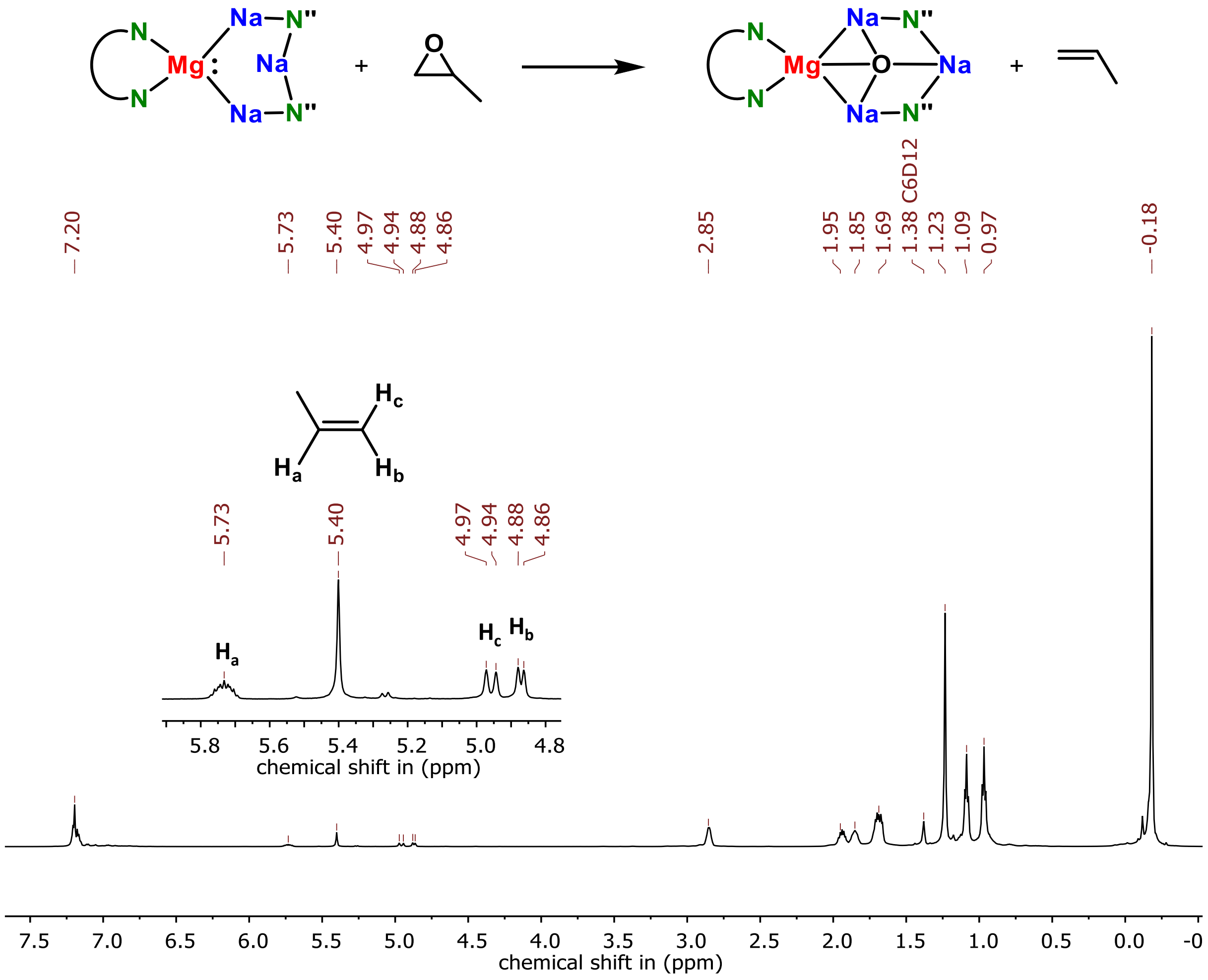


**Figure 50.** $^{1}$H NMR spectrum (600 MHz, 298 K, $C_6D_{12}$) of an equimolar reaction of (BDI*)$MgNa_3N''_2$ (**1**) with propylene oxide. (BDI*)$MgNa_3N''_2O$ (**2**) and propylene are formed quantitatively.

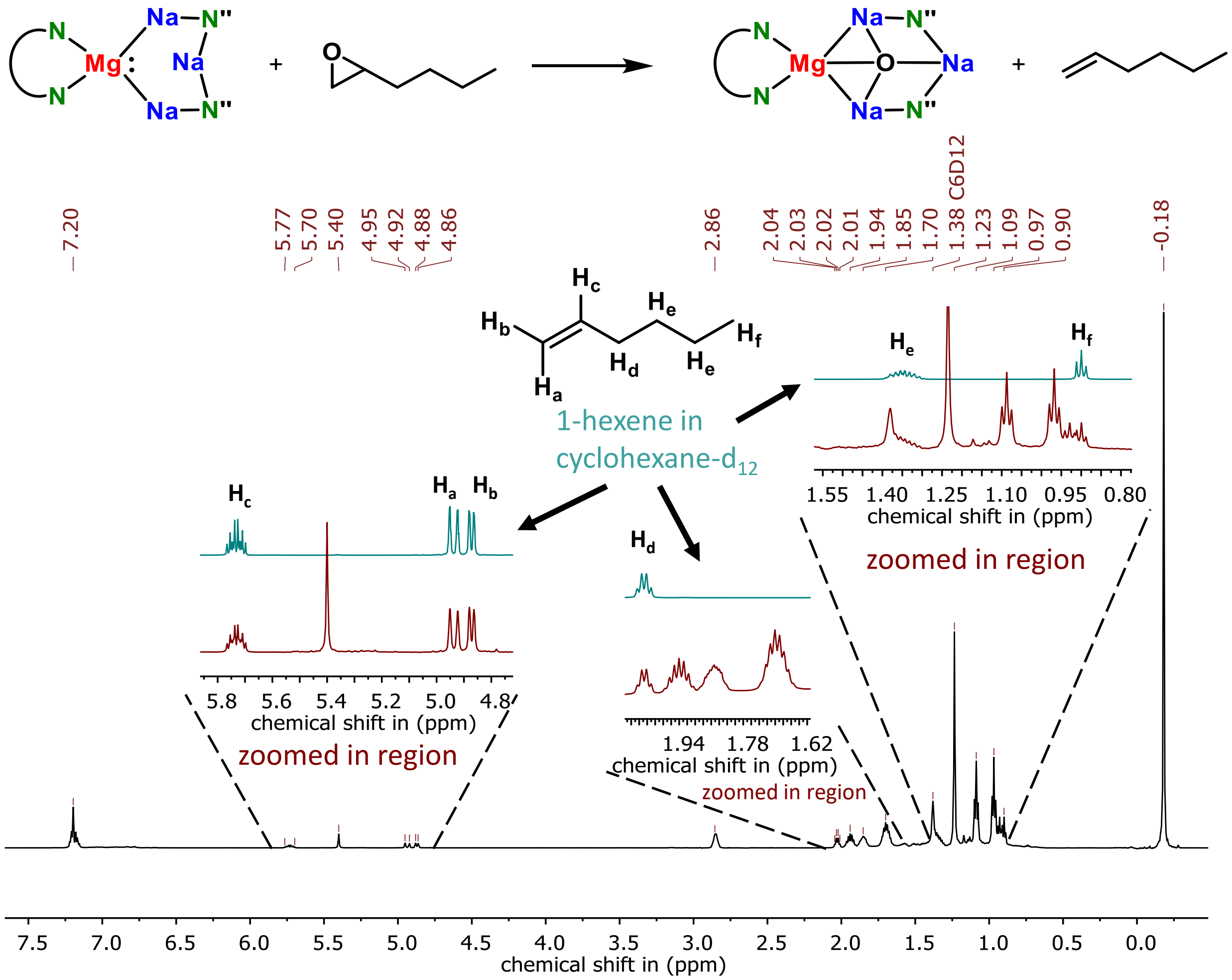


**Figure 51.** $^{1}$H NMR spectrum (600 MHz, 298 K, $C_6D_{12}$) of an equimolar reaction of (BDI*)MgNa$_3$N''$_2$ (**1**) with 1,2-epoxy hexane. (BDI*)MgNa$_3$N''$_2$O (**2**) and 1-hexene are formed quantitatively.

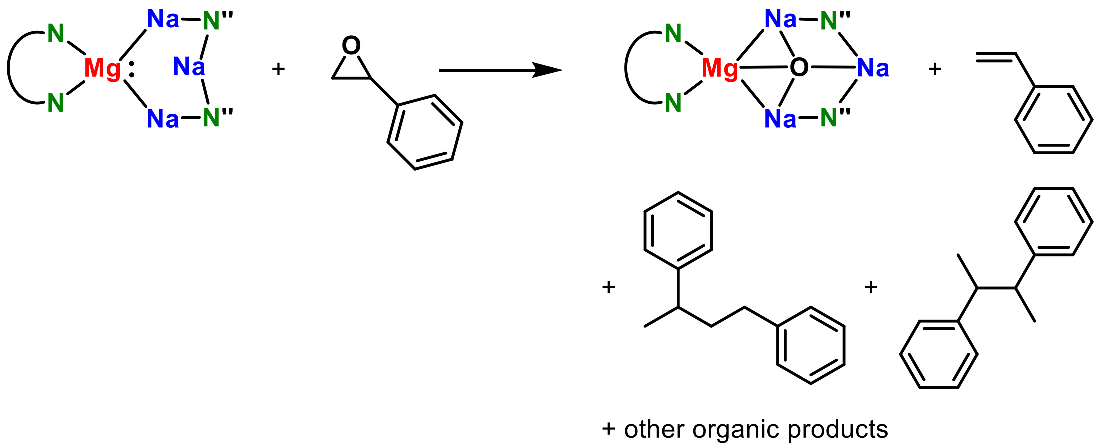


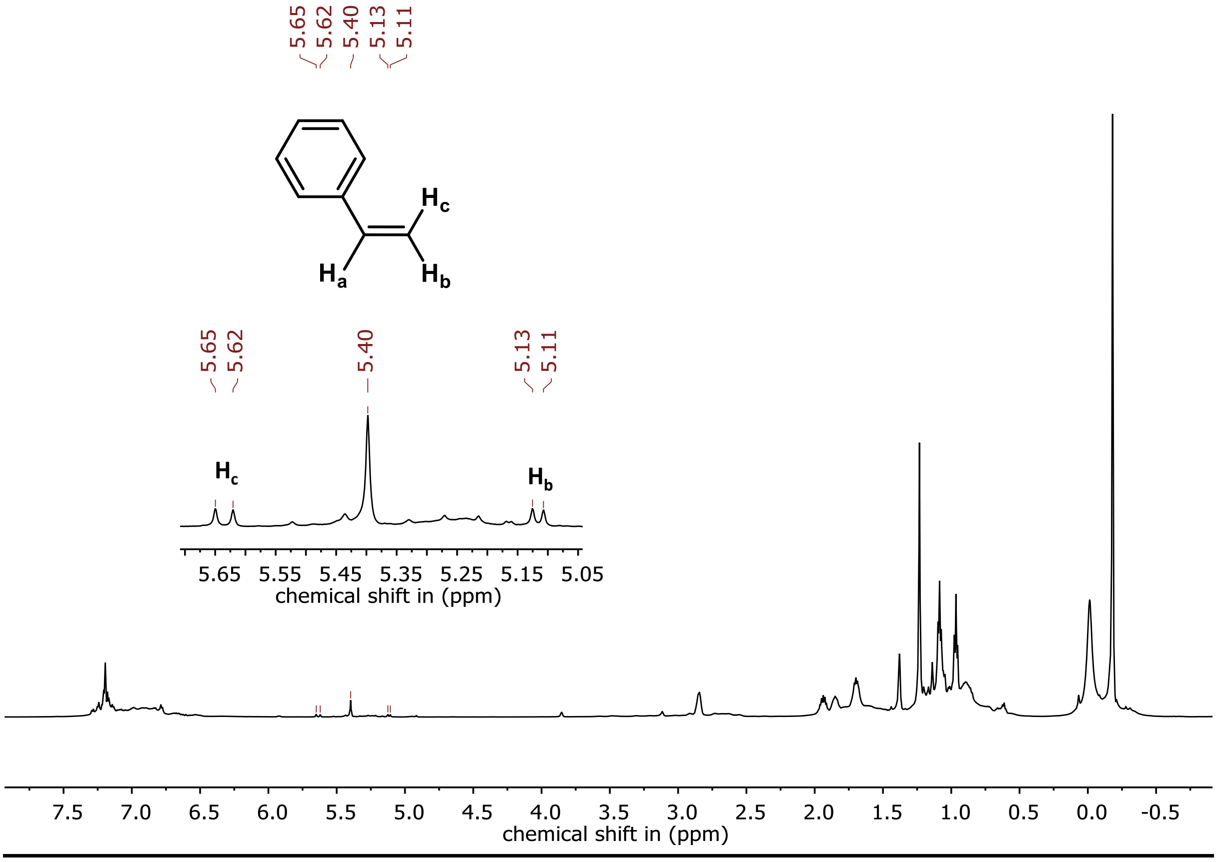


**Figure 52.** $^{1}$H NMR spectrum (600 MHz, 298 K, $C_6D_{12}$) of an equimolar reaction of (BDI*)$MgNa_3N''_2$ (**1**) with styrene oxide. (BDI*)$MgNa_3N''_2O$ (**2**) is formed quantitatively. Only approx. 20% styrene can be detected by $^{1}$H NMR spectroscopy, which we attribute to oligomerization of styrene.

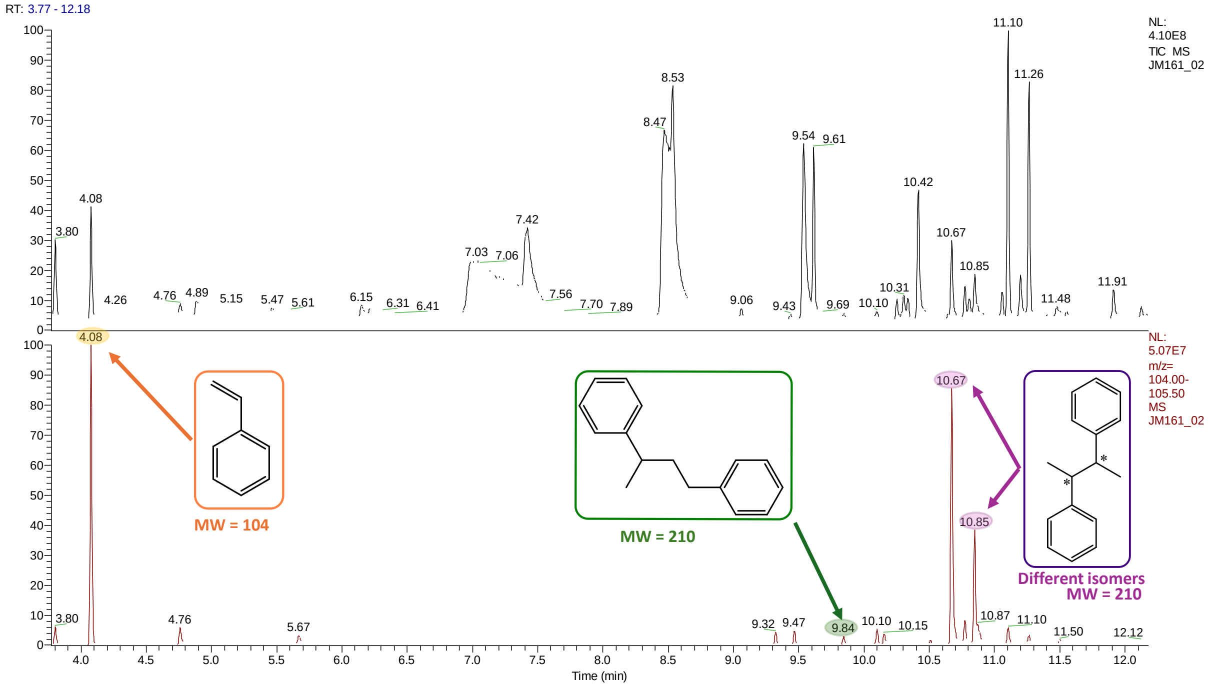


**Figure 53.** GC-MS Chromatogram of the reaction of (BDI*)MgNa$_3$N''$_2$ (**1**) with styrene oxide. After completion of the reaction, the reaction mixture was exposed to air. The chromatogram shows a peak at a retention time (RT) of 4.08 min, 9.84 min, 10.67 min and 10.85 min, which can be attributed to styrene and its oligomerization products.

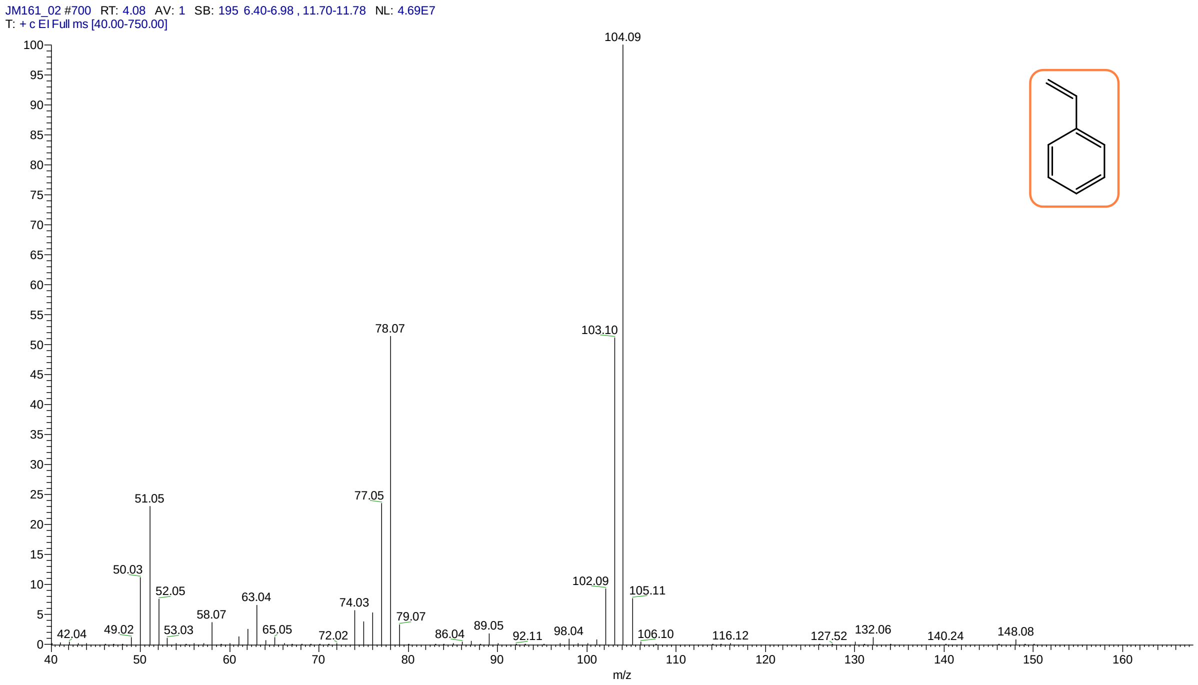


**Figure 54.** Mass spectrum (RT = 4.08 min) of the reaction of (BDI*)MgNa$_3$N''$_2$ (**1**) with styrene oxide.

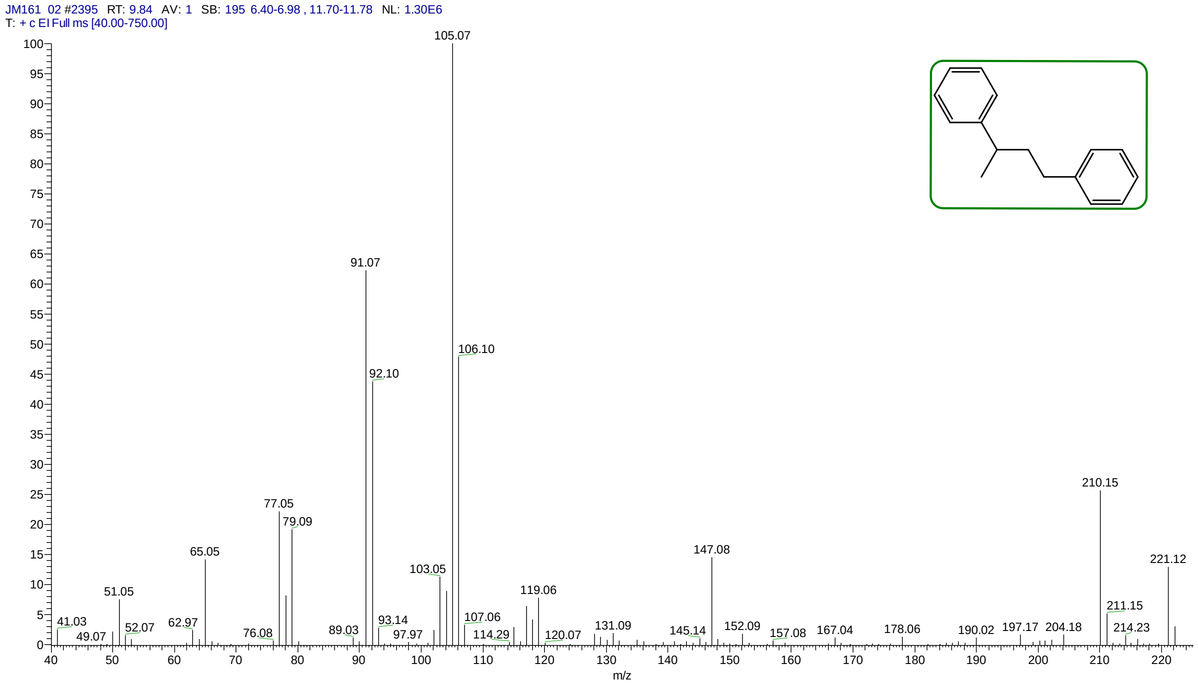


**Figure 55.** Mass spectrum (RT = 9.84 min) of the reaction of (BDI*)MgNa$_3$N''$_2$ (**1**) with styrene oxide.

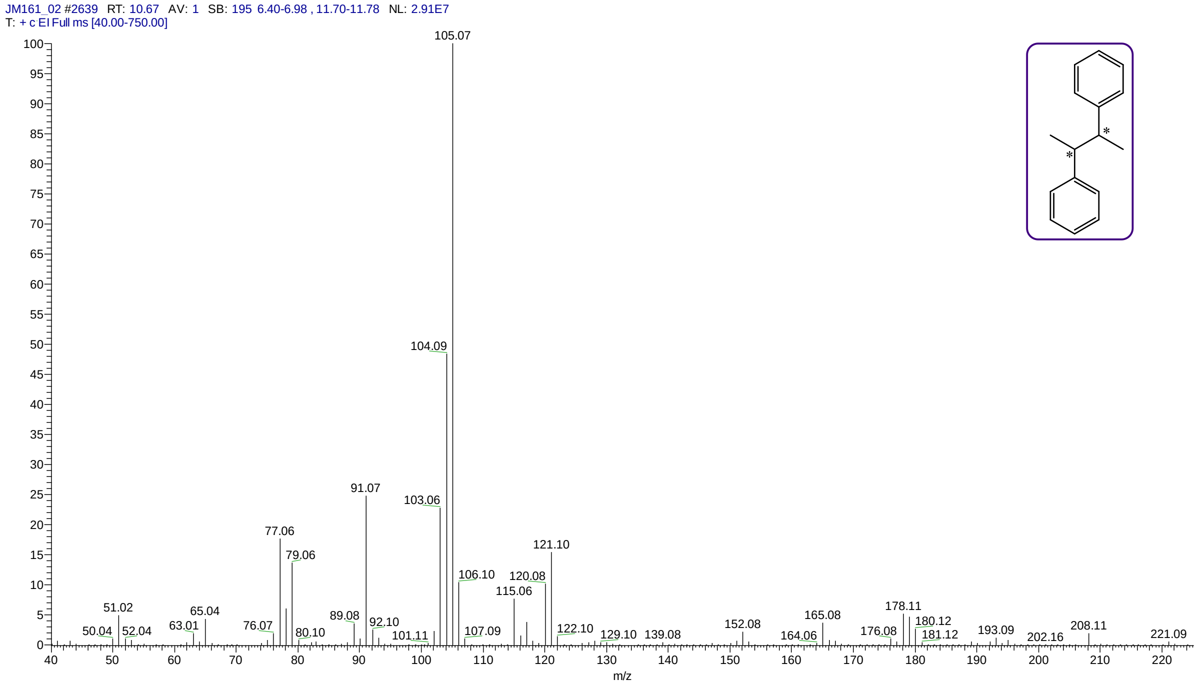


**Figure 56.** Mass spectrum (RT = 10.67 min) of the reaction of (BDI*)MgNa$_3$N''$_2$ (**1**) with styrene oxide.

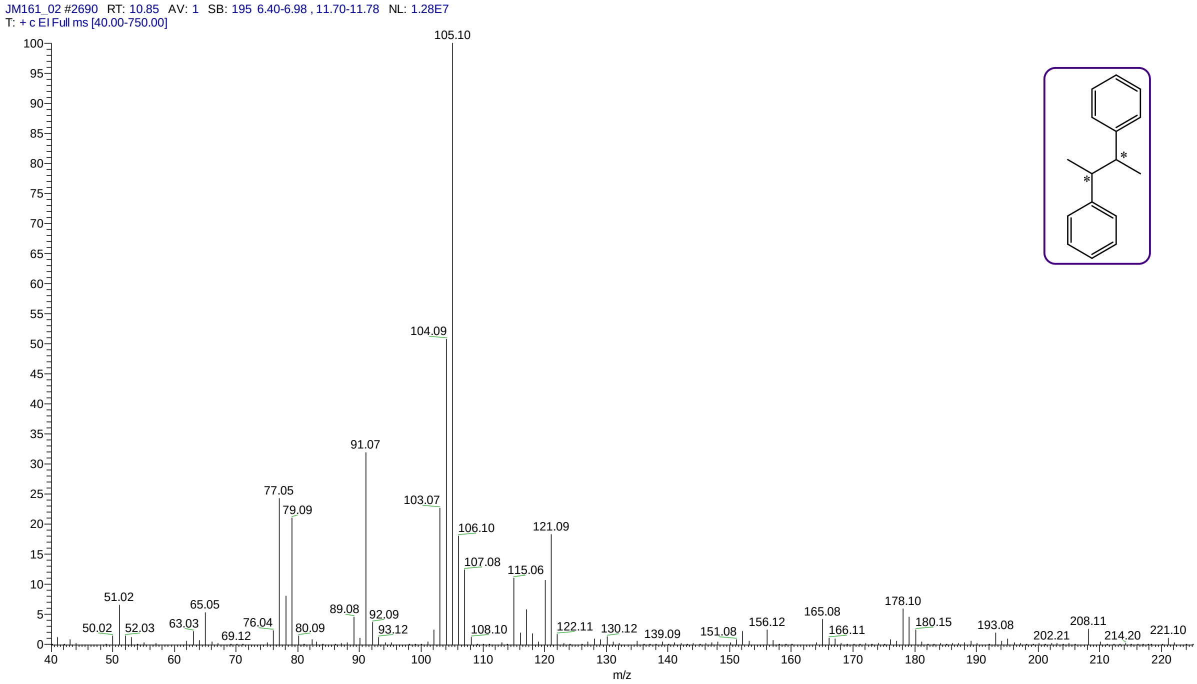


**Figure 57.** Mass spectrum (RT = 10.85 min) of the reaction of (BDI*)MgNa$_3$N''$_2$ (**1**) with styrene oxide.

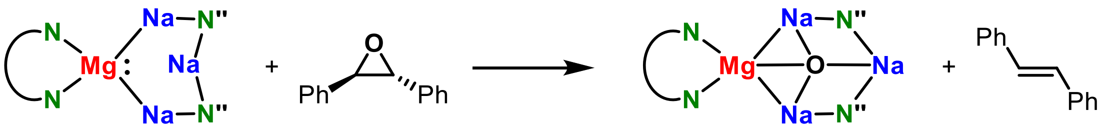

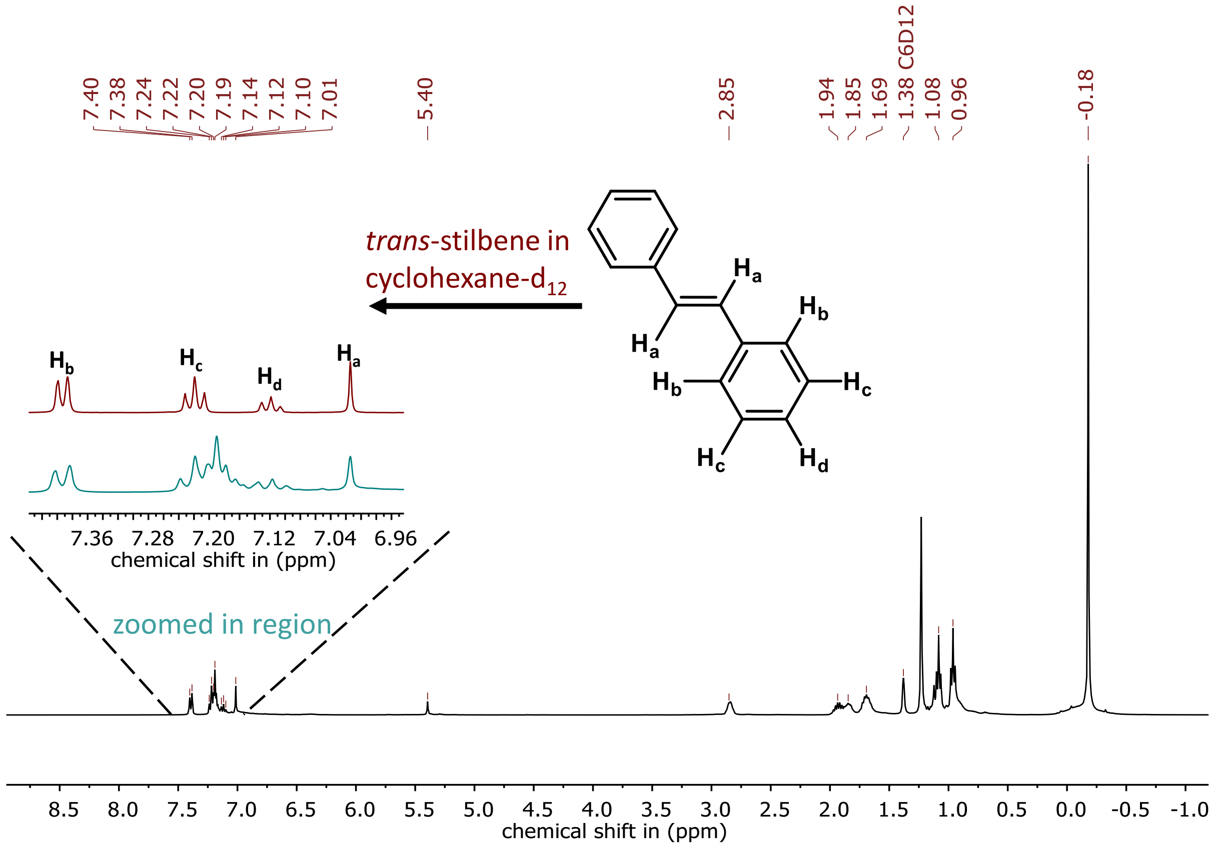


**Figure 58.** $^{1}$H NMR spectrum (400 MHz, 298 K, $C_6D_{12}$) of an equimolar reaction of (BDI*)$MgNa_3N''_2$ (**1**) with *trans*-stilbene oxide. (BDI*)$MgNa_3N''_2O$ (**2**) and *trans*-stilbene are formed quantitatively.

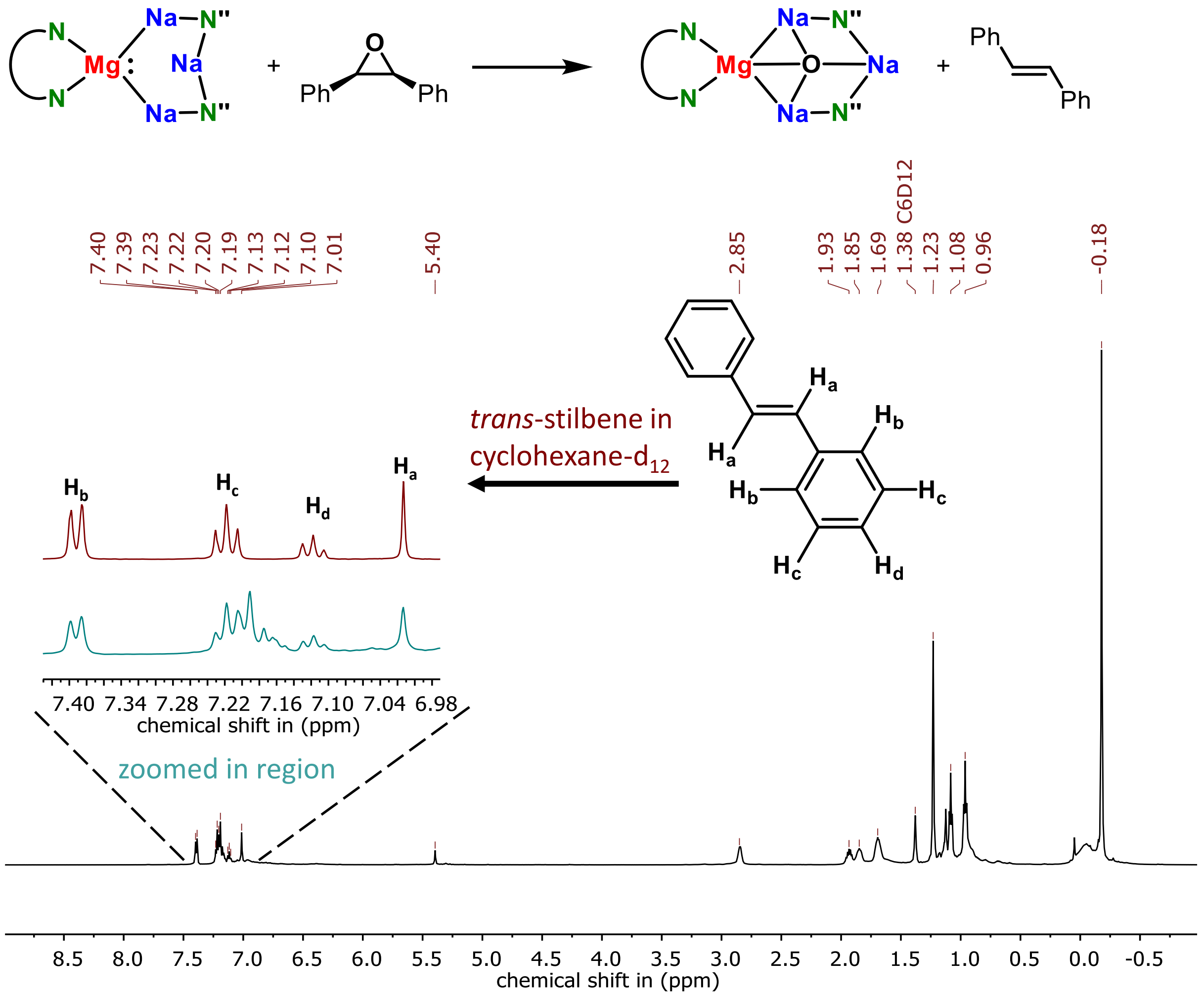


**Figure 59.** $^{1}H$ NMR spectrum (600 MHz, 298 K, $C_6D_{12}$) of an equimolar reaction of (BDI*)$MgNa_3N''_2$ (**1**) with *cis*-stilbene oxide. (BDI*)$MgNa_3N''_2O$ (**2**) and *trans*-stilbene are formed quantitatively.

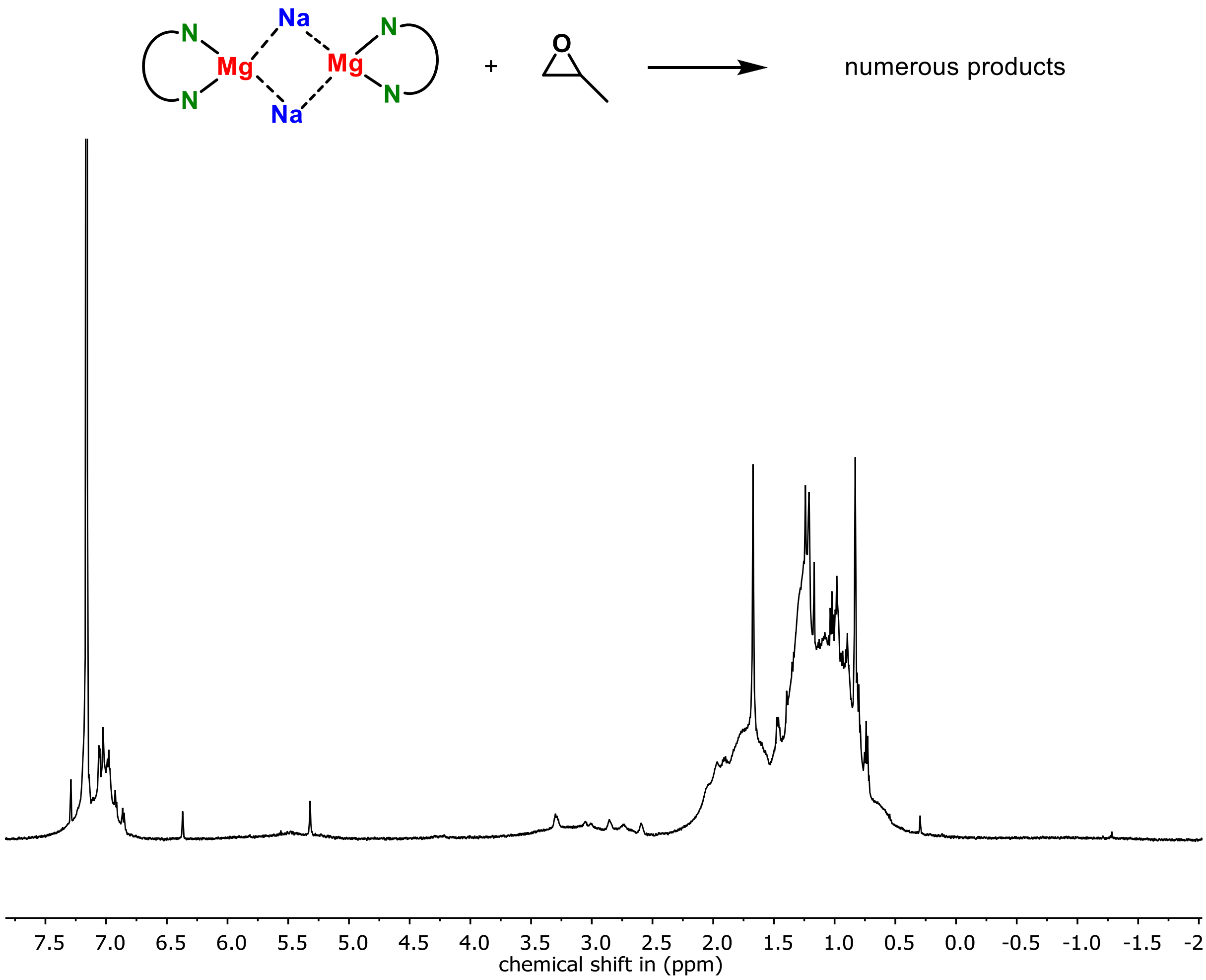


**Figure 60.** $^{1}$H NMR spectrum (600 MHz, 298 K, $C_6D_6$) of the reaction of $[(BDI^*)MgNa]_2$ (**I**) with two equivalents of propylene oxide. Very broad signals and multiplets suggest decomposition and formation of various products.

## 3. Crystal structure determination

Suitable single crystals of compounds **1**, **2**, **4**, **5**, **6**, **7**, **8**, (BDI*)Na(THF) and (BDI*)Na(THF)$_3$ were embedded in protective perfluoropolyalkyether oil (viscosity 1800 cSt; ABCR GmbH) on a microscope slide and a single specimen was selected and subsequently transferred to the cold nitrogen gas stream of the diffractometer.

The intensity data was collected at 100 K using Cu$K_\alpha$ radiation ($\lambda$ = 1.54184 Å) on an Agilent SuperNova dual radiation diffractometer with microfocus X-ray sources and mirror optics. The measured data were processed with the CrysAlisPro software package[1–4]. Data were corrected for Lorentz and polarization effects, and an empirical absorption correction using spherical harmonics as well as a numerical absorption correction based on Gaussian integration over a multifaceted crystal model were applied. Using Olex2[5], the structures were solved by dual-space methods (SHELXT)[6] and refined by full-matrix least-squares procedures on $F^2$ using SHELXL[7]. All non-hydrogen atoms were refined with anisotropic displacement parameters. Most H-atoms were placed in geometrically calculated positions and refined by using a riding model where each H-atom was assigned a fixed isotropic displacement parameter with a value equal to 1.2$U_{eq}$ (CH or $CH_2$) or 1.5$U_{eq}$ ($CH_3$) of its parent C-atom.

Compound (BDI*)MgNa$_3$N''$_2$ (**1**) crystallized with three symmetry independent molecules per asymmetric unit. The crystals were of sub-standard quality and showed some signs of minor twinning. However, attempts to take the twinning into account were unsuccessful. Better results were obtained, when the crystal was treated as a single crystal, but the ignored twinning is likely a contributing factor to one of the observed C-Alerts (PLAT 906) raised by checkCIF. Additionally, disorder was observed for one complete N'' anion, two additional SiMe$_3$ groups and three 3-pentyl moieties. Refinement of the disorder succeeded with the help of similarity restraints (SADI, SIMU) and rigid bond restaints (RIGU)[8], and led to site occupancy factors of 0.762(10)/0.238(10) (N''), 0.68(2)/0.32(2) (SiMe$_3$), 0.579(3)/0.261(3)/0.160(3) (SiMe$_3$), 0.554(8)/0.446(8) (3-pentyl), 0.653(8)/0.347(8) (3-pentyl) and 0.695(6)/0.305(6), respectively.

Disorder was also present in case of (BDI)MgNa$_3$N''$_2$O (**2**). A NaN'' moiety, the methyl groups of a SiMe$_3$ moiety and an additional methyl group were affected. Similarity restraints (SIMU, SADI) were used during refinement, leading to site occupancy factors of 0.855(6)/0.145(6) (NaN''), 0.62(4)/0.38(4) (SiMe$_3$) and 0.721(14)/0.279(14) (Me), respectively.

In case of compound **4** the hydrogen atoms of the OH group and the metallated $CH_2$ group were placed in the positions indicated by a difference electron density map and their positions were refined together with an isotropic displacement parameter. The co-crystallized toluene was disordered and was refined with the help of similarity restraints (SADI, SIMU) and rigid bond restaints (RIGU)[8]. The relative occupancies of the two alternative orientations of the first toluene moiety were refined to 0.770(4) and 0.230(4), while the site occupancy factor of the second toluene moiety was constrained to 0.5 due to its disorder about an inversion center.

Compound (BDI*)MgNa$_3$N''$_2$($O_2$) (**5**) crystallized with three symmetry independent molecules per asymmetric unit.

The structure of (BDI*)MgNa$_4$N''$_3$(N$_2$O$_2$) (**6**) suffered from disorder as well. Here, two 3-pentyl groups adopted two alternative orientations. Refinement of the disorder succeeded with the help of similarity restraints (SADI, SIMU) and rigid bond restraints (RIGU)[8], and led to site occupancy factors of 0.903(4)/0.097(4) (3-pentyl 1) and 0.933(4)/0.067(4) (3-pentyl 2), respectively.

Severe disorder was also detected in case of (BDI*)MgNa$_3$N''$_2$S (**7**). A *t*-butyl group and a 3-pentyl group were found to adopt two alternative positions each, while another 3-pentyl group and one N'' moiety adopted three alternative orientations and for the second N'' anion it was even necessary to model four orientations. A substantial number of similarity restraints (SADI, SIMU) and rigid bond restraints (RIGU)[8], were used during refinement. This treatment led to site occupancy factors of 0.51(4)/0.49(4) (*t*Bu), 0.755(7)/0.245(7) (3-pentyl 1), 0.514(3)/0.343(3)/0.143(3) (3-pentyl 2), 0.4774(18)/0.352(3) 0.170(3) (N'' 1) and 0.329(3)/0.269(3)/0.218(2)/0.184(2) (N'' 2 ), respectively.

The disulfide complex (BDI*)MgNa$_3$N''$_2$(S$_2$) (**8**) is less plagued by disorder than the sulfide derivative. Here, only the two N'' anions and a sodium atom were affected. Use of similarity restraints (SIMU, SADI) enabled a stable refinement. The relative occupancies of the two alternative orientations of all affected moieties were refined to 0.631(11) and 0.369(11).

The THF ligand in the sodium complex (BDI*)Na(THF) was disordered over two alternative orientations. Similarity restraints (SIMU, SADI) and rigid bond restraints[8] were used during refinement, leading to site occupancy factors of 0.60(2) and 0.40(2).

The compound (BDI*)Na(THF)$_3$ crystallized with two symmetry independent molecules per asymmetric unit. While molecule 1 is perfectly ordered, the Na(THF)$_3$ moiety, one C(*t*Bu)Ndipep moiety and two additional 3-pentyl groups of molecule 2 were disordered. The co-crystallized solvent (benzene, *n*-Hexane) was also affected by disorder. Similarity restraints (SADI, SIMU) and rigid bond restraints (RIGU)[8] were applied during refinement in order to achieve a stable refinement. Site occupancy factors of 0.530(7)/0.470(7) (Na(THF)$_3$), 0.748(4)/0.252(4) (C(*t*Bu)Ndipep), 0.824(4)/0.176(4) (3-pentyl 1), 0.846(4)/0.154(4) (3-pentyl 2), 0.573(6)/0.427(6) (*n*-Hexane) and 0.278(3)/0.222(3) (benzene; disorder about an inversion center) were subsequently determined.

The crystal structure data has been deposited with the Cambridge Crystallographic Data Centre. CCDC 2365518-2365522, 2366895, 2366896, 2387079 and 2387080 contain the supplementary crystallographic data for the complexes. This data can be obtained free of charge from The Cambridge Crystallographic Data Centre via www.ccdc.cam.ac.uk/data_request/cif.

Crystallographic and refinement data are summarized in Table S1.

**Table 1.** Crystal data and structure refinement for compounds **1**, **2**, **4**, **5**, **6**, **7**, **8** and related sodium complexes.

| Compound | (BDI*)MgNa$_3$N''$_2$ (**1**) | (BDI*)MgNa$_3$N''$_2$O (**2**) | Complex **4**·1.5Toluene | (BDI*)MgNa$_3$N''$_2$(O$_2$) (**5**) |
|---|---|---|---|---|
| Identification code | hasj220913a | hasj220919a | hasj220203b | hasj231016a |
| Empirical formula | $C_{55}H_{105}MgN_4Na_3Si_4$ | $C_{55}H_{105}MgN_4Na_3OSi_4$ | $C_{107}H_{162}Mg_2N_4Na_2O_2$ | $C_{55}H_{105}MgN_4Na_3O_2Si_4$ |
| Formula weight | 1028.06 | 1044.06 | 1631.00 | 1060.06 |
| Temperature/K | 100.01(10) | 100.0(2) | 100.0(5) | 99.97(10) |
| Crystal system | monoclinic | orthorhombic | triclinic | triclinic |
| Space group | P2$_1$/n | Pca2$_1$ | P-1 | P-1 |
| a/Å | 19.4688(3) | 20.4668(2) | 12.1701(4) | 16.1046(3) |
| b/Å | 22.1144(3) | 14.4504(2) | 12.4334(3) | 19.7957(3) |
| c/Å | 45.7469(5) | 21.7229(3) | 18.6675(5) | 31.4472(4) |
| α/° | 90 | 90 | 79.887(2) | 77.9310(10) |
| β/° | 98.7055(12) | 90 | 75.914(3) | 86.2060(10) |
| γ/° | 90 | 90 | 63.312(3) | 81.7310(10) |
| Volume/Å$^3$ | 19469.0(4) | 6424.62(14) | 2440.70(14) | 9695.2(3) |
| Z | 12 | 4 | 1 | 6 |
| $\rho_{calc}$g/cm$^3$ | 1.052 | 1.079 | 1.110 | 1.089 |
| μ/mm$^{-1}$ | 1.394 | 1.427 | 0.677 | 1.437 |
| F(000) | 6768.0 | 2288.0 | 894.0 | 3480.0 |
| Crystal size/mm$^3$ | 0.45 × 0.308 × 0.065 | 0.151 × 0.095 × 0.078 | 0.285 × 0.194 × 0.097 | 0.37 × 0.167 × 0.085 |
| Radiation | Cu Kα (λ = 1.54184) | Cu Kα (λ = 1.54184) | Cu Kα (λ = 1.54184) | Cu Kα (λ = 1.54184) |
| 2Θ range for data collection/° | 5.256 to 134.158 | 6.116 to 145.39 | 7.982 to 145.336 | 5.55 to 145.03 |
| Index ranges | -23 ≤ h ≤ 23, -19 ≤ k ≤ 26, -54 ≤ l ≤ 39 | -25 ≤ h ≤ 25, -17 ≤ k ≤ 11, -25 ≤ l ≤ 26 | -15 ≤ h ≤ 12, -15 ≤ k ≤ 14, -23 ≤ l ≤ 22 | -19 ≤ h ≤ 19, -24 ≤ k ≤ 15, -38 ≤ l ≤ 38 |
| Reflections collected | 106171 | 29504 | 27510 | 66974 |
| Independent reflections | 34611 [$R_{int}$ = 0.0713, $R_{sigma}$ = 0.0575] | 10974 [$R_{int}$ = 0.0290, $R_{sigma}$ = 0.0330] | 9409 [$R_{int}$ = 0.0301, $R_{sigma}$ = 0.0288] | 37195 [$R_{int}$ = 0.0306, $R_{sigma}$ = 0.0439] |
| Data/restraints/parameters | 34611/2305/2241 | 10974/762/778 | 9409/651/626 | 37195/0/1942 |
| Goodness-of-fit on F$^2$ | 1.022 | 1.038 | 1.034 | 1.042 |
| Final R indexes [I>=2σ (I)] | $R_1$ = 0.0736, w$R_2$ = 0.1907 | $R_1$ = 0.0432, w$R_2$ = 0.1151 | $R_1$ = 0.0473, w$R_2$ = 0.1287 | $R_1$ = 0.0412, w$R_2$ = 0.0990 |
| Final R indexes [all data] | $R_1$ = 0.0960, w$R_2$ = 0.2166 | $R_1$ = 0.0475, w$R_2$ = 0.1189 | $R_1$ = 0.0520, w$R_2$ = 0.1335 | $R_1$ = 0.0504, w$R_2$ = 0.1045 |
| Largest diff. peak/hole / e Å$^{-3}$ | 0.60/-0.39 | 0.52/-0.24 | 0.44/-0.50 | 0.64/-0.38 |
| Flack parameter | - | -0.005(13) | - | - |
| CCDC number | 2365518 | 2365519 | 2365520 | 2365521 |

**Table 1.** Crystal data and structure refinement for compounds **1**, **2**, **4**, **5**, **6**, **7**, **8** and related sodium complexes (continued).

| Compound | (BDI*)MgNa$_4$N''$_3$(N$_2$O$_2$) (**6**) | (BDI*)MgNa$_3$N''$_2$S (**7**) | (BDI*)MgNa$_3$N''$_2$(S$_2$) (**8**) |
|---|---|---|---|
| Identification code | hasj230109a | hasj231120a | hasj230829a |
| Empirical formula | $C_{61}H_{123}MgN_7Na_4O_2Si_6$ | $C_{55}H_{105}MgN_4Na_3SSi_4$ | $C_{55}H_{105}MgN_4Na_3S_2Si_4$ |
| Formula weight | 1271.47 | 1060.12 | 1092.18 |
| Temperature/K | 100.01(10) | 100.01(10) | 99.96(12) |
| Crystal system | monoclinic | monoclinic | monoclinic |
| Space group | P2$_1$/c | P2$_1$/n | P2$_1$/c |
| a/Å | 12.32731(12) | 15.5409(2) | 15.5665(2) |
| b/Å | 28.2360(3) | 17.9637(3) | 18.1360(2) |
| c/Å | 22.09076(18) | 24.7004(4) | 25.0341(3) |
| α/° | 90 | 90 | 90 |
| β/° | 97.3103(9) | 107.336(2) | 106.7690(10) |
| γ/° | 90 | 90 | 90 |
| Volume/Å$^3$ | 7626.72(12) | 6582.42(19) | 6766.94(14) |
| Z | 4 | 4 | 4 |
| $\rho_{calc}$g/cm$^3$ | 1.107 | 1.070 | 1.072 |
| μ/mm$^{-1}$ | 1.645 | 1.676 | 1.924 |
| F(000) | 2776.0 | 2320.0 | 2384.0 |
| Crystal size/mm$^3$ | 0.2 × 0.18 × 0.115 | 0.408 × 0.332 × 0.184 | 0.153 × 0.097 × 0.042 |
| Radiation | Cu Kα (λ = 1.54184) | Cu Kα (λ = 1.54184) | Cu Kα (λ = 1.54184) |
| 2Θ range for data collection/° | 6.26 to 143.748 | 7.728 to 145.208 | 7.678 to 145.158 |
| Index ranges | -11 ≤ h ≤ 14, -32 ≤ k ≤ 34, -26 ≤ l ≤ 26 | -17 ≤ h ≤ 19, -14 ≤ k ≤ 21, -30 ≤ l ≤ 26 | -19 ≤ h ≤ 18, -22 ≤ k ≤ 19, -30 ≤ l ≤ 22 |
| Reflections collected | 26593 | 23684 | 26210 |
| Independent reflections | 14412 [$R_{int}$ = 0.0243, $R_{sigma}$ = 0.0334] | 12591 [$R_{int}$ = 0.0255, $R_{sigma}$ = 0.0330] | 13011 [$R_{int}$ = 0.0285, $R_{sigma}$ = 0.0368] |
| Data/restraints/parameters | 14412/597/848 | 12591/5851/1265 | 13011/497/803 |
| Goodness-of-fit on F$^2$ | 1.023 | 1.058 | 1.013 |
| Final R indexes [I>=2σ (I)] | $R_1$ = 0.0387, w$R_2$ = 0.1003 | $R_1$ = 0.0571, w$R_2$ = 0.1423 | $R_1$ = 0.0341, w$R_2$ = 0.0817 |
| Final R indexes [all data] | $R_1$ = 0.0464, w$R_2$ = 0.1067 | $R_1$ = 0.0718, w$R_2$ = 0.1563 | $R_1$ = 0.0429, w$R_2$ = 0.0864 |
| Largest diff. peak/hole / e Å$^{-3}$ | 0.37/-0.33 | 0.54/-0.41 | 0.57/-0.28 |
| Flack parameter | - | - | - |
| CCDC number | 2365522 | 2387079 | 2387080 |

**Table 1.** Crystal data and structure refinement for compounds **1**, **2**, **4**, **5**, **6**, **7**, **8** and related sodium complexes (continued).

| Compound | (BDI*)Na(THF) | (BDI*)Na(THF)$_3$·0.25Benzene·0.5*n*-Hexane |
|---|---|---|
| Identification code | hasj240220a | hasj211112a |
| Empirical formula | $C_{47}H_{77}N_2ONa$ | $C_{59.5}H_{101.5}N_2NaO_3$ |
| Formula weight | 709.09 | 915.91 |
| Temperature/K | 99.97(11) | 100.0(6) |
| Crystal system | monoclinic | triclinic |
| Space group | $P2_1/n$ | P-1 |
| a/Å | 10.35370(10) | 11.4384(3) |
| b/Å | 19.5644(2) | 15.0170(3) |
| c/Å | 21.7614(2) | 36.1820(7) |
| α/° | 90 | 82.6283(16) |
| β/° | 96.9200(10) | 85.6640(17) |
| γ/° | 90 | 70.183(2) |
| Volume/Å$^3$ | 4375.96(7) | 5795.2(2) |
| Z | 4 | 4 |
| $\rho_{calc}$g/cm$^3$ | 1.076 | 1.050 |
| μ/mm$^{-1}$ | 0.554 | 0.539 |
| F(000) | 1568.0 | 2030.0 |
| Crystal size/mm$^3$ | 0.393 × 0.219 × 0.185 | 0.39 × 0.248 × 0.196 |
| Radiation | Cu Kα (λ = 1.54184) | Cu Kα (λ = 1.54184) |
| 2Θ range for data collection/° | 8.186 to 145.156 | 6.294 to 145.38 |
| Index ranges | -12 ≤ h ≤ 12, -23 ≤ k ≤ 8, -18 ≤ l ≤ 26 | -14 ≤ h ≤ 11, -17 ≤ k ≤ 18, -42 ≤ l ≤ 44 |
| Reflections collected | 16123 | 38638 |
| Independent reflections | 8426 [$R_{int}$ = 0.0207, $R_{sigma}$ = 0.0275] | 22211 [$R_{int}$ = 0.0203, $R_{sigma}$ = 0.0287] |
| Data/restraints/parameters | 8426/224/520 | 22211/3651/1756 |
| Goodness-of-fit on F$^2$ | 1.025 | 1.017 |
| Final R indexes [I>=2σ (I)] | $R_1$ = 0.0367, w$R_2$ = 0.0916 | $R_1$ = 0.0546, w$R_2$ = 0.1493 |
| Final R indexes [all data] | $R_1$ = 0.0412, w$R_2$ = 0.0950 | $R_1$ = 0.0629, w$R_2$ = 0.1577 |
| Largest diff. peak/hole / e Å$^{-3}$ | 0.25/-0.19 | 0.66/-0.53 |
| Flack parameter | - | - |
| CCDC number | 2366895 | 2366896 |

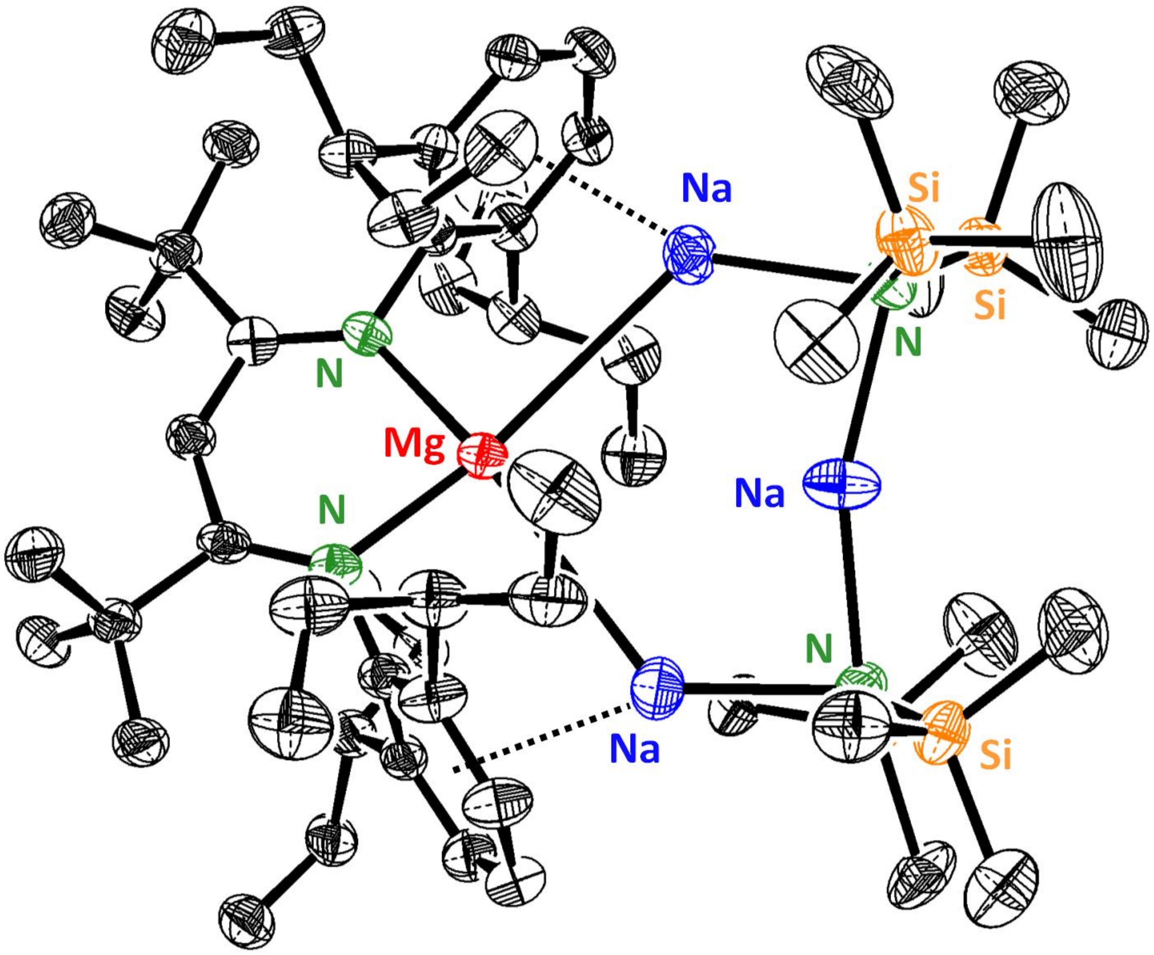


**Figure 61.** ORTEP representation of (BDI*)MgNa$_3$N''$_2$ (**1**) (CCDC 2365518) with atomic displacement ellipsoids set at 50% probability. Hydrogen atoms are omitted for clarity.

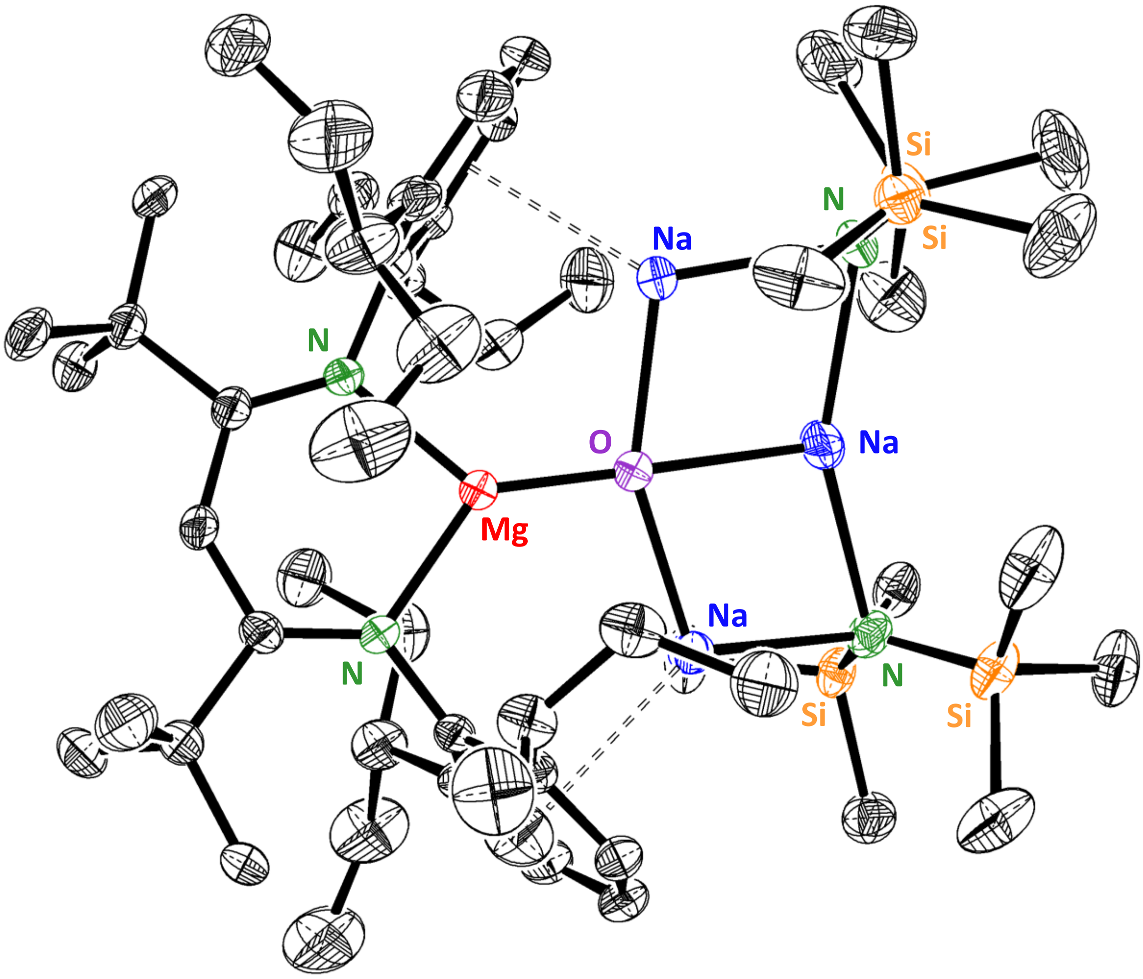


**Figure 62.** ORTEP representation of (BDI*)$MgNa_3N''_2O$ (**2**) (CCDC 2365519) with atomic displacement ellipsoids set at 50% probability. Hydrogen atoms are omitted for clarity.

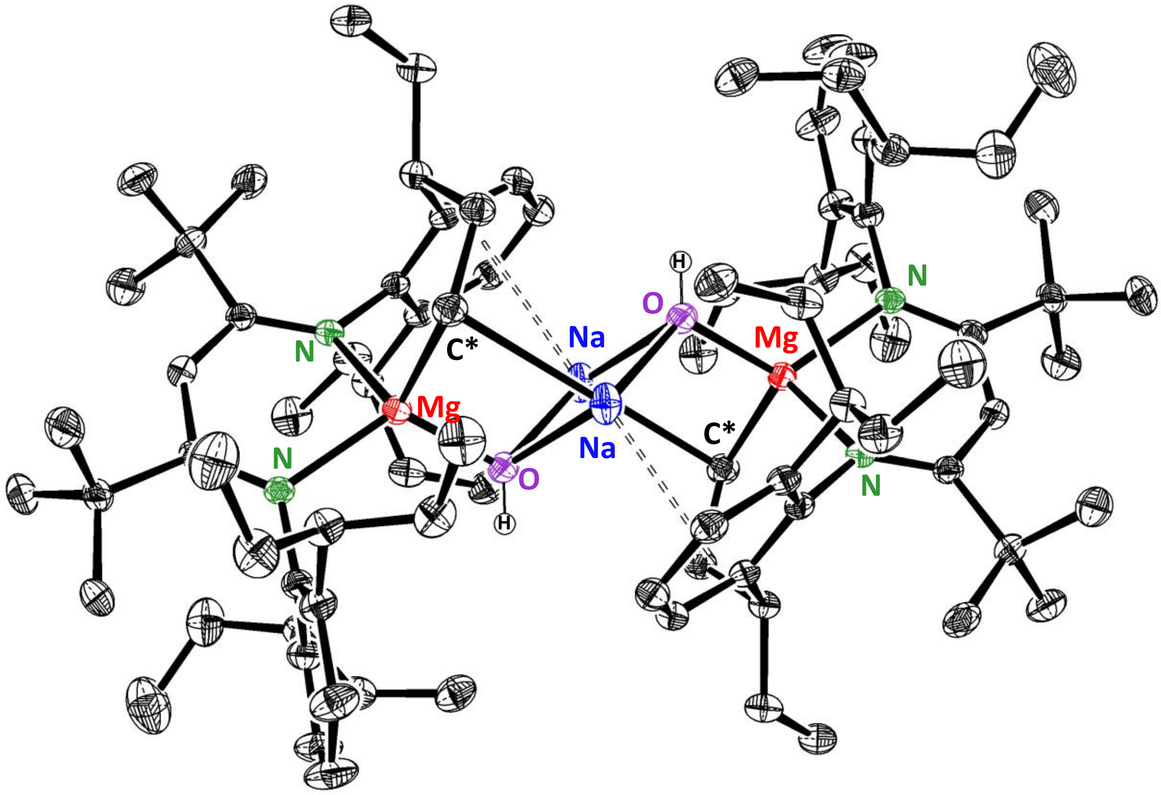


**Figure 63.** ORTEP representation of complex **4** (CCDC 2365520) with atomic displacement ellipsoids set at 50% probability. Hydrogen atoms are omitted for clarity, where appropriate.

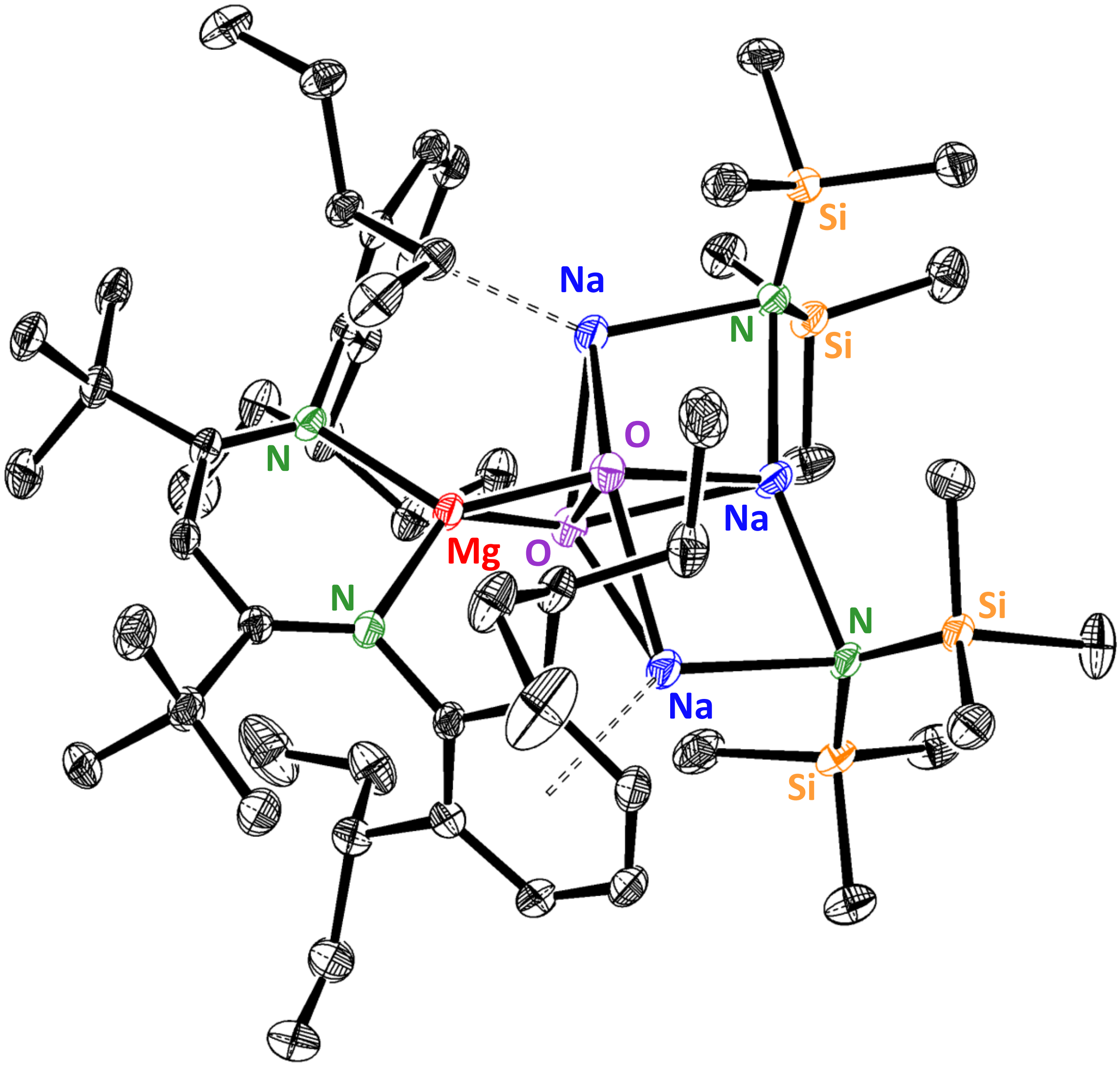


**Figure 64.** ORTEP representation of (BDI*)$MgNa_3N''_2(O_2)$ (**5**) (CCDC 2365521) with atomic displacement ellipsoids set at 50% probability. Hydrogen atoms are omitted for clarity.

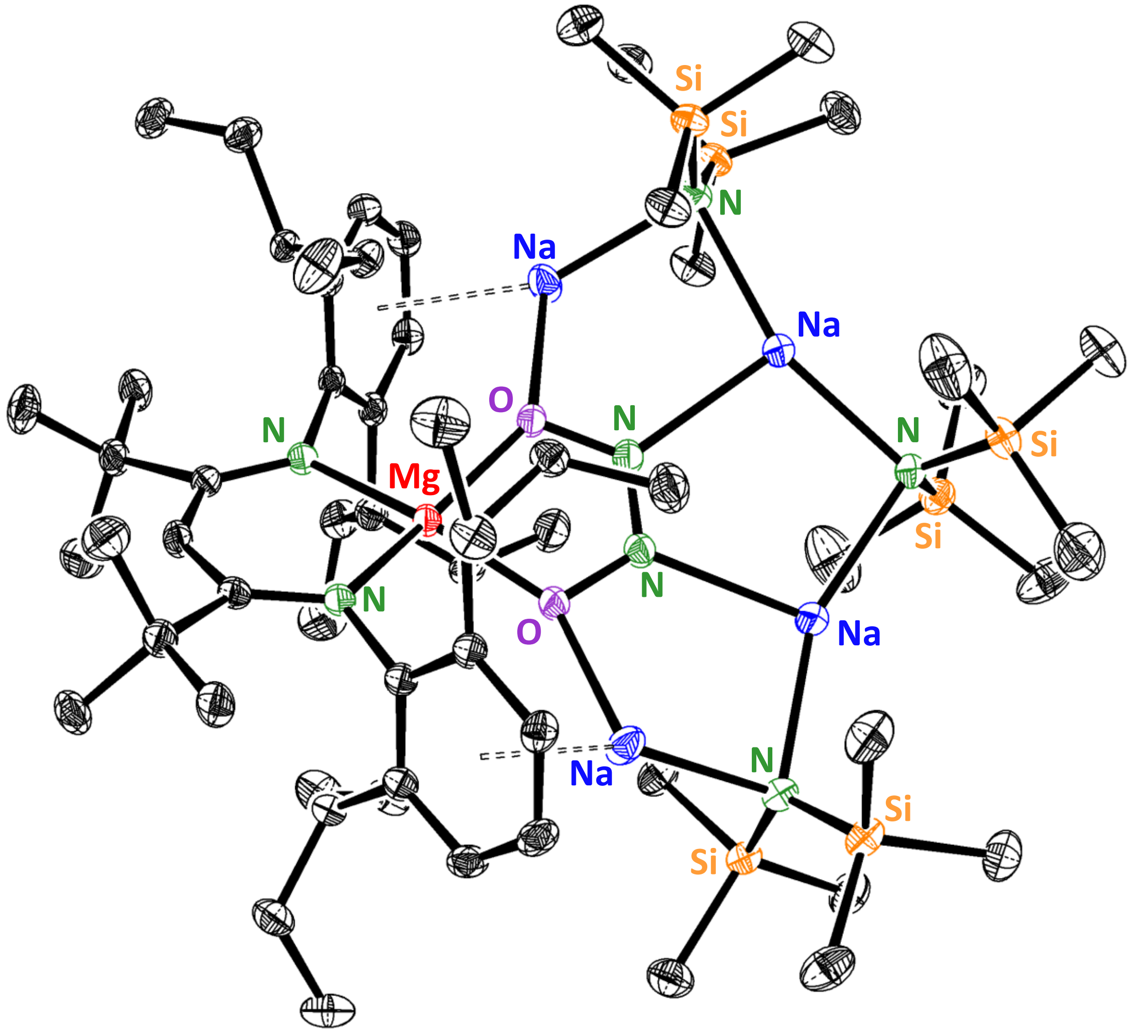


**Figure 65.** ORTEP representation of (BDI*)MgNa$_4$N''$_3$(N$_2$O$_2$) (**6**) (CCDC 2365522) with atomic displacement ellipsoids set at 50% probability. Hydrogen atoms are omitted for clarity.

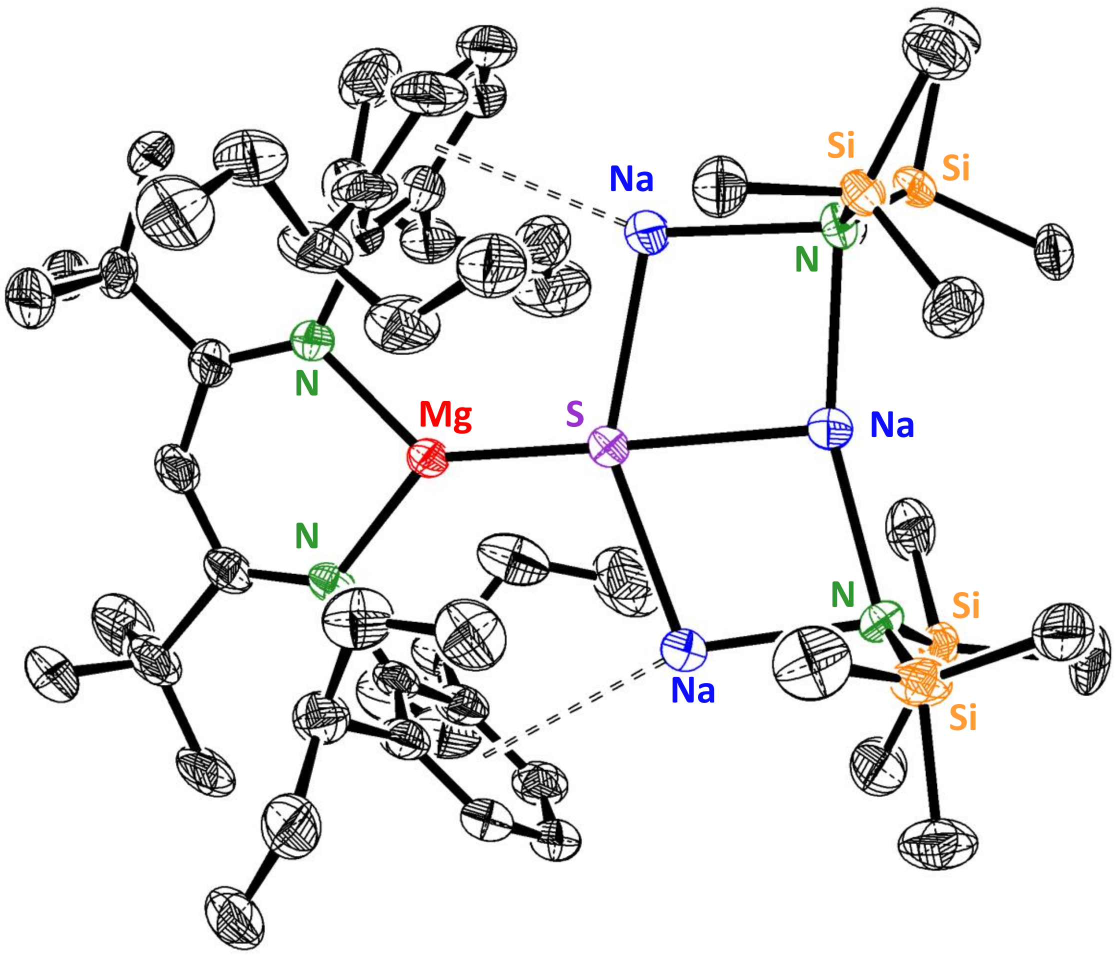


**Figure 66.** ORTEP representation of (BDI*)MgNa$_3$N''$_2$S (**7**) (CCDC 2387079) with atomic displacement ellipsoids set at 50% probability. Hydrogen atoms are omitted for clarity.

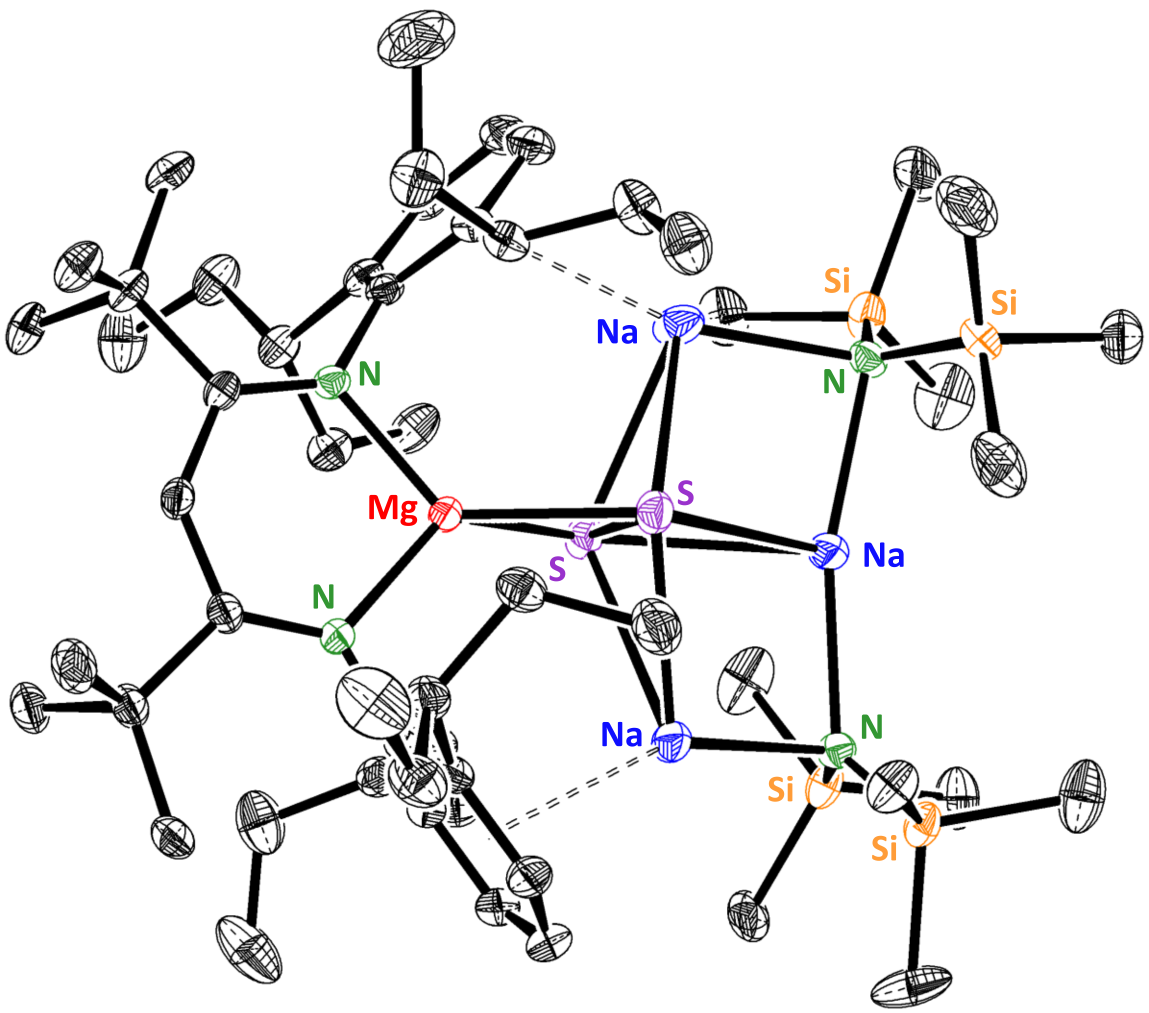


**Figure 67.** ORTEP representation of (BDI*)MgNa$_3$N''$_2$(S$_2$) (**8**) (CCDC 2387080) with atomic displacement ellipsoids set at 50% probability. Hydrogen atoms are omitted for clarity.

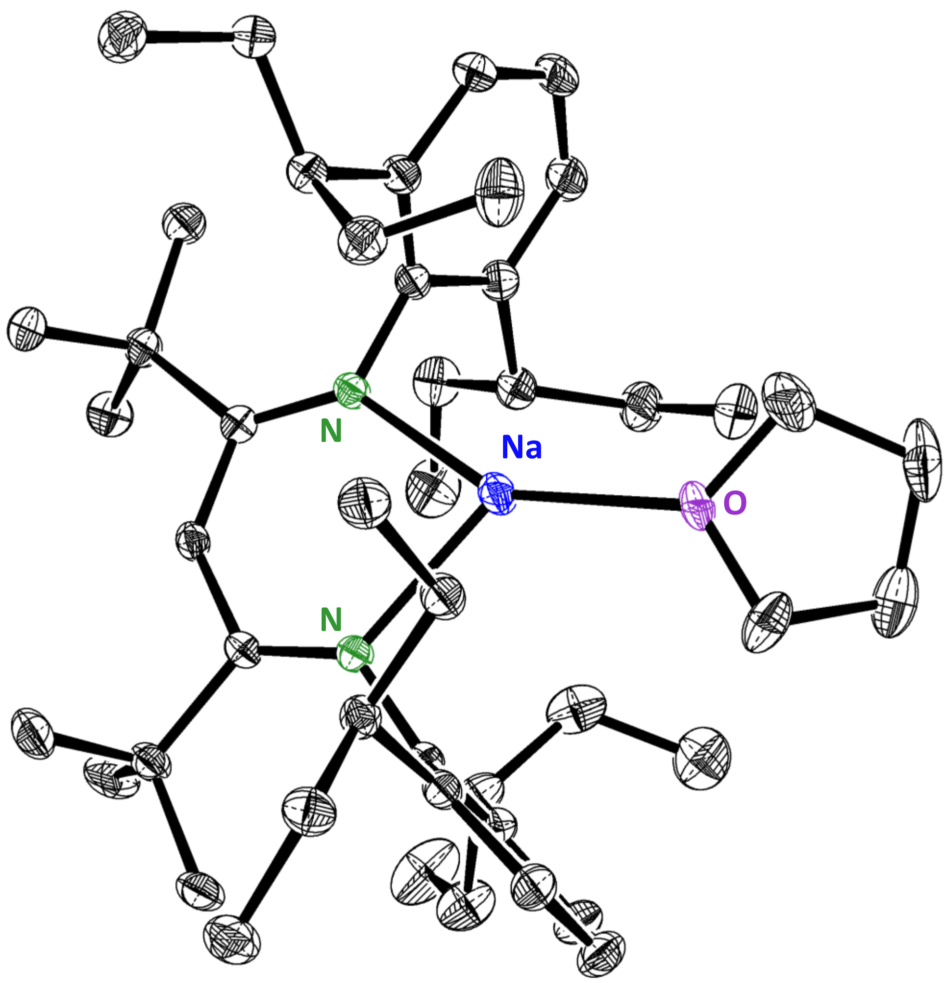


**Figure 68.** ORTEP representation of (BDI*)Na(THF) (CCDC 2366895) with atomic displacement ellipsoids set at 50% probability. Hydrogen atoms are omitted for clarity.

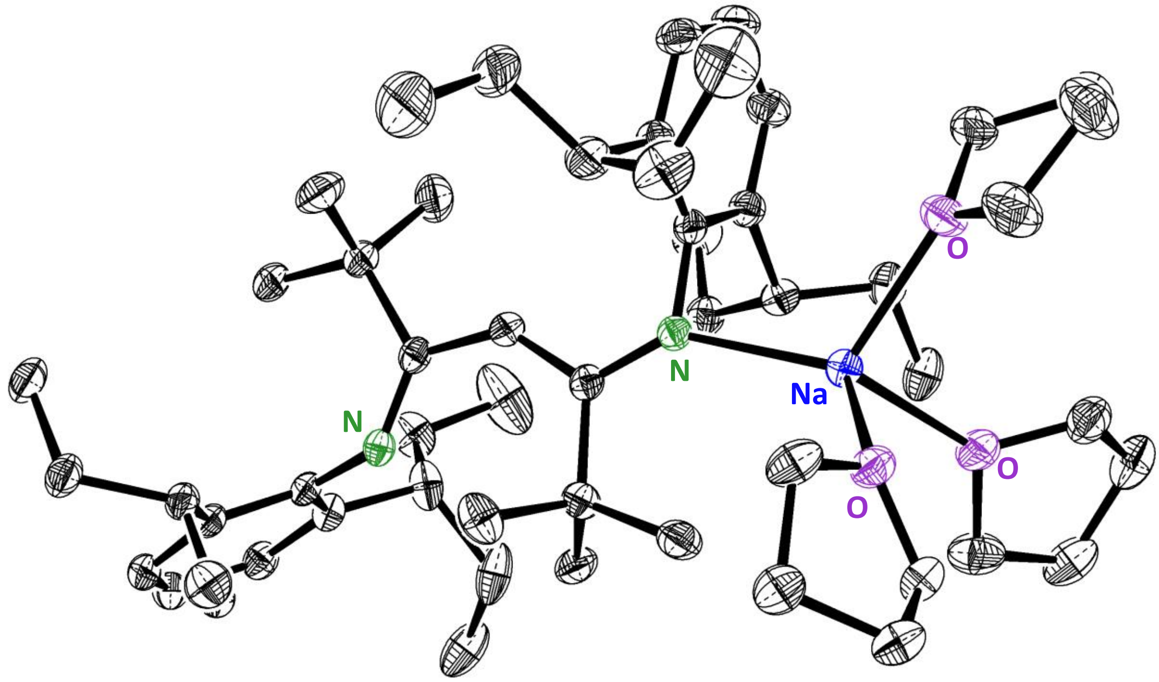


**Figure 69.** ORTEP representation of (BDI*)Na(THF)$_3$ (CCDC 2366896) with atomic displacement ellipsoids set at 50% probability. Hydrogen atoms are omitted for clarity.

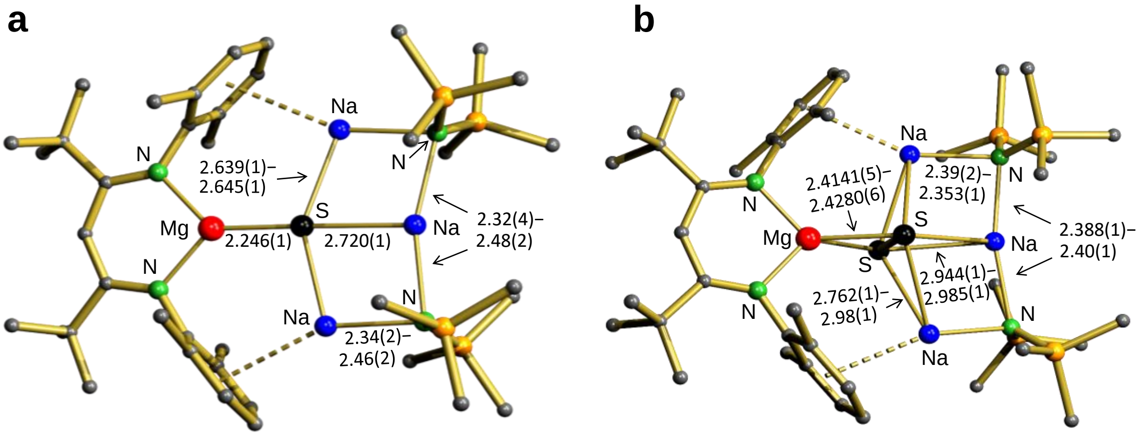


**Figure 70. Crystal structures. a**, (BDI*)MgNa$_3$N''$_2$S (**7**). **b**, (BDI*)MgNa$_3$N''$_2$(S$_2$) (**8**). The Et groups of the Et$_2$(H)C-substituents and H atoms are not shown for clarity. Bond distances shown in Å.

# 4. DFT calculations

All calculations were carried out using Gaussian 16A[9]. All methods were used as implemented. All structures were fully optimized at a B3PW91-GD3BJ/def2svp level of theory which includes Grimme D3 dispersion correction using Becke–Johnson dampening (GD3BJ)[10–14]. All structures were characterized as true minima (Nimag = 0) or as transition states (Nimag = 1) by frequency calculations on the same level of theory. Energies were determined at a B3PW91-GD3BJ/def2tzvp level of theory. The same level of theory was for the NPA charge calculations with NBO6[15]. All structures were evaluated using Molecule 2.3 (ref. [16]). QTAIM analysis was carried out using AIMAll (v17) with the wave functions obtained at the B3PW91-GD3BJ/def2tzvp level of theory[17,18].

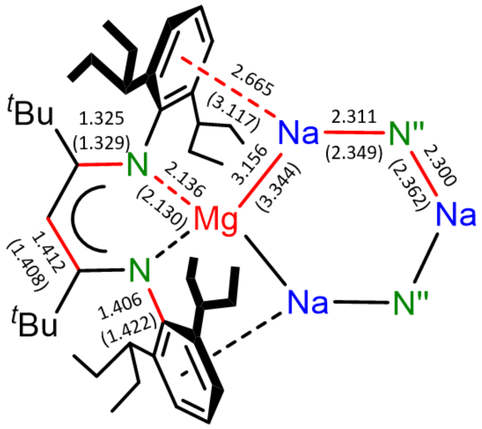


**Figure 71.** Comparison of selected experimentally determined average distances (X-ray) with calculated average values of (BDI*)MgNa$_3$N''$_2$ (**1**).

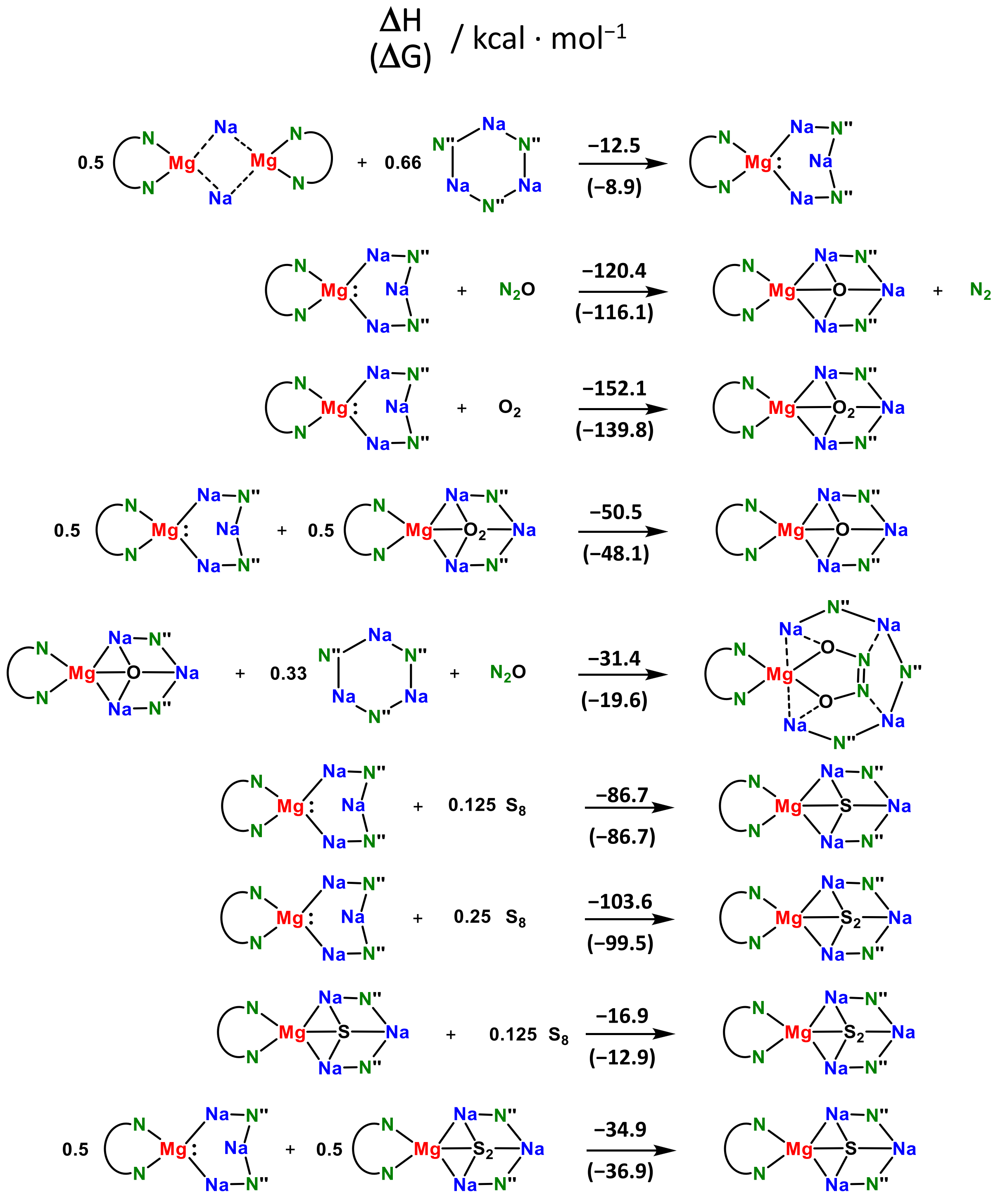


**Figure 72.** Reaction enthalpies (ΔH) and between brackets free energies (ΔG at 298 K) in kcal·mol$^{-1}$.

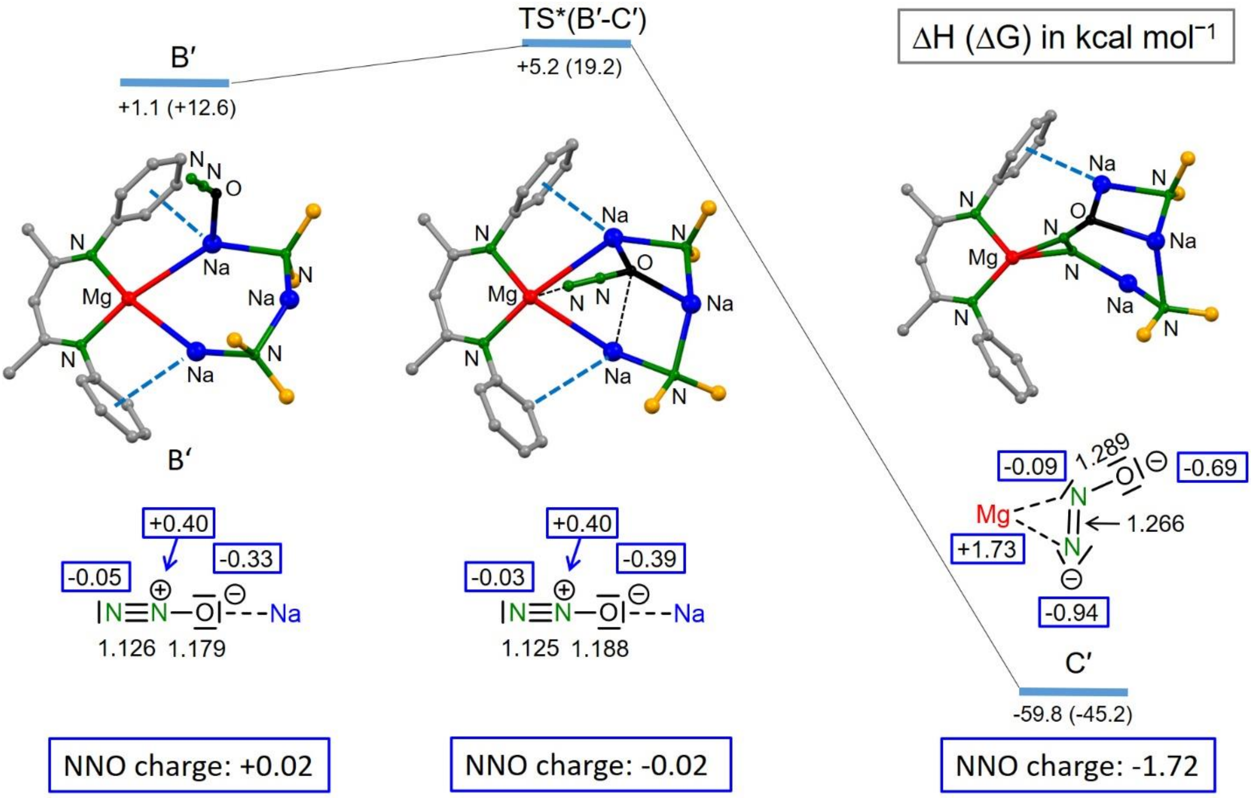


**Figure 73.** Energy profile with ΔH and ΔG values (between brackets) in kcal·mol⁻¹ related to the reactants **1** and $N_2O$. Selected bond lengths in Å and NPA charges in blue boxes. The alternative complex with N=N=O···Na coordination (**B'**) is 0.3 kcal·mol⁻¹ less stable than complex **B** with O=N=N···Na coordination. Complex **C'** represents an alternative for **C**, a complex with $N_2O^{2-}$ encapsulation, but is 21.7 kcal mol⁻¹ less stable. Complex **TS*(B'-C')** represents a very early transition state on the **B'**-**C'** pathway with a very low barrier of only 4.1 kcal·mol⁻¹.

## 5. References


1. Rigaku Oxford Diffraction. *CrysAlisPro Software system, version 1.171.40.84a, Rigaku Corporation, Oxford, UK* (compound **4**, (BDI*)Na(THF)$_3$) (Rigaku, 2020).

2. Rigaku Oxford Diffraction. *CrysAlisPro Software system, version 1.171.41.113a, Rigaku Corporation, Oxford, UK* (compound **1** and **2**) (Rigaku, 2021).

3. Rigaku Oxford Diffraction. *CrysAlisPro Software system, version 1.171.42.72a, Rigaku Corporation, Oxford, UK* (compound **5**, **6**, **7** and **8**) (Rigaku, 2022).

4. Rigaku Oxford Diffraction. *CrysAlisPro Software system, version 1.171.43.106a, Rigaku Corporation, Oxford, UK* (compound (BDI*)Na(THF)) (Rigaku, 2024).

5. Dolomanov, O. V., Bourhis, L. J., Gildea, R. J., Howard, J. A. K. & Puschmann, H. OLEX2: A complete structure solution, refinement and analysis program. *J. Appl. Crystallogr.* **42**, 339–341 (2009).

6. Sheldrick, G. M. SHELXT – Integrated space-group and crystal-structure determination. *Acta Crystallogr. Sect. A Found. Adv.* **71**, 3–8 (2015).

7. Sheldrick, G. M. Crystal structure refinement with SHELXL. *Acta Crystallogr. Sect. C Struct. Chem.* **71**, 3–8 (2015).

8. Thorn, A., Dittrich, B. & Sheldrick, G. M. Enhanced rigid-bond restraints. *Acta Crystallogr. Sect. A Found. Crystallogr.* **68**, 448–451 (2012).

9. Frisch, M. J. *et al.* Gaussian 16 Rev. A.03 (Gaussian, Inc., 2016).

10. Becke, A. D. Density-functional thermochemistry. III. The role of exact exchange. *J. Chem. Phys.* **98**, 5648–5652 (1993).

11. Perdew, J. P. *et al.* Erratum: Atoms, molecules, solids, and surfaces: Applications of the generalized gradient approximation for exchange and correlation. *Phys. Rev. B* **48**, 4978–4978 (1993).

12. Weigend, F. Accurate Coulomb-fitting basis sets for H to Rn. *Phys. Chem. Chem. Phys.* **8**, 1057 (2006).

13. Weigend, F. & Ahlrichs, R. Balanced basis sets of split valence, triple zeta valence and quadruple zeta valence quality for H to Rn: Design and assessment of accuracy. *Phys. Chem. Chem. Phys.* **7**, 3297 (2005).

14. Grimme, S., Ehrlich, S. & Goerigk, L. Effect of the damping function in dispersion corrected density functional theory. *J. Comput. Chem.* **32**, 1456–1465 (2011).

15. Glendening, E. D., Landis, C. R. & Weinhold, F. NBO 6.0 : Natural bond orbital analysis program. *J. Comput. Chem.* **34**, 1429–1437 (2013).

16. van Eikema Hommes, N. J. R. *Molecule* (Erlangen, 2018).

17. Bader, R. F. W. A quantum theory of molecular structure and its applications. *Chem. Rev.* **91**, 893–928 (1991).

18. Keith, T. A. *AIMAll Version 17.01.25* (TK Gristmill Software, 2017).
